# LC-HRMS Coupling to Feature-Based Molecular Networking to Efficiently Annotate Monoterpene Indole Alkaloids of *Alstonia scholaris*

**DOI:** 10.3390/plants14142177

**Published:** 2025-07-14

**Authors:** Ying-Jie He, Yan Qin, Xiao-Dong Luo

**Affiliations:** 1 Key Laboratory of Medicinal Chemistry for Natural Resource, Ministry of Education and Yunnan Province, Yunnan Characteristic Plant Extraction Laboratory, School of Chemical Science and Technology, Yunnan University, Kunming 650500, China; yingjiehe272@163.com (Y.-J.H.); qinyan_yolonda@163.com (Y.Q.); 2 School of Public Health, Kunming Medical University, Kunming 650500, China; 3 School of Environmental Science and Engineering, Southern University of Science and Technology, Shenzhen 518055, China; 4 State Key Laboratory of Phytochemistry and Plant Resources in West China, Kunming Institute of Botany, Chinese Academy of Sciences, Kunming 650201, China

**Keywords:** high-resolution mass spectrometry, feature-based molecular networking, biosynthetic pathways, monoterpene indole alkaloids, *Alstonia scholaris*

## Abstract

Monoterpene indole alkaloids (MIAs) exhibit diverse structures and pharmacological effects. Annotating MIAs in herbal medicines remains challenging when using liquid chromatography combined with high-resolution mass spectrometry (LC-HRMS). This study introduced a new annotation strategy employing LC-HRMS to efficiently identify MIAs in herbal medicines. Briefly, MS^2^ spectra under multiple collision energies (MCEs/MS^2^) helped capture high-quality product ions across a range of mass-to-charge (*m*/*z*) values, revealing key MS^2^ features such as diagnostic product ions (DPIs), characteristic cleavages (CCs), and neutral/radical losses (NLs/RLs). Next, feature-based molecular networking (FBMN) was created to map the structural relationships among MIAs across large MS datasets. Potential MIAs were then graded and annotated through systematic comparison with known biosynthetic pathways (BPs), derived skeletons, and their characteristic substituents. The MCEs/MS^2^-FBMN/BPs workflow was first applied to annotate MIAs in the alkaloids from the leaf of *Alstonia scholaris* (ALAS), a new botanical drug for respiratory diseases. A total of 229 MIAs were systematically annotated and classified, forming a solid basis for future clinical research on ALAS. This study offers an effective strategy that enhances the structural annotation of MIAs within complex herbal medicines.

## 1. Introduction

Liquid chromatography combined with high-resolution mass spectrometry (LC-HRMS) is widely used for identifying organic molecules in complex matrices, such as phytochemicals [1,2,3,4], food compositions [5], chemical migrants [6], drug metabolites [7], and endogenous substances [8]. However, there are still significant challenges in confidently annotating structures in complex herbal medicines, which are crucial for better understanding the biological functions or effects of phytochemicals in organisms.

Ultrahigh-performance liquid chromatography electrospray ionization quadrupole time-of-flight mass spectrometry (UHPLC-ESI-QTOF-MS^E^) is an advanced LC-HRMS platform that generally involves three data processing steps for structural analysis identification [1,2,6]. Firstly, establishing suitable chromatographic separation conditions for complex components is fundamental for covering most compounds, improving ionization efficiency, and reducing ionization competition. Next, the MS^1^ mass spectrum provides accurate mass (AM) support based on qualitative scores that mainly include precise mass-to-charge (*m*/*z*) ratios, isotope matches (abundance and spacing), and alternative molecular formulas, as well as indicating retention time (RT). The final step involves analyzing the MS^2^ mass spectrum (also called MS/MS) to convert molecular formulas into structures by examining fragment patterns, such as diagnostic product ions (DPIs), characteristic cleavages (CCs), and neutral/radical losses (NLs/RLs). This stage is crucial for proposing a confident structure from multiple candidates.

Experimental practices suggest that MS^2^ spectra under multiple collision energies (MCEs/MS^2^) enable capturing ions across a wide *m*/*z* range. This range facilitates the detection of most fragment ions with a threshold, resulting in higher mass scores, accurate molecular formulas, and reliable fragment patterns such as DPIs, CCs, and NLs/RLs. These are crucial for increasing confidence in the structural annotation of compounds. The next challenge is efficiently marking and identifying analogs within complex MS datasets of target samples. Traditionally, the analytical process involves comparing retention behavior, molecular weight, and spectral similarities or differences with standard substances or literature reports. However, a single MS experiment can produce thousands of MS^2^ spectra within minutes [6], and a project can generate millions of spectra. This creates enormous difficulties for manual analysis of these large datasets [9,10], making systematic annotation time-consuming and labor-intensive for experts.

Global natural products social molecular networking (GNPS) addresses this gap. GNPS is a data-driven platform for storing, analyzing, and sharing knowledge about MS^2^ spectra that allows community sharing of raw spectra, ongoing annotation of deposited data, and collaborative curation of reference spectra (called spectral libraries) and experimental data [9]. Molecular networking (MN) serves as the foundation for the web-based MS infrastructure of GNPS, and each of these tens of millions of MS^2^ spectra processed by MN is automatically compared to reference spectral libraries to identify compounds. The similarity of MS^2^ spectra reflects the similarity of chemical structures in identifying potential molecules; chemical structural information can thus be represented as a network, and chemical relationships can be visualized [10]. The enhanced feature-based MN (FBMN) utilizes the capabilities of well-established MS processing software and improves traditional MN by incorporating not only MS^1^ information, such as isotope patterns and RT, but also ion mobility separation when performed [9]. FBMN enables precise visualization of isomeric or closely related compounds within molecular families, offering a promising alternative to fast mark analogs in complex herb matrices.

Monoterpene indole alkaloids (MIAs), an essential class of active molecules in herbal medicines, have multiple pharmacological activities in clinical use. For example, vinca alkaloids, first isolated from *Catharanthus roseus*, have been developed as microtubule-targeting agents (Vincristine, Vinblastine, Vinorelbine, Vindesine, Vinflunine, etc.) approved for treating hematological and lymphatic cancers [11]. Reserpine is initially derived from Rauvolfia serpentina and is used as a central nervous system depressant. It blocks the postganglionic nerve fiber from releasing the excitatory neurotransmitter norepinephrine in the synaptic gap [12].

MIAs are naturally produced by the condensation of tryptamine and secologanin via the Pictet–Spengler reaction [13]. They mainly possess molecular weights from 300 to 400 Da and display diversity in chemical structures due to multiple chiral carbons. This results in the prevalence of MIA isomers, analogs, or derivatives with similar structures in herbs, which still makes it difficult to quickly annotate MIAs in complex components using the MCEs/MS^2^ coupling with the FBMN strategy. Additionally, potential MIAs can also be derived from basic skeletons and typical substituents based on the biosynthetic pathways (BPs) of the target herbs to enable more efficient annotation.

*Alstonia scholaris* is a herb with leaves rich in MIAs that are widely used in Chinese traditional medicine to treat respiratory diseases, such as cough, asthma, phlegm, and chronic obstructive pulmonary disease [14,15]. The alkaloids from the leaf of *A. scholaris* (ALAS), a new investigational botanical drug (No. 2011L01436) for respiratory disease, have been confirmed to contain the major MIAs (e.g., scholaricine, 19-*epi*-scholaricine, vallesamine, and picrinine) for anti-inflammatory and analgesic effects [16], anti-tussive, anti-asthmatic, and expectorant activities [17], anti-airway inflammation [18] and anti-allergic asthma effects [15]; protective activity against emphysema [18] and post-infectious cough [18], the ability to alleviate pulmonary fibrosis [14], anti-microbial effects [19], and inhibition of influenza A virus replication [20]. ALAS has been approved for phase I/II clinical trials by the China Food and Drug Administration (CFDA).

This study aims to comprehensively annotate MIAs in ALAS using the proposed MCEs/MS^2^-FBMN/BP workflow (Figure S1). Fragment patterns of 48 reference MIAs (ten subtypes) were systematically illustrated in detail, and DPIs, CCs, and NLs/RLs were proposed accordingly. The non-target captured LC-MS^2^ dataset of ALAS was processed with MZmine 2 to screen MS^2^ features of MIAs, and the FBMN was successfully constructed. The potential structures of MIAs were derived from basic MIA skeletons and typical substituents based on the reported BPs of the *Alstonia* genus, as well as consulted from the in-house database, SciFinder, online libraries, and the literature. Consequently, 229 known and unknown MIAs in ALAS were interactively annotated and classified for the first time. This study provides a fundamental basis for further clinical investigations of ALAS and offers an LC-HRMS-based strategy and clues for annotating MIAs in herbal medicines.

## 2. Results and Discussion

### 2.1. Characteristic Fragment Patterns of MIAs

MIAs were the most abundant metabolites in *A. scholaris*, biosynthesized through the condensation of tryptophan with secologanin [13]. MIAs showed higher ion intensities in the positive ESI mode than in the negative mode, with the hydrogenated adduct [M+H]^+^ being clearly observed, as shown in Figure 1. An in-house database containing MCEs/MS^2^ spectra of 48 reference MIAs, including scholaricine-type (10), picrinine-type (6), vallesamine-type (4), alstolactine-type (7), yohimbine-type (2), alstoscholarisine-type (7), scholarisine-type (3), vallesiachotamine-type (2), alstoscholarine-type (2), and unclassified MIAs (5), was successfully built and accessed in the GNPS library with specific spectrum IDs (Figure 2, Table S1, Figures S2–S49). Additionally, MS^2^ features for each MIA subtype (Figure 3, Figure 4 and Figure 5 and Figures S50–S56) and common NLs/RLs (Table S2) were tentatively proposed. The main MIA subtypes, including scholaricine-type, picrinine-type, vallesamine-type, and alstolactine-type, were used as examples to summarize characteristic fragment patterns of MIAs.

#### 2.1.1. Scholaricine-Type MIAs

Scholaricine-type MIAs including scholaricine (**P41**), 19-*epi*-scholaricine (**P45**), alstoniascholarine F (**P36**), alstoniascholarine H (**P70**), scholaricine N-oxide (**P61**), alstoniascholarine P (**P66**), N-demethylalstogustine N-oxide (**P109**), alstoniascholarine Q (**P143**), tubotaiwine (**P162**), and tubotaiwine N-oxide (**P184**) showed [M+H]^+^ as the precursor ion (**P41**, **P45**, **P70**, and **P109**: *m*/*z* 357.1809 [C_20_H_25_N_2_O_4_]^+^; **P36**: *m*/*z* 327.1703 [C_19_H_23_N_2_O_3_]^+^; **P61** and **P66**: *m*/*z* 373.1758 [C_20_H_25_N_2_O_5_]^+^; **P143**: *m*/*z* 387.1914 [C_21_H_27_N_2_O_5_]^+^; **P162**: *m*/*z* 355.1652 [C_20_H_23_N_2_O_4_]^+^; **P184**: *m*/*z* 341.1860 [C_20_H_25_N_2_O_3_]^+^).

**P41**, **P45**, **P36**, and **P70** were typical scholaricine-type MIAs that differ in the absolute configuration of C-19/C-20 and the substituents at C-12 and C-16. As shown in Figure 3, these examples illustrated the common fragment patterns of classical scholaricine-type MIAs: (1) the DPI [M+H-CH_4_O/H_2_O]^+^ with high intensity resulted from the incomplete elimination of COOCH_3_ or COOH groups on the C-16 position; (2) a pair of DPIs, [M+H-CH_4_O/H_2_O-H_2_O]^+^ and [M+H-CH_4_O/H_2_O-C_4_H8O]^+^, were formed by the elimination of the OH group at C-19 and ring D cleavage, respectively; (3) a DPI, [M+H-CH_4_O/H_2_O-C_4_H_8_O-CO]^+^, was produced through NL of CO (27.99 Da) from [M+H-CH_4_O/H_2_O-C_4_H_8_O]^+^; and (4) the DPI [M+H-CH_4_O/H_2_O-C_4_H_8_O-C_2_H_5_N]^+^ resulted from ring E cleavage, which corresponds to the indole ring skeleton of scholaricine-type MIAs.

**P61**, **P66**, **P109**, and **P143** differed in the substituent at the C-12 position and were used to analyze common fragment patterns of scholaricine-type N4-oxide MIAs (Figure 3): (1) an abundance of DPI [M+H-CH_4_O]^+^ was generated by NL of CH_4_O (32.03 Da) from the COOCH_3_ group on the C-16 position; (2) a specific DPI [M+H-•C_6_H_14_NO_2_]^•+^ was produced after simultaneous ring D/E cleavage; (3) the DPI [M+H-122.05 Da]^+^, corresponding to the indole ring skeleton of scholaricine-type N-oxide MIAs, was generated through successive NLs of CH_4_O+H_2_O+C_2_H_4_O+CO (122.05 Da); and (4) a pair of ring D DPIs, *m*/*z* 82.06 ([C_5_H_8_N]^+^) and *m*/*z* 94.06 ([C6H8N]^+^), resulted from a_1_- and a_2_-cleavage on the fragment ion [M+H-CH_4_O-H_2_O-C_2_H_4_O]^+^, respectively.

Due to the rotation of the single bond between N-4 and C-5 during biosynthetic pathways, **P162** and its N4-oxide compound (**P184**) exhibited an ethyl chain at the C-14 position. Although the fragment patterns of **P162** and **P184** did not match the typical scholaricine-type MIAs, the characteristic NLs, including CO (27.99 Da), H_2_O (18.01 Da), C_2_H_2_, C_2_H_4_, C_2_H_5_N, and C_3_H_7_N, were detected in their MS^2^ spectra.

#### 2.1.2. Picrinine-Type MIAs

Six standard picrinine-type MIAs, including picrinine (**P160**), picralinal (**P169**), burnamine (**P96**), 5*α*-methoxystrictamine (**P150**), strictamine N-oxide (**P139**), and scholarisine B (**P202**), showed [M+H]^+^ as the precursor ion (**P160** and **P139**: *m*/*z* 339.1703 [C_20_H_23_N_2_O_3_]^+^; **P169**: *m*/*z* 367.1652 [C_21_H_23_N_2_O_4_]^+^; **P96**: *m*/*z* 369.1809 [C_21_H_25_N_2_O_4_]^+^; **P150**: *m*/*z* 353.1860 [C_21_H_25_N_2_O_3_]^+^; **P202**: *m*/*z* 411.1914 [C_23_H_27_N_2_O_5_]^+^).

As shown in Figure 4, the compound **P160** was used as an example to illustrate common fragment patterns of picrinine-type MIAs: (1) three concurrent DPIs with *m*/*z* 108.08 ([C_7_H_10_N]^+^), 107.07 ([C_7_H_9_N]^•+^), and 106.06 ([C_7_H_8_N]^+^), generated through b_1_-cleavage, helped characterize the structure of ring D; (2) a specific DPI [M+H-H_2_O]^+^ appeared if an oxygen bridge existed between C-2 and C-5; (3) a DPI with *m*/*z* 120.08 ([C_8_H_10_N]^+^), produced by b_2_-cleavage on [M+H-H_2_O]^+^, also aided in characterizing ring D; (4) a DPI with *m*/*z* 144.08 ([C_10_H_10_N]^+^), corresponding to the indole ring skeleton, resulted from the simultaneous removal of ring D and the carbon bridge between C-7 and C-18 from [M-H-H_2_O]^+^; and (5) a pair of DPIs with *m*/*z* 307.18 ([M+H-CH_4_O]^+^) and *m*/*z* 279.15 ([M+H-CH_4_O-CO]^+^), generated by NL of CH_4_O (32.03 Da) and CH_4_O+CO (60.02 Da) from [M+H]^+^, could be used to identify the COOCH_3_ group at C-16. 

Comparisons with **P169** and **P96** showed that C-16 contained a CHO and CH_2_OH group, respectively, leading to a common DPI at *m*/*z* 339.17 ([C_20_H_23_N_2_O_3_]^+^) in the MS^2^ spectrum of both **P169** and **P96**, resulting from NL of CO and CH_2_O. For **P150**, which featured a substitution of an OCH_3_ group at C-5 and lacked an oxygen bridge between C-2 and C-5, a pair of DPIs at *m*/*z* 122.09 ([C8H12N]^+^) and 120.08 ([C_8_H_10_N]^+^) were formed by b_2_-cleavage. In the case of **P202**, a DPI at *m*/*z* 178.08 ([C_10_H_12_NO_2_]^+^) was produced by b_1_-cleavage, corresponding to two unique side chains at C-20 and C-21 of ring D. **P139** was a picrinine-type N4-oxide MIA lacking an oxygen bridge between C-2 and C-5. Notable DPIs of **P139** included (1) three ions at *m*/*z* 323.18 ([M+H-O]^+^), 322.17 ([M+H-•OH]^+^), and 321.16 ([M+H-H_2_O]^+^); and (2) a DPI at *m*/*z* 121.08 ([C8H11N]^•+^) resulting from b_2_-cleavage after NL of H_2_O (18.01 Da).

#### 2.1.3. Vallesamine-Type MIAs

Four vallesamine-type alkaloid standards, including vallesamine (**P91**), vallesamine N-oxide (**P116**), alstoniascholarine (**P53**), and 6,7-seco-angustilobine B (**P137**), exhibited [M+H]^+^ as the precursor ion under an ESI source (**P91** and **P137**: *m*/*z* 341.1860 [C_20_H_25_N_2_O_3_]^+^; **P116**: *m*/*z* 357.1809 [C_20_H_25_N_2_O_4_]^+^; **P53**: *m*/*z* 327.1703 [C_19_H_23_N_2_O_3_]^+^).

As shown in Figure 5, **P91** and **P116** served as examples to illustrate common fragment patterns of vallesamine-type MIAs: (1) the DPI *m*/*z* 232.09 ([C_13_H_14_NO_3_]^+^) and its two complementary ring D DPIs (**P91**: *m*/*z* 110.09 and 108.08; **P116**: *m*/*z* 126.09 and 124.07) were produced by c_1_-cleavage; (2) the NL of H_2_O generated a DPI *m*/*z* 214.09 ([C_13_H_12_NO_2_]^+^) (18.01 Da) from *m*/*z* 232.09, indicating the OH group was substituted at the C-17 position; and (3) the DPI *m*/*z* 182.06 ([C_12_H_8_NO]^+^) and the indole ring skeleton DPI *m*/*z* 154.06 ([C_11_H_8_N]^+^) were produced by the NL of CH_4_O (32.03 Da) and CH_4_O+CO (60.02 Da) from *m*/*z* 214.09, which could be used to identify the COOCH_3_ group at the C-16 position.

For compound **P53**, a specific DPI at *m*/*z* 283.18 ([M+H-CO_2_]^+^) was produced due to a COOH group substituted at the C-16 position; a DPI at *m/z* 174.09 ([C_11_H_12_NO]^+^), along with its two complementary ring D DPIs at *m*/*z* 110.09 ([C_7_H_12_N]^+^) and *m*/*z* 108.08 ([C_7_H_10_N]^+^), were formed through c_1_-cleavage at *m*/*z* 283.18. Additionally, DPIs at *m*/*z* 110.09 and *m*/*z* 108.08 could also result from c_1_-cleavage at the precursor ion.

**P137** was a peculiar vallesamine-type MIA, and its fragment patterns are shown in Figure 5: (1) two complementary DPIs at *m*/*z* 202.06 ([C_12_H_12_NO_2_]^+^) and 140.10 ([C_8_H_14_NO]^+^) resulted from c_2_-cleavage; (2) an indole ring skeleton DPI at *m*/*z* 142.12 ([C_10_H_8_N]^+^) arose from the complete loss of the COOCH_3_ group on *m*/*z* 202.06; (3) a diagnostic ring D ion at *m*/*z* 110.10 ([C_7_H_12_N]^+^) formed by the NL of CH_2_O (30.01 Da) from *m*/*z* 140.10; and (4) the DPI at *m*/*z* 224.13 ([C_12_H_18_NO_3_]^+^) was caused by the NL of an indole molecule (cleavage of the single bond between C-2 and C-16) from [M+H]^+^.

### 2.2. Feature-Based Molecular Networking (FBMN) of MIAs in ALAS

The datasets in the “.mZML” format were initially imported into MZmine 2, and most steps followed the general workflow shown in previous studies [21,22]. The simplified tutorial is provided in Figure S57. After completing the basic processing steps, the most important part of this study focused on screening MIAs’ MS^2^ features. Therefore, the molecular weight was set in the range of 180 Da to 500 Da, elemental composition was limited to C 0–50, H 0–80, O 0–30, and N 0–2, and the DBE was constrained to 5–20. The charge of the precursor ion was set to one, and molecular formulas with ppm < 2 were accurately generated. Finally, RT-consistent MS^2^ features were manually compared to filter out false features caused by in-source collision-induced dissociation (IS-CID). The “.csv” and “.mgf” files were exported to GNPS. Then, FBMNs with different cosine thresholds were created, enabling efficient visualization of isomeric and similar MIAs in LC–MS^2^ datasets of ALAS [9]. In summary, a highly restrictive FBMN (cosine 0.70) was first generated to create a network with tightly clustered, highly correlated nodes, facilitating rapid identification of their characteristic MS^2^ fragment patterns. Subsequently, moderate-restriction FBMNs (cosine at 0.40, 0.50, 0.58) were produced, forming an expanded network that connected some unlinked nodes within clusters for further annotation and classification (Figure S58) [23].

The FBMN (cosine 0.58) displayed the overall distribution of major clusters that were systematically analyzed (Figure 6). Compounds with similar structures were automatically grouped into clusters. The precursor ion intensity of some MIAs was too low to be detected in ALAS, which prevented them from reaching the threshold to trigger MS^2^ spectrum collection. A total of 30 reference MIAs were automatically annotated in the ALAS FBMN. For instance, **P41** (node 7), **P160** (node 86), **P91** (node 4), **P220** (node 148), **P179** (node 126), and **P225** (node 34) were accurately identified as scholaricine, picrinine, vallesamine, scholarisine M, alstoscholarisine C, and vallesiachotamine, respectively (Figure S59). Additionally, some nodes matched more closely with higher cosine values in GNPS Libraries; for example, **P107** (node 31), **P77** (node 25), **P29** (node 84), **P186** (node 193), and **P161** (node 1) were effectively identified as yohimbine, akuammidine, echitamidine, leuconolam, and pleiocarpamine, respectively (Figure S60). In summary, 18 MIAs were precisely annotated based on the reference standards. The main clusters primarily contained six subtypes of MIAs, including scholaricine-type, picrinine-type, vallesamine-type, yohimbine-type, alstolactine-type, and alstoscholarisine-type MIAs (Figure S58).

FBMN is an essential tool that enables the targeted isolation of new chemical entities and allows for accurate isomer discrimination. In recent years, using FBMN technology has helped researchers discover an increasing number of natural products with unprecedented structural frameworks and notable bioactivities, transforming the approach to natural product isolation and discovery. For example, Padilla-González et al. used FBMN to analyze the structural differences in caffeic acid esters systematically and successfully isolated new, low-abundance derivatives from various tissue extracts of *Smallanthus sonchifolius* [24]; Cauchie et al. combined molecular networking and substructure annotation to guide the isolation of MIAs (inaequalisines A and B) from the roots of *Callichilia inaequalis* [25]. The previous research isolated a limited number of MIAs in the *Alstonia* genus, which indicated the urgent need for a more comprehensive understanding of its chemical composition, especially regarding the unknown MIAs. One effective approach to achieve this is by using FBMN strategies, which leverage MS^2^ data to quickly cluster and remove duplicate compounds with similar structures. By using known MIAs as “seeds” to deduce the presence of unknown ingredients [26], FBMN can help us discover previously unreported components such as scholaricine-type MIAs. This approach can offer valuable insights into the unique MIA profile of the *Alstonia* genus and its potential medicinal benefits. While the FBMN platform has shown promising results in analyzing MIAs, the study recognized some limitations of this method. For instance, the clustering of different MIA subtypes suggested that their MS^2^ spectra were somewhat similar, making it difficult for researchers to quickly distinguish their structural differences. However, these findings indicated the potential close relationships among these MIAs in BPs, which may help in the practical annotation of unknown MIA nodes.

### 2.3. Potential MIAs in the Biosynthetic Pathways of the Alstonia Genus

For efficient annotation of MIAs in ALAS, this study constructed hypothetical BPs of *Alstonia* MIAs based on the verified and reported chemical literature, serving as a key reference for annotating MIAs in FBMN (Figure S61). Partially characteristic biosynthetic pathways are shown in Figure 7. Briefly, tryptophan and secologanin first formed the precursor strictosidine, which undergone ed s biochemical reactions such as deglycosylation, dehydration, reduction, and oxidation to produce yohimbine-type MIAs like ajmalicine and 19*E*-geissoschizine. The precursor 4,21-dehydro-geissoschizine, from which 19*E*-geissoschizine was derived, marked the start of the first branch’s differentiation; this pathway led to (+)-stemmadenine through complex reactions, and produced vallesamine, a typical vallesamine-type MIA. Vallesamine can be oxidized to vallesamine-N-oxide or hydrolyzed to form alstoniascholarine A. It can also undergo oxidation, reduction, and cyclization after N-4 methylation to generate alstoscholarisine-type MIAs, including alstoscholarisine I, C, and H. The second branch began with 19*E*-geissoschizine, which cyclized to form rhazimol. This intermediate can then be oxidized, methylated, and processed through various reactions to produce picrinine-type MIAs such as burnamine, picralinal, 5*α*-methoxystrictamine, and picrinine. Subsequently, picrinine can undergo N-4 methylation, ring cleavage, oxidation, and epoxidation, resulting in alstolactin-type MIAs like scholarisine M, L, and E. The third branch, also starting from 19*E*-geissoschizine, produced preakuammicine via geissoschizine oxidase activity, forming the core of scholaricine-type MIAs. Preakuammicine then spontaneously lost HCHO to form (−)-akuammicine, which, through further redox reactions, yielded characteristic scholaricine-type MIAs such as scholaricine and 19-*epi*-scholaricine.

Due to the structural complexity and diversity of MIAs, few studies invested significant effort in constructing MIA virtual databases based on BPs. Building on the basic framework of subtype MIAs established in the BPs above, we created an exploratory biosynthetic pathway framework for MIAs from the *Alstonia* genus. We developed a corresponding virtual database, utilizing resources such as SciFinder, Mssbank, HMDB, Phytohub, and others, aiming to improve the annotation coverage of MIAs. Aside from reported authorized reference MIAs, the potential structures for each subtype MIA were empirically constructed solely based on differences in substituted groups and sites (Figure S62). This virtual database markedly expanded the potential scope of MIAs and increased confidence in their annotation. Using characteristic scholaricine-type MIAs in FBMN as an example (Figure 7), C-12 was often replaced by H, OH, OCH_3_, etc., C-16 was typically replaced by CHO, COOH, COOCH_3_, etc., C-19 undergone oxidation to form carbonyl, hydroxyl, and double bonds (at C-19 and C-20), and N-4 was oxidized. Consequently, more than 70 reported and potential scholaricine-type MIAs were empirically generated. Overall, 318 candidates of different-subtype MIAs, regardless of stereoconfiguration, were identified based on the BPs of *Alstonia* MIAs, serving as a vital reference for the structural annotation of MIAs in ALAS.

### 2.4. Systematic Annotation of MIAs in ALAS

The integrated MCEs/MS^2^-FBMN/BP workflow was used to comprehensively annotate the main MIAs in ALAS (Figure 8A). In brief, (1) the accurate mass and molecular formula of all nodes in FBMN were characterized based on MCEs/MS^2^ spectra; and (2) a comparative analysis of MS^2^ features and cosine similarity between reference nodes (or automatically identified nodes in GNPS Libraries) and unknown nodes was performed. The similarities of DPIs and CCs indicated information about the parent skeleton, while differences in NLs/RLs helped deduce substituent modifications on the basic structure; (3) finally, the structural candidates of MIAs based on the MPs were applied for efficient annotation of unknown nodes in FBMN. These candidates were further indexed in SciFinder to determine whether they had been reported in the *Alstonia* genus and to predict their structural novelty. Using this workflow, the identified MIAs were tentatively classified into four annotation levels: Level 1, confirmed by the reference standard; Level 2, speculated based on the library spectrum match or diagnostic fragmentation evidence; Level 3, tentative candidate classification; and Level 4, exact molecular formula.

The representative scholaricine-type MIAs served as examples to illustrate the structural annotation of the neighboring nodes. Nodes 7 and 10 were precisely identified as scholaricine and 19-*epi*-scholaricine by comparing RT and MS^2^ features with standard substances (Figure 8B). Node 66 (*m*/*z* 357.1809 [M+H]^+^, C_20_H_24_N_2_O_4_) shared identical MS^1^ and MS^2^ spectra with 19-*epi*-scholaricine (cosine 0.90) and exhibited consistent DPIs, *m*/*z* 325.15 [M+H-CH_4_O]^+^, *m*/*z* 307.14 [M+H-CH_4_O-H_2_O]^+^, *m*/*z* 253.10 [M+H-CH_4_O-C_4_H_8_O]^+^, *m*/*z* 225.10 [M+H-CH_4_O-C_4_H_8_O-CO]^+^, and *m*/*z* 210.05 [M+H-CH_4_O-C_4_H_8_O-C_2_H_5_N]^+^, indicating that both MIAs shared the same CCs and NLs/RLs. Subsequently, BP screening was performed to deduce that node 66 was a chiral isomer of 19-*epi*-scholaricine, tentatively assigned as a scholaricine isomer (Figure 8C). Node 19 (*m/z* 371.1967 [M+H]^+^, C_21_H_26_N_2_O_4_) showed an additional 14 Da compared to the precursor ion and fragment ions when compared with 19-*epi*-scholaricine (cosine 0.88). The DPIs of node 19 contained *m*/*z* 339.17 [M+H-CH_4_O]^+^, *m*/*z* 267.11 [M+H-CH_4_O-C_4_H_8_O]^+^, *m*/*z* 239.12 [M+H-CH_4_O-C_4_H_8_O-CO]^+^, and *m*/*z* 224.07 [M+H-CH_4_O-C_4_H_8_O-C_2_H_5_N]^+^, indicating it had consistent fragment patterns with 19-*epi*-scholaricine. Node 19 was believed to be a derivative of 19-*epi*-scholaricine (scholaricine), based on methylation at the C-12 hydroxyl, identified as 12-methoxy-echitamidine (Figure 8C). Nodes 33 and 45 (*m*/*z* 341.1858 [M+H]^+^, C_20_H_24_N_2_O_3_) showed consistent MS^1^ and MS^2^ spectra, meaning that they were isomers. For example, node 45 displayed a 15.99 Da (O) loss from the precursor and fragment ions when compared with 19-*epi*-scholaricine (cosine 0.90). Its DPIs contained *m*/*z* 309.16 [M+H-CH_4_O]^+^, *m*/*z* 291.15 [M+H-CH_4_O-H_2_O]^+^, *m*/*z* 237.10 [M+H-CH_4_O-C_4_H_8_O]^+^, *m*/*z* 209.11 [M+H-CH_4_O-C_4_H_8_O-CO]^+^, and *m*/*z* 194.06 [M+H-CH_4_O-C_4_H_8_O-C_2_H_5_N]^+^, suggesting that it shared the same CCs and NLs/RLs with 19-*epi*-scholaricine. Nodes 45 and 33 were deduced to be derivatives of 19-*epi*-scholaricine (scholaricine), produced by deoxygenation at C-12, and were identified as echitamidine and its isomer (Figure 8C).

The nodes directly connected to authorized reference MIAs (nodes) that showed high similarities of MS^2^ features were easily annotated by comparing DPIs, CCs, and NLs/RLs. The nodes indirectly associated with authorized reference MIAs (nodes) were first analyzed through fragment ions to identify potential DPIs for classifying the MIA subtypes, then referenced to BPs to preliminarily predict possible structures. For single nodes in FBMN, a SciFinder search revealed that the molecules were not reported in the *Alstonia* genus and exhibited significant variation in fragment patterns compared to MIA standards, suggesting potential novelty in their structures. Using the proposed MCEs/MS^2^-FBMN/BP workflow, the chemical profile of MIAs in ALAS was clearly illustrated, and 229 MIAs were interactively annotated and classified for the first time (Table S3).

Our study offered a thorough analysis of the MIA profiles in ALAS. By examining the subtypes of MIAs, we found that scholaricine-type MIAs showed the highest peak intensity, making up 23.22% of the total peak area, followed by picrinine-type MIAs at 20.59% and vallesamine-type MIAs at 19.17% (Table S3). This finding supported our earlier conclusion that scholaricine, 19-*epi*-scholaricine, picrinine, and vallesamine were the main bioactive markers in ALAS [14,15,16,17,18,19,20]. While our analytical framework greatly increased MIA annotation coverage, structural elucidation was limited to plausible planar structure assignments due to the inherent limits of mass spectrometry in distinguishing stereoisomers. Without standard substances, the stereoconfiguration of annotated MIAs cannot be fully confirmed based on LC-HRMS. Notably, the proposed structures of unknown compounds showed great potential for novelty due to substantial differences in fragment patterns from the known MIAs. These findings offered strategic guidance for future bioactivity-guided isolation studies to discover MIAs with new scaffolds and improved bioactivities.

## 3. Materials and Methods

### 3.1. Chemicals and Reagents

**Figure 2:**
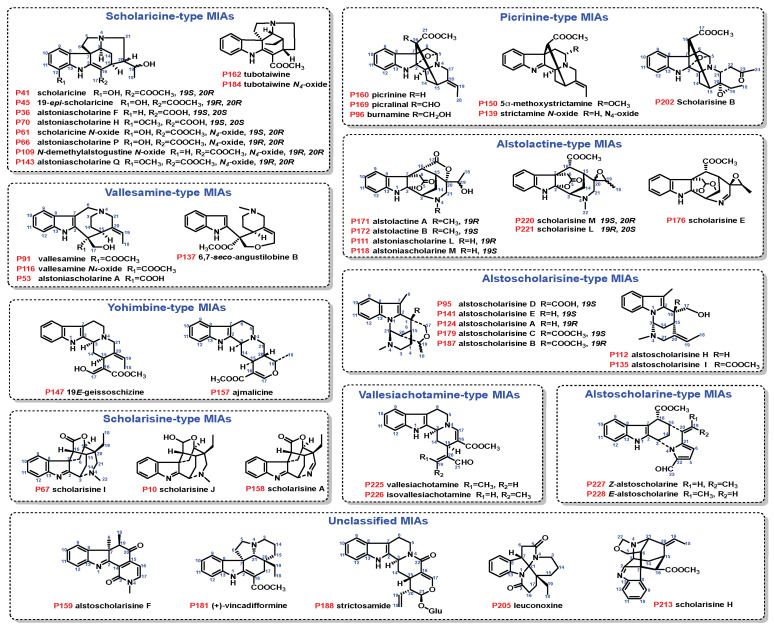
Structures of 48 authorized reference MIAs used in this study.

**Figure 3:**
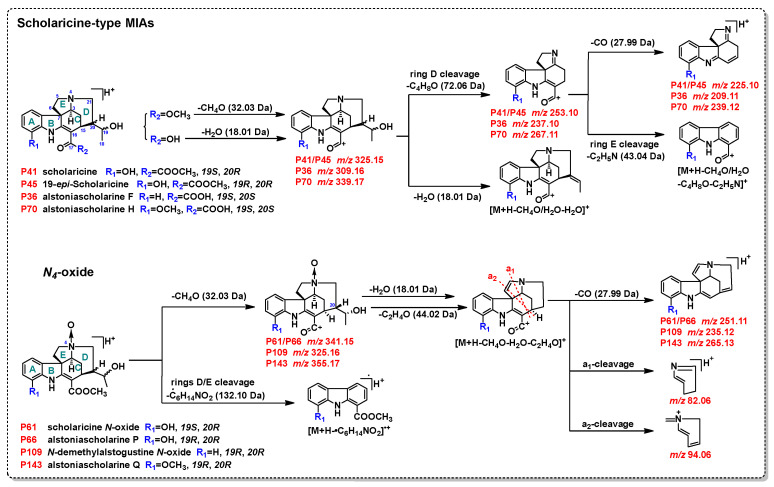
Characteristic fragment patterns of scholaricine-type MIAs.

**Figure 4:**
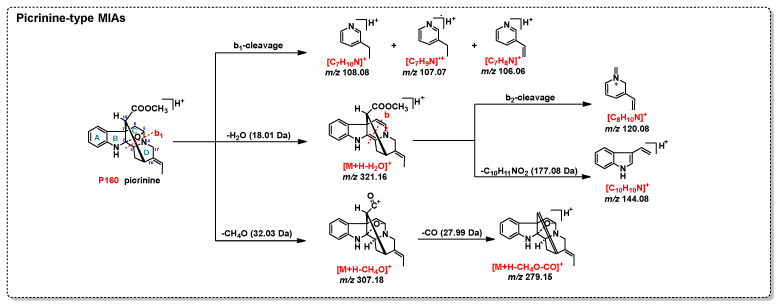
Characteristic fragment patterns of picrinine-type MIAs.

**Figure 5:**
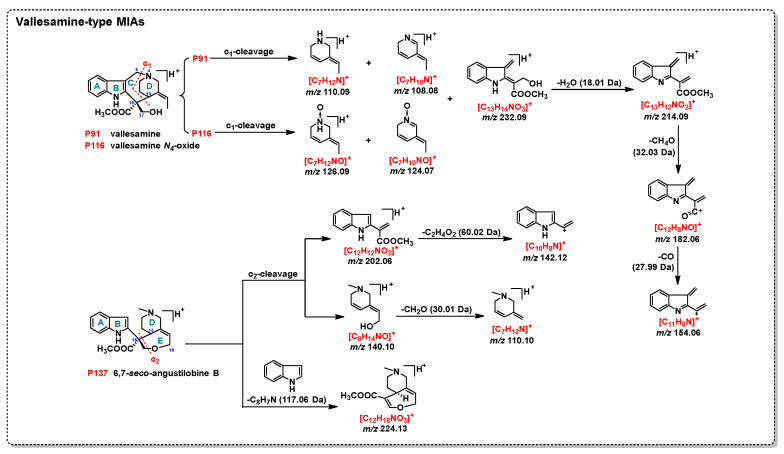
Characteristic fragment patterns of vallesamine-type MIAs.

**Figure 6:**
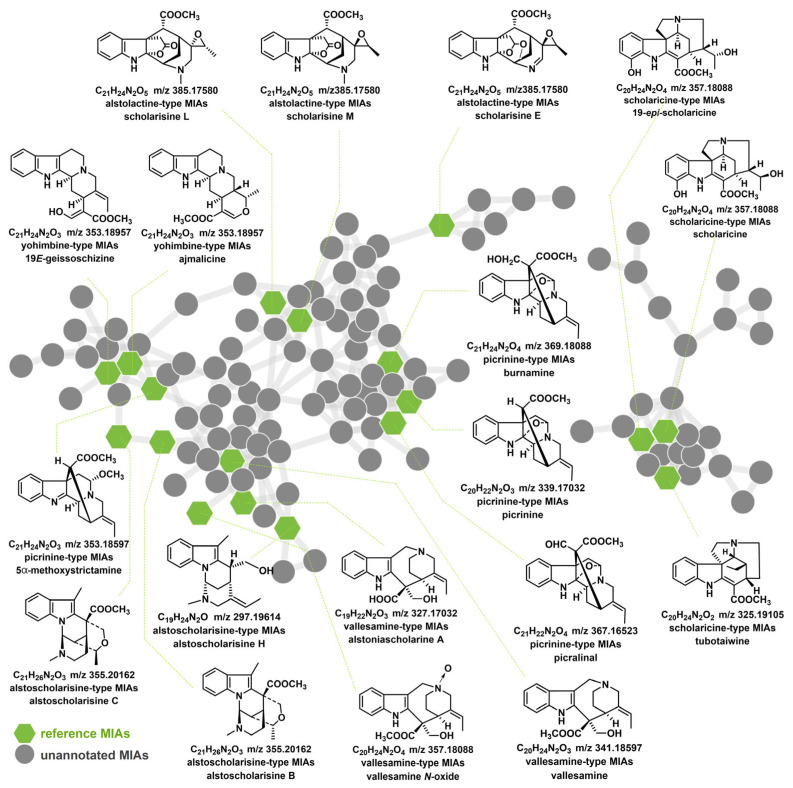
In-house annotation of authorized reference MIAs in ALAS’s major clusters of feature-based molecular networking (FBMN).

**Figure 7:**
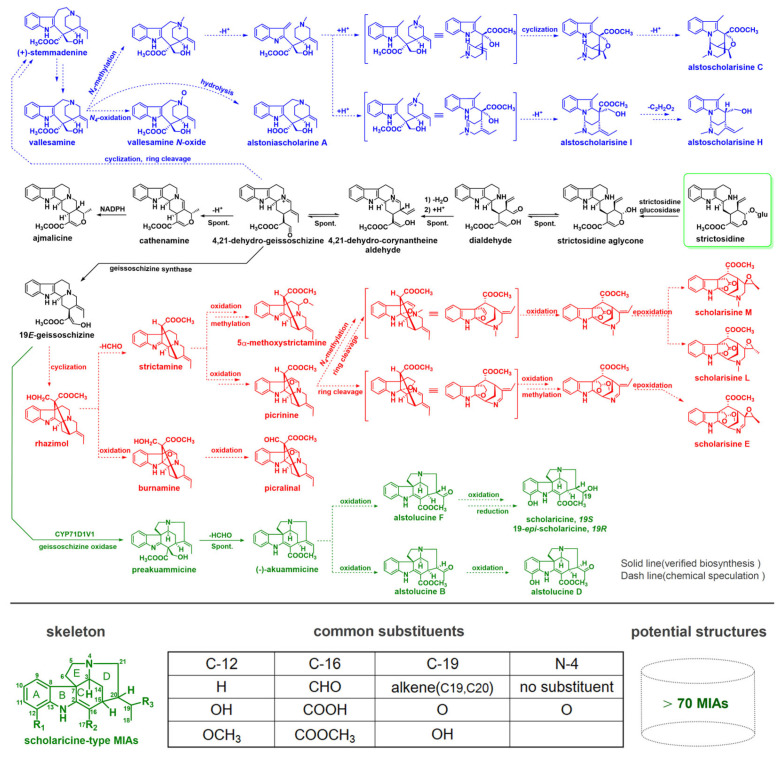
Typical biosynthetic pathways of MIAs in the *Alstonia* genus; the potential structures of scholaricine-type MIAs based on the fundamental skeleton and typical substituents.

**Figure 8:**
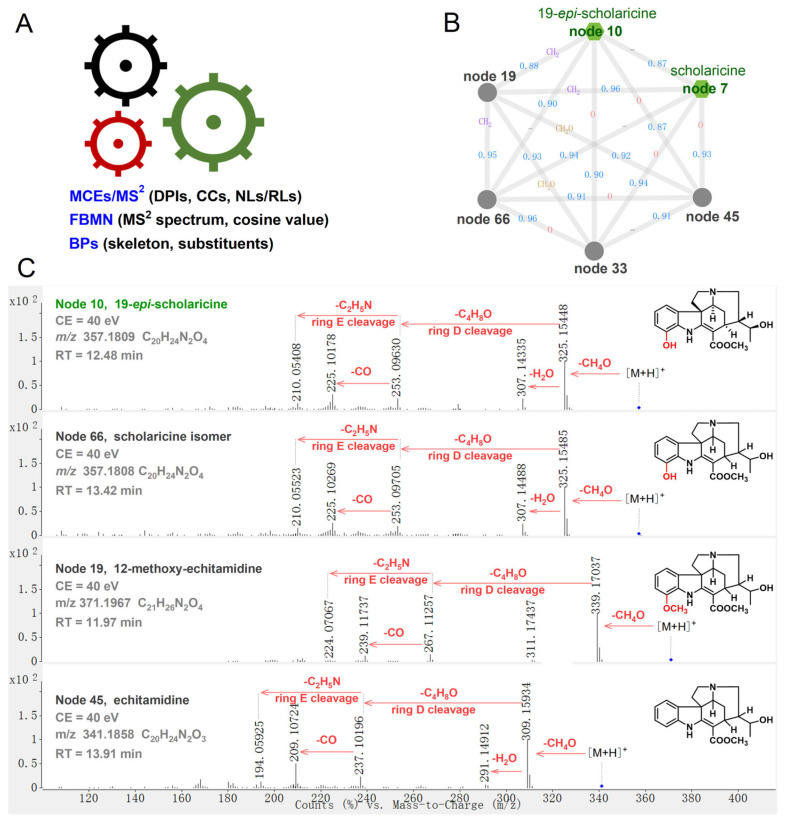
Comprehensive annotation of MIAs in ALAS (**A**); an example (scholaricine-type MIAs) to illustrate this workflow (**B**,**C**).

For LC-HRMS analysis, acetonitrile, ethanol, methanol, and formic acid were purchased from Merck KGaA Co., Ltd. (Darmstadt, Germany). Hydrochloric acid, analytical ethanol, and other reagents were obtained from Sinopharm Chemical Reagent Co., Ltd. (Shanghai, China). Distilled water was sourced from Watson’s Specialty Store (Hong Kong). Forty-eight authorized reference MIAs were isolated from *A. scholaris* by Luo’s group and stored in a refrigerator at −20 °C. According to published literature, the absolute configuration of MIAs was identified using NMR, HRMS, X-ray, etc. (Table S1). The MIAs (Figure 2) included scholaricine-type (scholaricine, 19-*epi*-scholaricine, alstoniascholarine F, alstoniascholarine H, scholaricine N-oxide, alstoniascholarine P, N-demethylalstogustine N-oxide, alstoniascholarine Q, tubotaiwine, and tubotaiwine N-oxide); picrinine-type (picrinine, picralinal, burnamine, 5*α*-methoxystrictamine, strictamine N-oxide, and scholarisine B); vallesamine-type (vallesamine, vallesamine N-oxide, alstoniascholarine A, and 6,7-seco-angustilobine B); alstolactine-type (scholarisine M, scholarisine L, scholarisine E, alstolactine A, alstolactine B, alstoniascholarine L, and alstoniascholarine M); yohimbine-type (19*E*-geissoschizine abd ajmalicine); alstoscholarisine-type (alstoscholarisine D, alstoscholarisine E, alstoscholarisine A, alstoscholarisine C, alstoscholarisine B, alstoscholarisine H, and alstoscholarisine I); scholarisine-type (scholarisine I, scholarisine J, and scholarisine A); vallesiachotamine-type (vallesiachotamine and isovallesiachotamine); alstoscholarine-type (*Z*-alstoscholarine and *E*-alstoscholarine); and unclassified MIAs (strictosamide, (+)-vincadifformine, leuconoxine, and scholarisine H).

### 3.2. ALAS Preparation

*A. scholaris* was collected in June 2013 in Pu’er city (Yunnan Province, China) and identified by Dr. Xiaodong Luo, Kunming Institute of Botany, Chinese Academy of Sciences (Kunming, China). The Chinese Academy of Sciences’ State Key Laboratory of Phytochemistry and Plant Resources in West China is home to the voucher specimen (Luo20130601). The dried *A. scholaris* leaf powder was extracted using 90% ethanol under reflux conditions (3 × 4), and the solvent was evaporated in a vacuum to obtain the ethanolic extract. The ethanolic extract was dissolved in 0.3% aqueous hydrochloric acid solution, and the residue was identified as a non-alkaloid fraction. Subsequently, the acidic solution was adjusted to pH 9–10 with 10% aqueous ammonia and extracted with EtOAc to yield ALAS (containing main MIAs) [14,19]. ALAS were dissolved in methanol to 100 μg/mL and then filtered through a 0.22 μm organic membrane for LC-HRMS analysis. Each sample was prepared three times in parallel. The reliability and stability of the instrumental analysis were confirmed through quality control (QC) samples, which were prepared by mixing equal amounts of the tested samples.

### 3.3. LC-HRMS Conditions

A 1290 Infinity II ultrahigh-performance liquid chromatography (UHPLC) system coupled with a 6545B quadrupole time-of-flight mass spectrometry system, equipped with a dual AJS electrospray ionization (ESI) source (Agilent Technologies, Santa Clara, CA, USA), was used for LC-HRMS analysis. Chromatographic separation was carried out on an SB C18 column (2.1 mm × 100 mm, 1.8 μm, Agilent Technologies) maintained at a constant temperature of 35 °C. The solvent system consisted of phases A (water with 0.1% formic acid) and B (acetonitrile with 0.1% formic acid), with a gradient elution at a flow rate of 0.15 mL/min, as follows: 0–7 min, 4–12% B; 7–12 min, 12–17% B; 12–16 min, 17% B; 16–23 min, 17–30% B; 23–28 min, 30–45% B; 28–35 min, 45–65% B; 35–40 min, 65–98% B; and 40–48 min, 98% B. The injected volume was 0.5 μL.

Mass spectra conditions in positive ESI mode for accurate compound characterization were optimized as follows: gas temperature 325 °C, 9 L/min; nebulizer pressure 35 psi; sheath gas temperature 365 °C, 11 L/min; nozzle voltage 1.0 kV; VCAP voltage 4.0 kV; OCT1 RF Vpp 750 V; skimmer voltage 65 V; and fragmentor 150 V. MS^1^ data were collected from 100 to 1000 Da, and MS^2^ data were acquired from 25 to 1000 Da. Collision energies (CEs) from low to high values (10–40 eV) were optimized to accurately capture MS^2^ spectra of individual compound features, based on our previous studies [27,28,29].

### 3.4. Construction of In-House and Online HRMS Database of Authorized Reference MIAs

Each MIA was properly dissolved in methanol and then filtered through a 0.22 μm organic membrane for LC-HRMS analysis. The MCEs/MS^2^ strategy was initially implemented using Agilent MassHunter Qualitative Analysis Navigator (B.08.00) to build an in-house MS^2^ database of 48 reference MIAs at 10, 20, and 40 eV, respectively (Figures S2–S49). Meanwhile, the raw data in the “.d” format were converted to “.MGF” files and then uploaded to the online GNPS libraries. Each MIA’s MS^2^ spectrum received a unique GNPS spectrum ID (Figures S2–S49), allowing researchers to access these spectra for free via the website https://gnps-library.ucsd.edu/ (accessed on 19 April 2022).

### 3.5. Construction of Feature-Based Molecular Networking of MIAs in ALAS

The MSconvert tool was used to convert the original .d data to the .mzML format, which was then submitted to MZmine 2 following the previous processing flow [13,21]. The present study improved the processing of LC-MS^2^ datasets of MIAs in ALAS. The main steps are shown in Section 3.2, and the “.csv” and “.mgf” files were exported to GNPS for FBMN analysis [9]. FBMN parameters were configured as follows: The data was filtered to remove all MS^2^ fragment ions within ±17 Da of the precursor *m*/*z*. MS^2^ spectra were window-filtered by selecting only the top 6 fragment ions within the ±50 Da window across the spectrum. The precursor ion mass tolerance was set to 0.02 Da, and the MS^2^ fragment ion tolerance to 0.05 Da. Edges between two nodes were retained in the network if each node was among the other’s top 10 most similar nodes. Finally, the maximum size of a molecular family was set to 100, and the lowest-scoring edges were removed from the molecular families until the size threshold was met. The spectra in the network were then searched against GNPS spectral libraries [30,31]. The library spectra were filtered in the same way as the input data. All matches between network spectra and library spectra had to have a score above 0.6 and at least six matched peaks. FBMNs were then generated using optimized values of the “Minimum Cosine Score” (a similarity score between two MS^2^ spectra that allows linking them) at 0.70, 0.58, 0.50, and 0.40, respectively. The FBMN jobs can be accessed via the GNPS website, as shown in the Supplementary Materials. The molecular networks were visualized using Cytoscape software (v3.9) [32]. The nodes indicated the precursor ions, and cosine values showed the similarity between nodes [23].

### 3.6. Analysis of Potential MIAs of the Alstonia Genus Based on Biosynthetic Pathways

The *Alstonia* genus mainly includes *A. scholaris*, *Alstonia macrophylla*, *Alstonia yunnanensis*, *Alstonia henryi*, *Alstonia mairei*, and *Alstonia rupestris*. *A. scholaris* is one of the representative plant species. The basic framework (individual subtype-MIAs) was first built based on the classical BPs of *Alstonia* MIAs according to the Kyoto Encyclopedia of Genes and Genomes (KEGG) and the existing literature. Additionally, multiple databases such as SciFinder, MsSID, HMDB, and Phytohub were consulted under the *Alstonia* genus. Then, each MIA subtype’s typical substituents and substitution sites were systematically analyzed.

## 4. Conclusions

The *Alstonia* genus has attracted increasing interest due to the distinctive chemical structures of MIAs and the diverse biological activities. In this study, a combined MCEs/MS^2^-FBMN/BPs workflow was developed using a UHPLC-ESI-QTOF-MS^E^ platform, greatly improving the efficiency and accuracy of alkaloid component analysis. The fragmentation patterns of various structural skeletons (ten subtypes out of 48 reference MIAs), including scholaricine-type, picrinine-type, vallesamine-type, alstolactine-type, yohimbine-type, alstoscholarisine-type, scholarisine-type, vallesiachotamine-type, alstoscholarine-type, and five unclassified MIAs, were systematically examined. The fragmentation behaviors observed through MCEs/MS^2^ were integrated with FBMN/BPs to enable detailed characterization of the enriched MIAs. A total of 229 MIAs were identified and classified in ALAS (*A. scholaris*) for the first time, including 30 previously unreported compounds. Overall, this study provides an effective workflow for identifying MIAs in complex herbal medicines. Notably, these newly discovered MIAs could serve as potential candidates for further MS-guided isolation and drug discovery investigations.

## Figures and Tables

**Figure 1:**
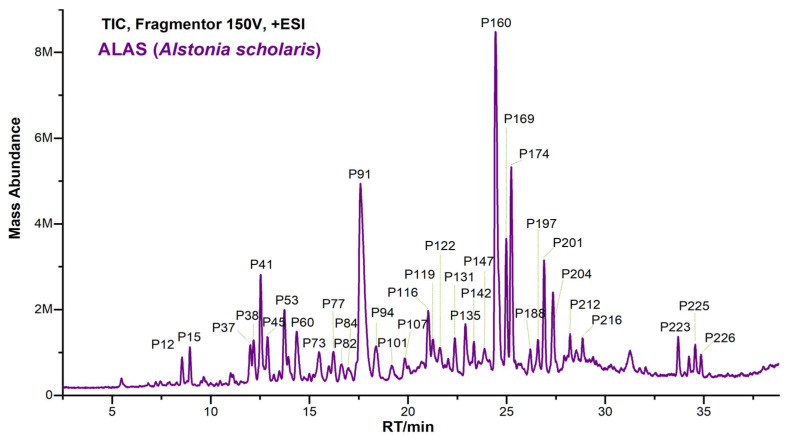
Characteristic total ion chromatogram of ALAS (*Alstonia scholaris*).

## Data Availability

Data are contained within the article.

## Supplementary Materials

The following supporting information can be downloaded at: https://www.mdpi.com/article/10.3390/plants14142177/s1.

## Abbreviations

The following abbreviations are used in this manuscript:

MIAs	Monoterpene indole alkaloids
LC-HRMS	Liquid chromatography coupled with high-resolution mass spectrometry
UHPLC-ESI-QTOF-MS^E^	Ultrahigh-performance liquid chromatography electrospray ionization quadrupole time-of-flight mass spectrometry
MCEs/MS^2^	MS^2^ spectra under multiple collision energies
DPIs	Diagnostic product ions
CCs	Characteristic cleavages
NLs	Neutral losses
RLs	Radical losses
FBMN	Feature-based molecular networking
BPs	Biosynthetic pathways
ALAS	Alkaloids from the leaves of *Alstonia scholaris*
