# Supplementary material for: LC-HRMS Coupling to Feature-Based Molecular Networking to Efficiently Annotate Monoterpene Indole Alkaloids of Alstonia scholaris
Source: Plants (Basel). 2025 Jul 14;14(14):2177. doi: 10.3390/plants14142177 (PMC12298960; doi:10.3390/plants14142177)
Supplement: Supplementary file 1 [file plants-14-02177-s001.zip › plants-3667385-supplementary.pdf]

Supporting Information

LC-HRMS Coupling to Feature-Based Molecular Networking to Efficiently Annotate Monoterpene Indole Alkaloids of *Alstonia scholaris*

Ying-Jie He [1,2,3,†], Yan Qin [1,†], and Xiao-Dong Luo [1,4,*]

1 Key Laboratory of Medicinal Chemistry for Natural Resource, Ministry of Education and Yunnan Province, Yunnan Characteristic Plant Extraction Laboratory, School of Chemical Science and Technology, Yunnan University, Kunming, 650500, China

2 School of Public Health, Kunming Medical University, Kunming 650500, China

3 School of Environmental Science and Engineering, Southern University of Science and Technology, Shenzhen 518055, China

4 State Key Laboratory of Phytochemistry and Plant Resources in West China, Kunming Institute of Botany, Chinese Academy of Sciences, Kunming 650201, China

* Correspondence: xdluo@ynu.edu.cn

† Ying-Jie He and Yan Qin contributed equally to this work.

ORCID:
Ying-Jie He: orcid.org/0000-0001-8361-2528
Yan Qin: orcid.org/0000-0003-4371-3548
Xiao-Dong Luo: orcid.org/0000-0002-6768-5679

Table of Contents

Workflow for structural annotation of MIAs 3

General information of 48 reference MIAs 4

In-house MCEs/MS^2 spectra of 48 reference MIAs (CEs = 10, 20, 40 eV) 6

Characteristic fragmentation patterns of the subtype of MIAs 30

Common neutral losses and radical losses of MIAs 39

MZmine 2 processing of LC-MS2 datasets 40

FBMN construction of ALAS and automatic annotation of MIAs 41

Hypothetical biogenetic pathways of *Alstonia* MIAs 44

Construction of in silico database of *Alstonia* MIAs 45

Systematically annotated MIAs in ALAS by MCEs/MS^2-FBMN/BPs 46

References 64

Workflow for structural annotation of MIAs

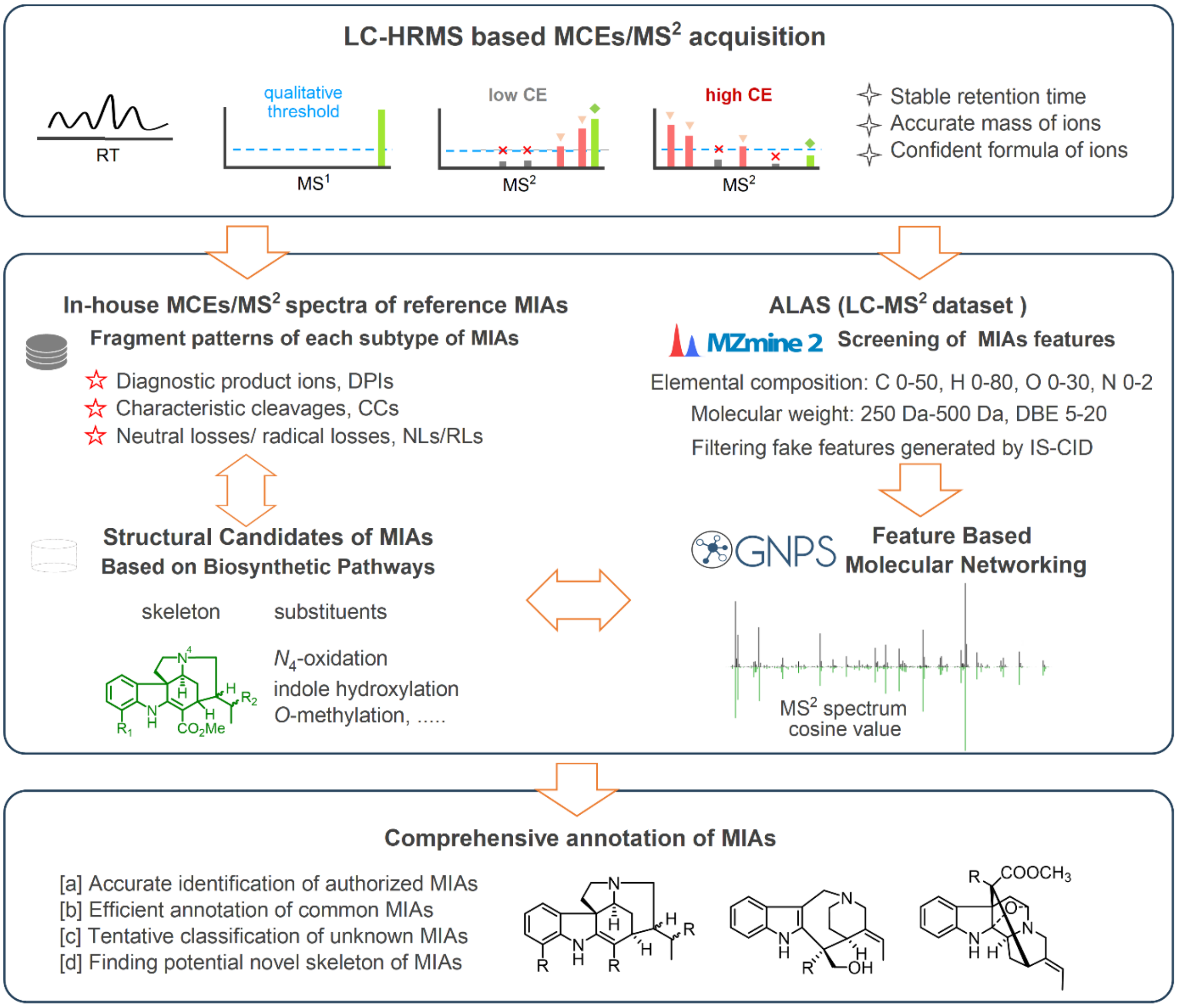

Figure S1. Workflow for structural annotation of MIAs in ALAS based on UHPLC-ESI-QTOF-MS^E.

General information of 48 reference MIAs

Table S1. General information of 48 reference MIAs used in this study.

Peak No.	Reference MIAs	CAS	Formula	Molecular weight	Ref.*
			**Scholaricine-type MIAs**		
**P41**	scholaricine	99694-90-3	$C_{20}H_{24}N_2O_4$	356.17361	[1], [2]▲
**P45**	19-*epi*-scholaricine	132923-06-9	$C_{20}H_{24}N_2O_4$	356.17361	[3], [4]▲
**P36**	alstoniascholarine F	1792216-83-1	$C_{19}H_{22}N_2O_3$	326.16304	[5]
**P70**	alstoniascholarine H	1792216-85-3	$C_{20}H_{24}N_2O_4$	356.17361	[5]
**P61**	scholaricine *N*-oxide	2134621-86-4	$C_{20}H_{24}N_2O_5$	372.16852	[6]
**P66**	alstoniascholarine P	2023815-29-2	$C_{20}H_{24}N_2O_5$	372.16852	[3]
**P109**	*N*-demethylalstogustine *N*-oxide	125205-50-7	$C_{20}H_{24}N_2O_4$	356.17361	[3], [7]▲
**P143**	alstoniascholarine Q	2023815-35-0	$C_{21}H_{26}N_2O_5$	386.18417	[3]
**P162**	tubotaiwine	6711-69-9	$C_{20}H_{24}N_2O_2$	324.18378	[8], [9]▲
**P184**	tubotaiwine *N*-oxide	40169-69-5	$C_{20}H_{24}N_2O_3$	340.17869	[8], [9]▲
			**Picrinine-type MIAs**		
**P160**	picrinine	4684-32-6	$C_{20}H_{22}N_2O_3$	338.16304	[10], [11]▲
**P169**	picralinal	20045-06-1	$C_{21}H_{22}N_2O_4$	366.15796	[12]
**P96**	burnamine	6808-68-0	$C_{21}H_{24}N_2O_4$	368.17361	[3], [13]▲
**P150**	5α-methoxystrictamine	870995-64-5	$C_{21}H_{24}N_2O_3$	352.17869	[10], [14]▲
**P139**	strictamine *N*-oxide	90850-95-6	$C_{20}H_{22}N_2O_3$	338.16304	[1], [15]▲
**P202**	scholarisine B	1236196-02-3	$C_{23}H_{26}N_2O_5$	410.18417	[16]
			**Vallesamine-type MIAs**		
**P91**	vallesamine	3368-87-4	$C_{20}H_{24}N_2O_3$	340.17869	[1], [4]▲
**P116**	vallesamine *N*-oxide	126594-73-8	$C_{20}H_{24}N_2O_4$	356.17361	[1], [4]▲
**P53**	alstoniascholarine A	1792216-74-0	$C_{19}H_{22}N_2O_3$	326.16304	[5]
**P137**	6,7-*seco*-angustilobine B	112464-23-0	$C_{20}H_{24}N_2O_3$	340.17869	[17], [18],[19]▲
			**Alstolactine-type MIAs**		
**P220**	scholarisine M	1636144-92-7	$C_{21}H_{24}N_2O_5$	384.16852	[10]
**P221**	scholarisine L	1636144-91-6	$C_{21}H_{24}N_2O_5$	384.16852	[10]
**P176**	scholarisine E	1236196-08-9	$C_{21}H_{24}N_2O_5$	384.16852	[16]
**P171**	alstolactine A	1620959-24-1	$C_{20}H_{22}N_2O_5$	370.15287	[20]
**P172**	alstolactine B	1620848-15-8	$C_{20}H_{22}N_2O_5$	370.15287	[20]
**P111**	alstoniascholarine L	2023815-26-9	$C_{19}H_{20}N_2O_5$	356.13722	[3]

Peak No.	Reference MIAs	CAS	Formula	Molecular weight	Ref.*
**P118**	alstoniascholarine M	2023815-27-0	$C_{19}H_{20}N_2O_5$	356.13722	[3]
		**Yohimbine-type MIAs**			
**P147**	19*E*-geissoschizine	25920-79-0	$C_{21}H_{24}N_2O_3$	352.17869	[21]
**P157**	ajmalicine	483-04-5	$C_{21}H_{24}N_2O_3$	352.17869	[22], [23]▲
		**Alstoscholarisine-type MIAs**			
**P95**	alstoscholarisine D	1636132-03-0	$C_{20}H_{24}N_2O_3$	340.17869	[24]
**P141**	alstoscholarisine E	1636132-04-1	$C_{19}H_{24}N_2O$	296.18886	[24]
**P124**	alstoscholarisine A	1636132-00-7	$C_{19}H_{24}N_2O$	296.18886	[24]
**P179**	alstoscholarisine C	1636132-02-9	$C_{21}H_{26}N_2O_3$	354.19434	[24]
**P187**	alstoscholarisine B	1636132-01-8	$C_{21}H_{26}N_2O_3$	354.19434	[24]
**P112**	alstoscholarisine H	1862248-98-3	$C_{19}H_{24}N_2O$	296.18886	[25]
**P135**	alstoscholarisine I	1862248-99-4	$C_{21}H_{26}N_2O_3$	354.19434	[25]
		**Scholarisine-type MIAs**			
**P67**	scholarisine I	1632317-41-9	$C_{20}H_{22}N_2O_2$	322.16813	[10]
**P10**	scholarisine J	1636144-90-5	$C_{20}H_{24}N_2O_2$	324.18378	[10]
**P158**	scholarisine A	1002321-69-8	$C_{19}H_{18}N_2O_2$	306.13683	[26]
		**Vallesiachotamine-type MIAs**			
**P225**	vallesiachotamine	5523-37-5	$C_{21}H_{22}N_2O_3$	350.16304	[1], [27]▲
**P226**	isovallesiachotamine	34384-71-9	$C_{21}H_{22}N_2O_3$	350.16304	[1], [27]▲
		**Alstoscholarine-type MIAs**			
**P227**	*Z*-alstoscholarine	937245-48-2	$C_{22}H_{20}N_2O_3$	360.14739	[21]
**P228**	*E*-alstoscholarine	937245-47-1	$C_{22}H_{20}N_2O_3$	360.14739	[21]
		**Unclassified alkaloids**			
**P188**	strictosamide	23141-25-5	$C_{26}H_{30}N_2O_8$	498.20022	[22], [28]▲
**P159**	alstoscholarisine F	—	$C_{18}H_{16}N_2O_2$	292.12118	[29]
**P181**	(+)-vincadifformine	15539-10-3	$C_{21}H_{26}N_2O_2$	338.19943	[8], [30]▲
**P205**	leuconoxine	155416-24-3	$C_{19}H_{22}N_2O_2$	310.16813	[16], [31]▲
**P213**	scholarisine H	1636144-89-2	$C_{21}H_{22}N_2O_3$	350.16304	[10]

* 48 authorized substances were isolated from *A. scholaris* and their absolute configuration has been identified by NMR, HRMS, X-RAY, etc., according to our previously systematic studies.

▲ Compound was isolated firstly in the literature.

In-house MCEs/MS^2 spectra of 48 reference MIAs (CEs = 10, 20, 40 eV)

Shcolaricine-type MIAs

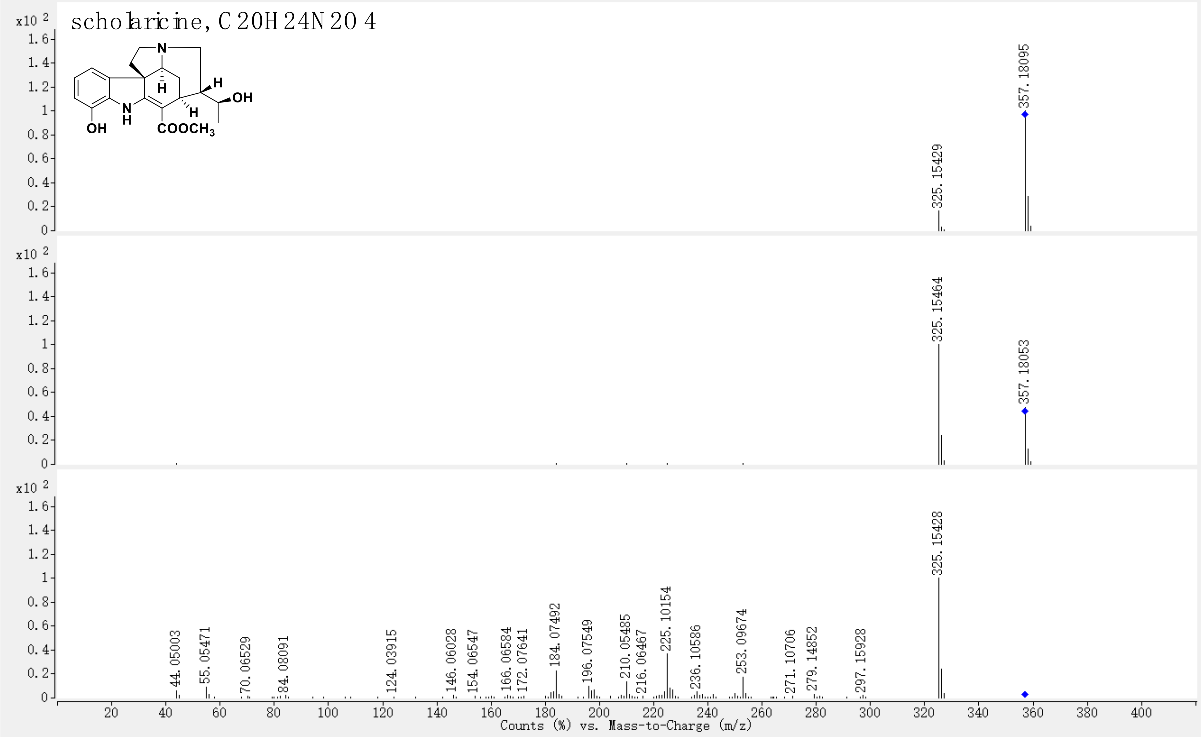

Figure S2. MCEs/MS^2 spectra of scholaricine (**P41**).
GNPS spectrum ID: CCMSLIB00006709963

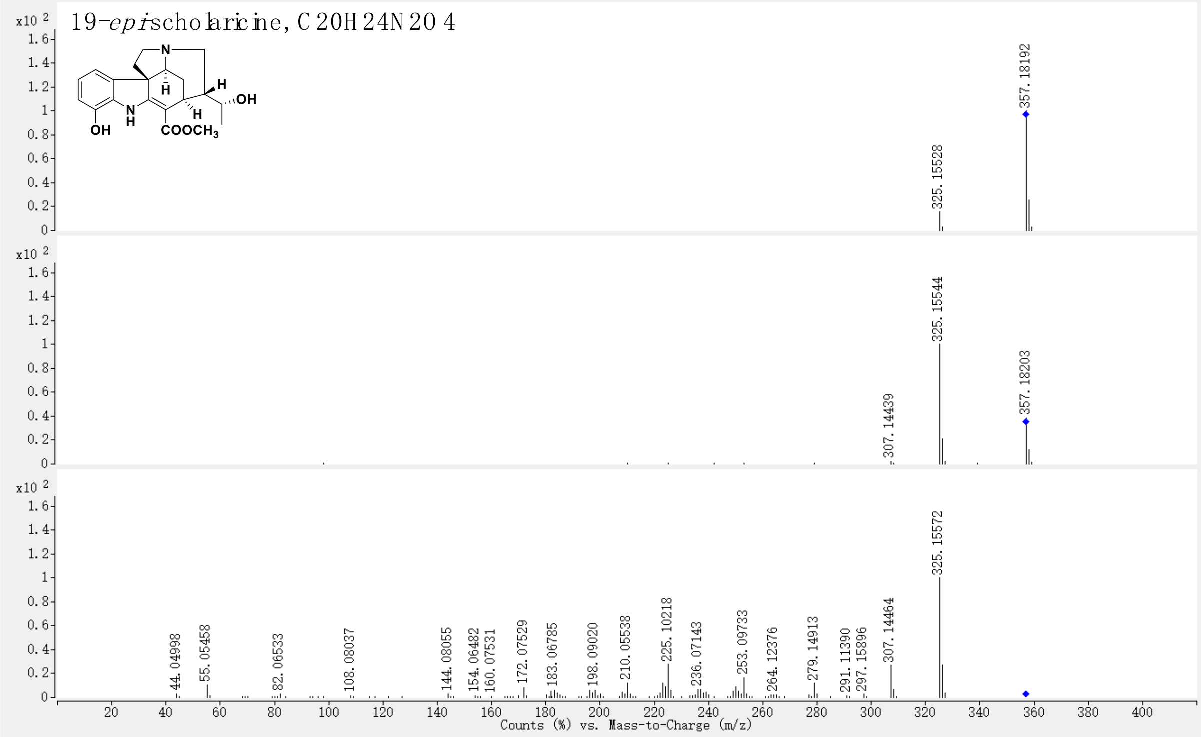

Figure S3. MCEs/MS^2 spectra of 19-*epi*-scholaricine (**P45**).
GNPS spectrum ID: CCMSLIB00006709966

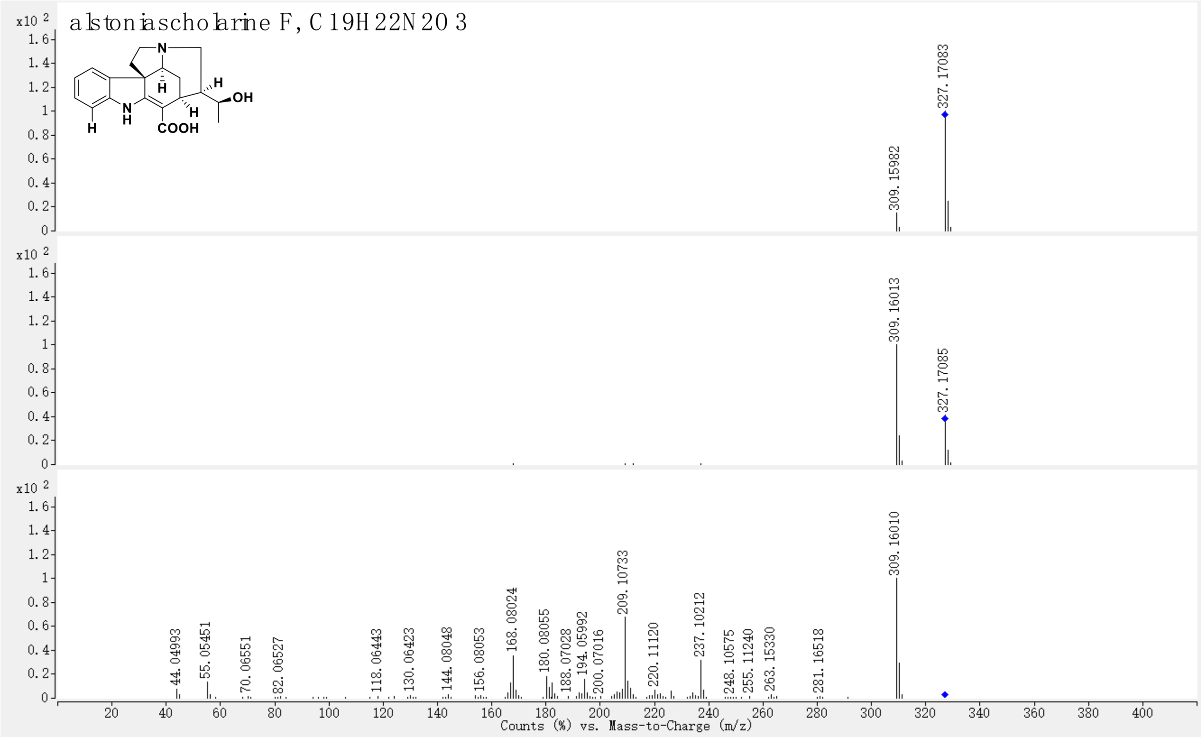

Figure S4. MCEs/MS^2 spectra of alstoniascholarine F (**P36**).
GNPS spectrum ID: CCMSLIB00006709967

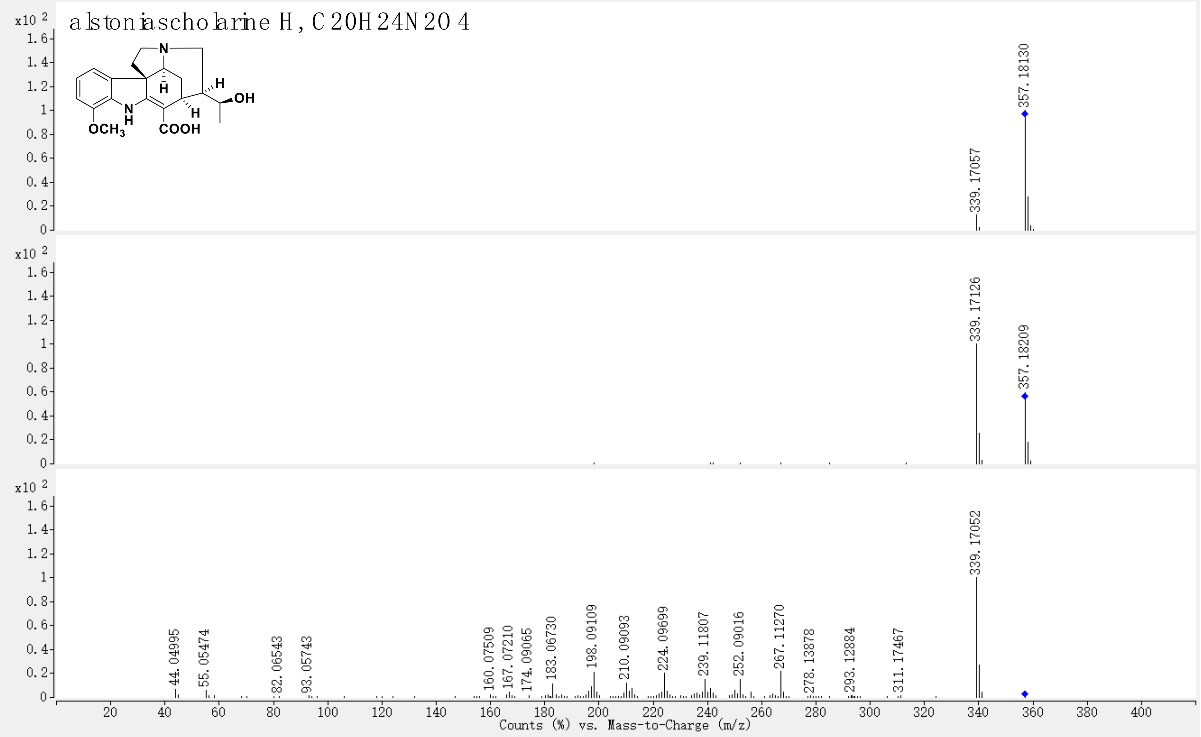

Figure S5. MCEs/MS^2 spectra of alstoniascholarine H (**P70**).
GNPS spectrum ID: CCMSLIB00006709968

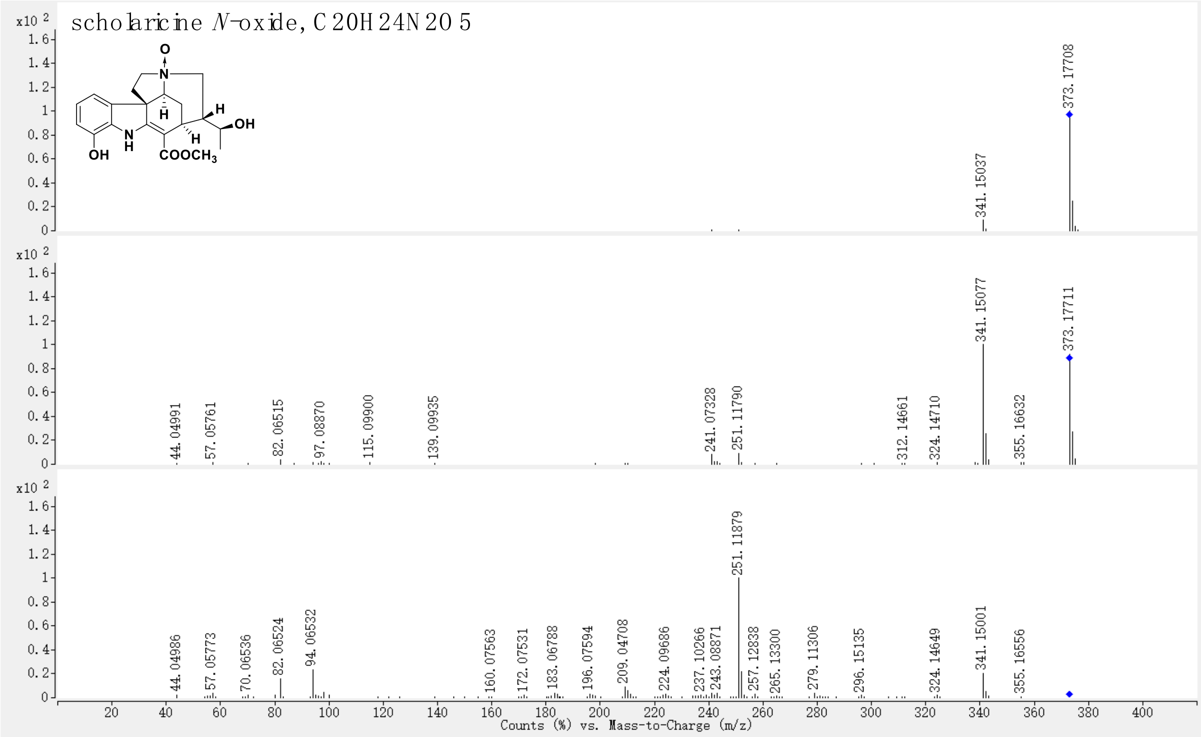

Figure S6. MCEs/MS² spectra of scholaricine *N*-oxide (**P61**).
GNPS spectrum ID: CCMSLIB00006709972

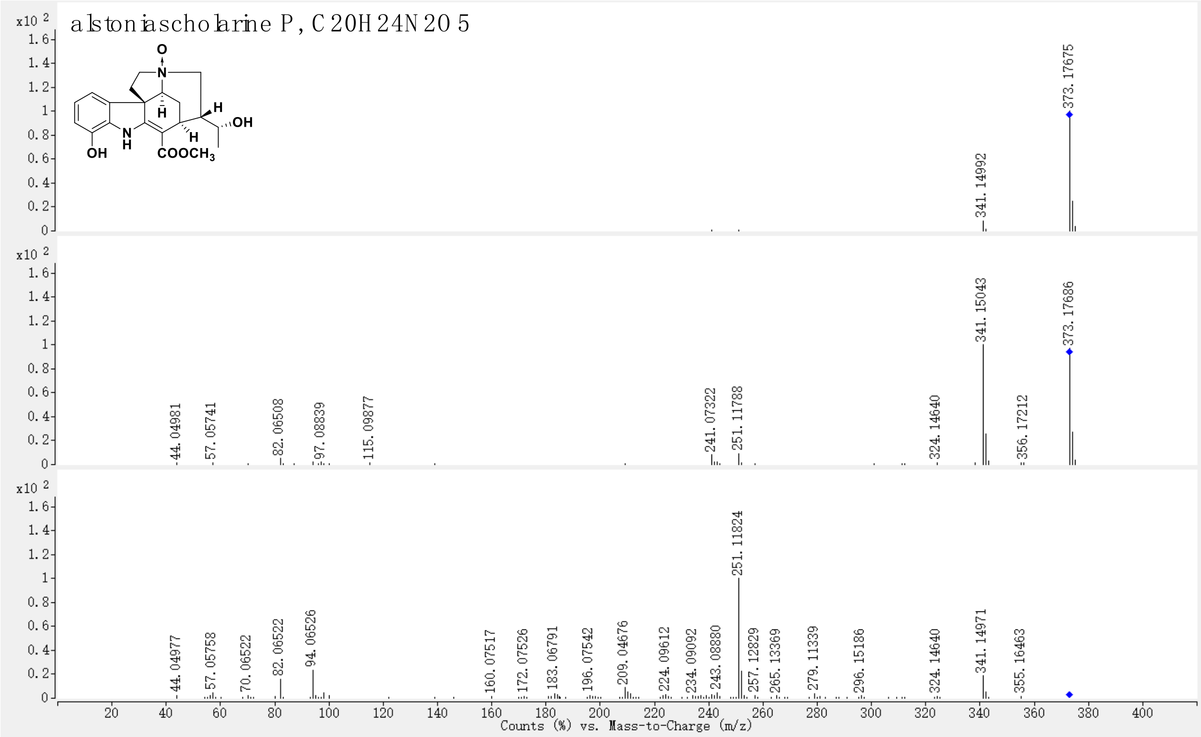

Figure S7. MCEs/MS² spectra of alstoniascholarine P (**P66**).
GNPS spectrum ID: CCMSLIB00006709970

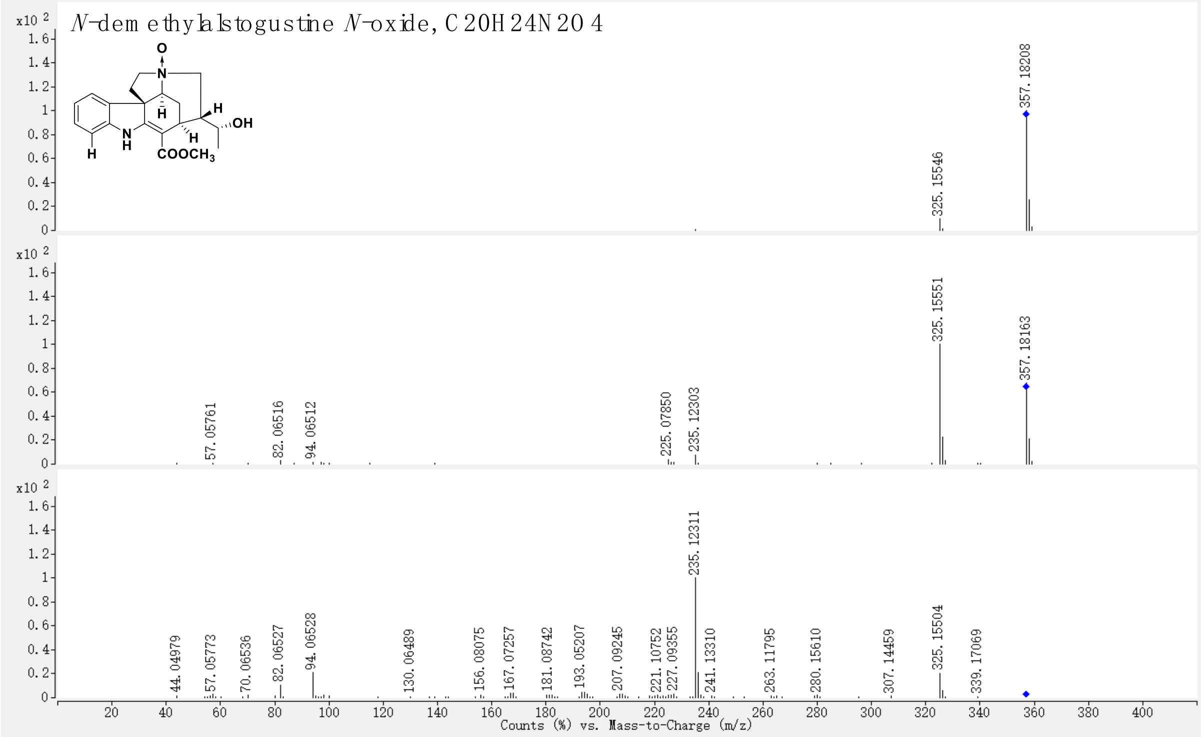

Figure S8. MCEs/MS^2 spectra of *N*-demethylalstogustine *N*-oxide (**P109**).
GNPS spectrum ID: CCMSLIB00006709971

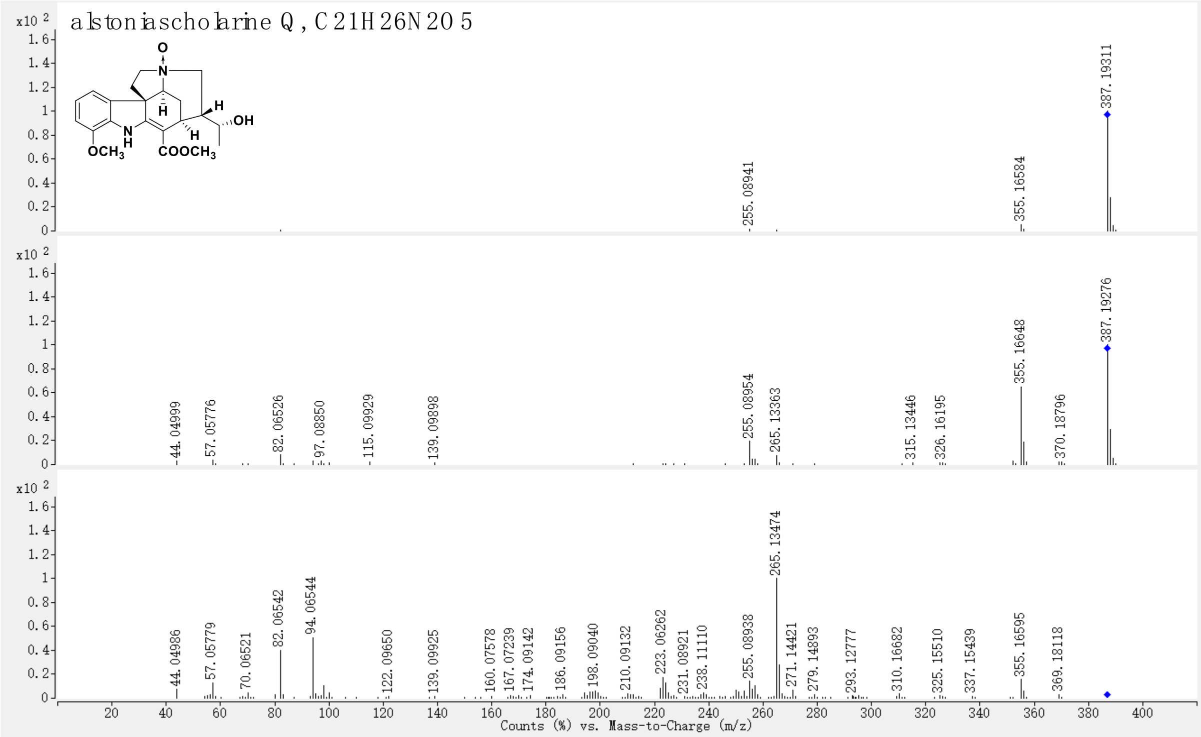

Figure S9. MCEs/MS^2 spectra of alstoniascholarine Q (**P143**).
GNPS spectrum ID: CCMSLIB00006709969

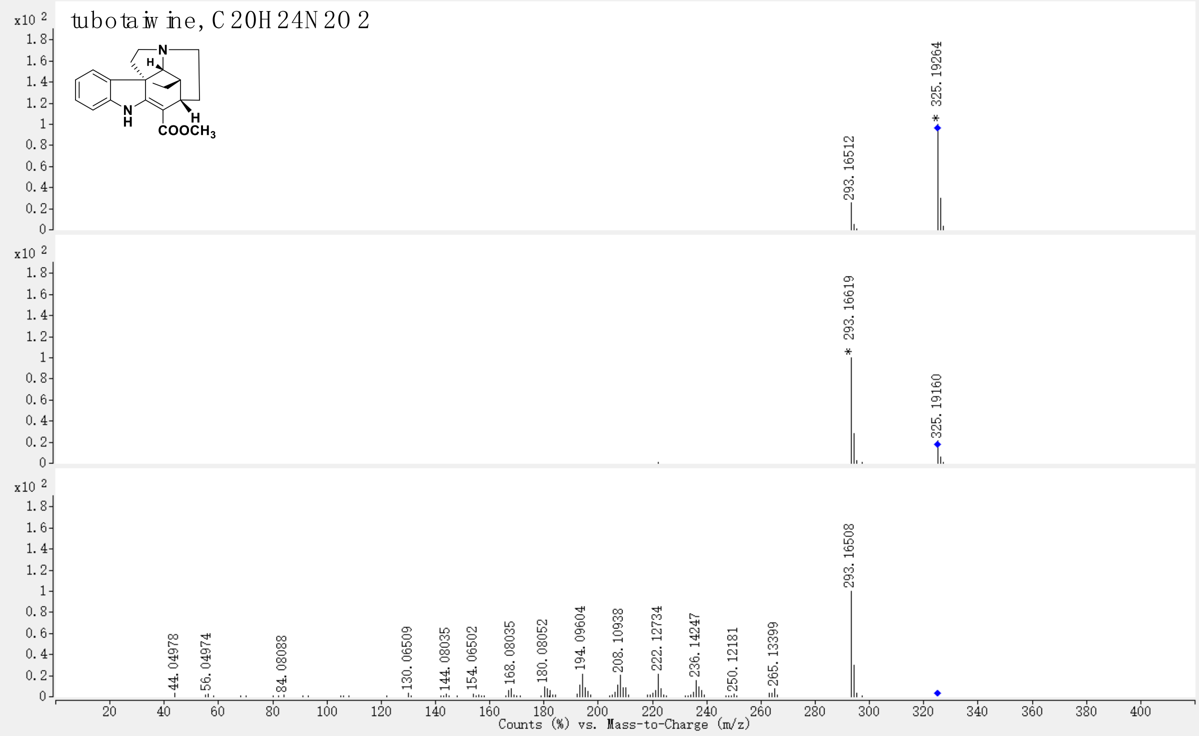

Figure S10. MCEs/MS^2 spectra of tubotaiwine (**P162**).
GNPS spectrum ID: CCMSLIB00006709974

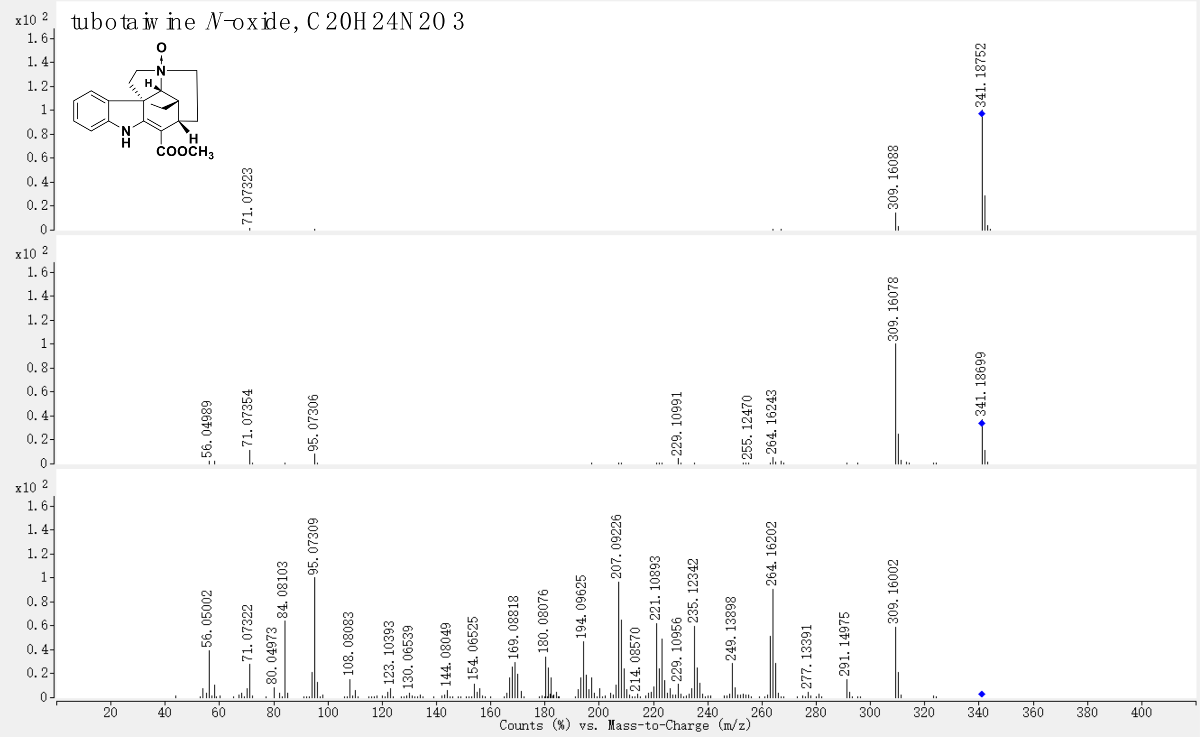

Figure S11. MCEs/MS^2 spectra of tubotaiwine *N*-oxide (**P184**).
GNPS spectrum ID: CCMSLIB00006709975

Picrinine-type MIAs

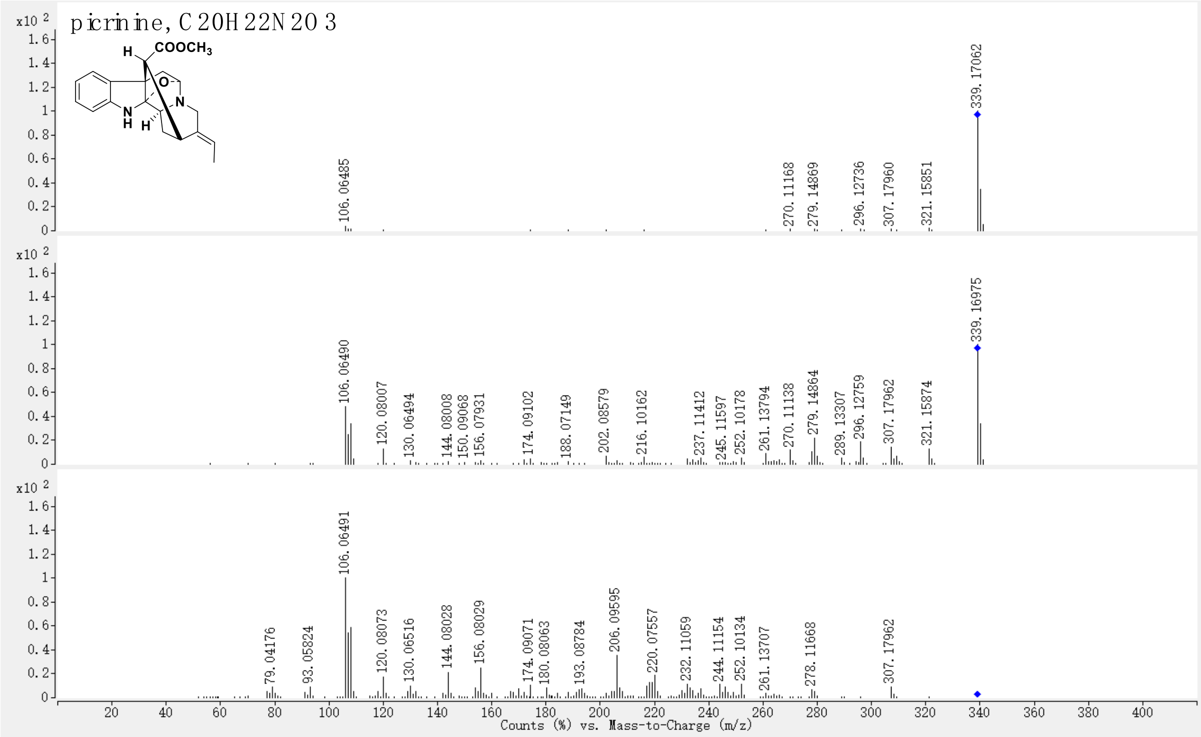

Figure S12. MCEs/MS^2 spectra of picrinine (**P160**).
GNPS spectrum ID: CCMSLIB00006709962

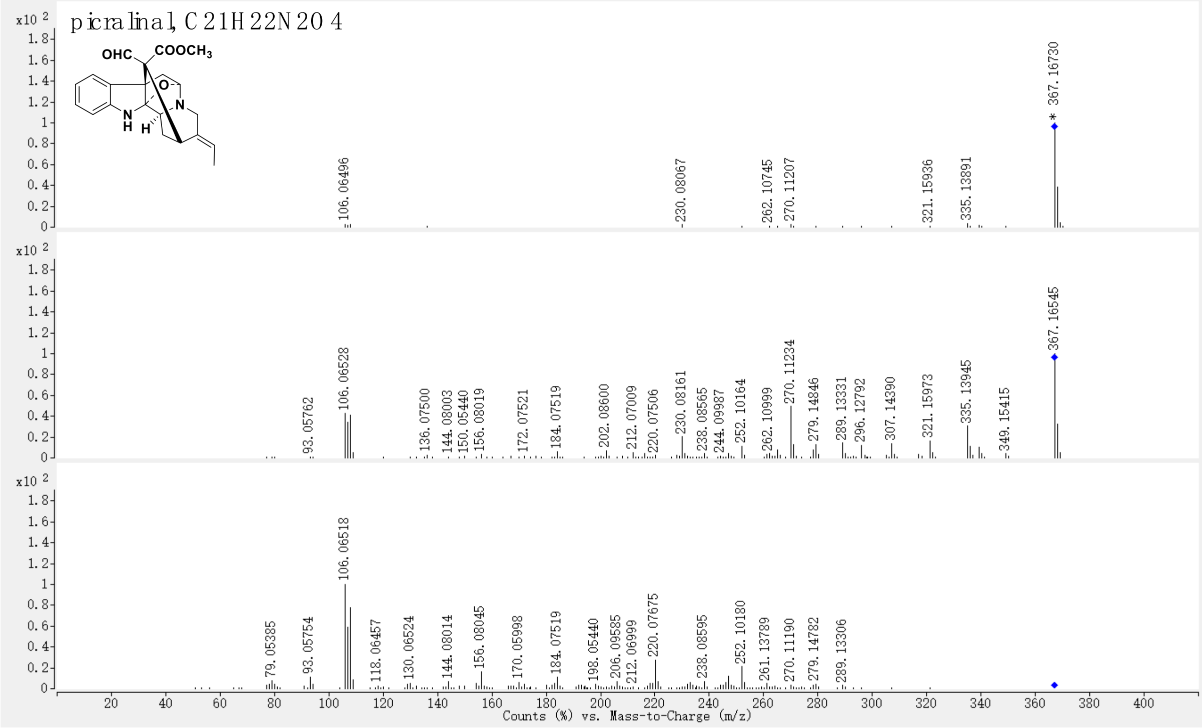

Figure S13. MCEs/MS^2 spectra of picralinal (**P169**).
GNPS spectrum ID: CCMSLIB00009919293

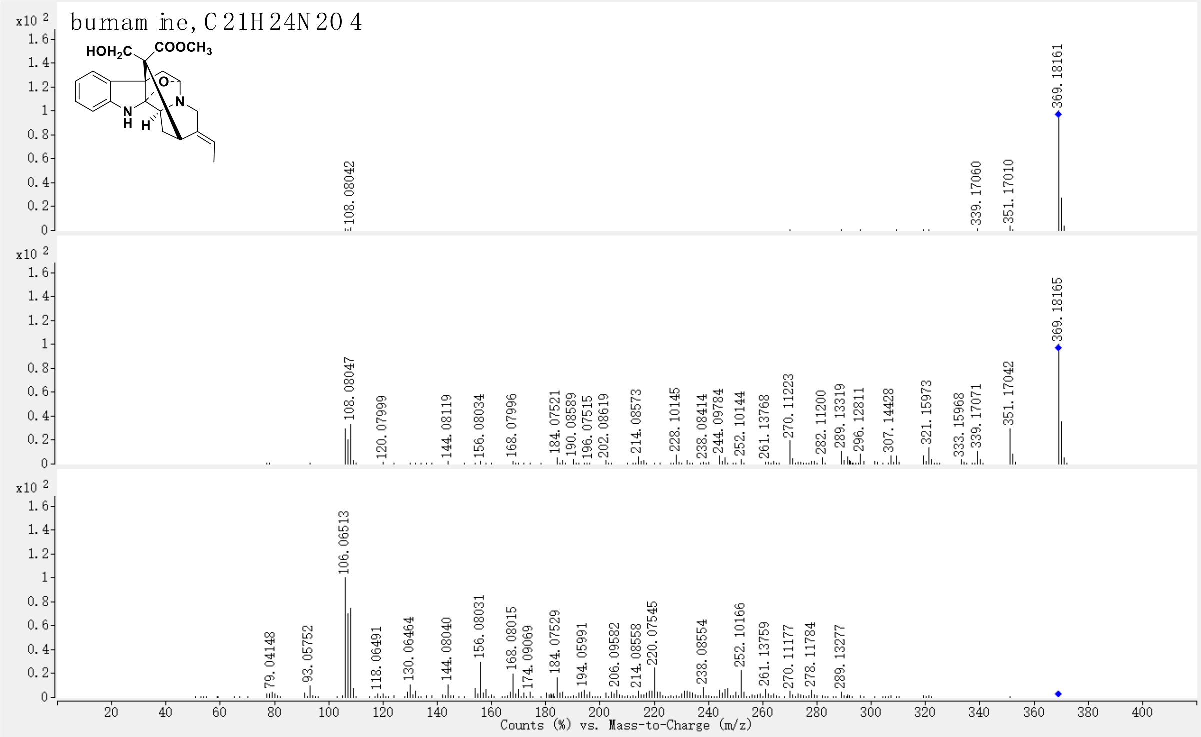

Figure S14. MCEs/MS^2 spectra of burnamine (**P96**).
GNPS spectrum ID: CCMSLIB00006709956

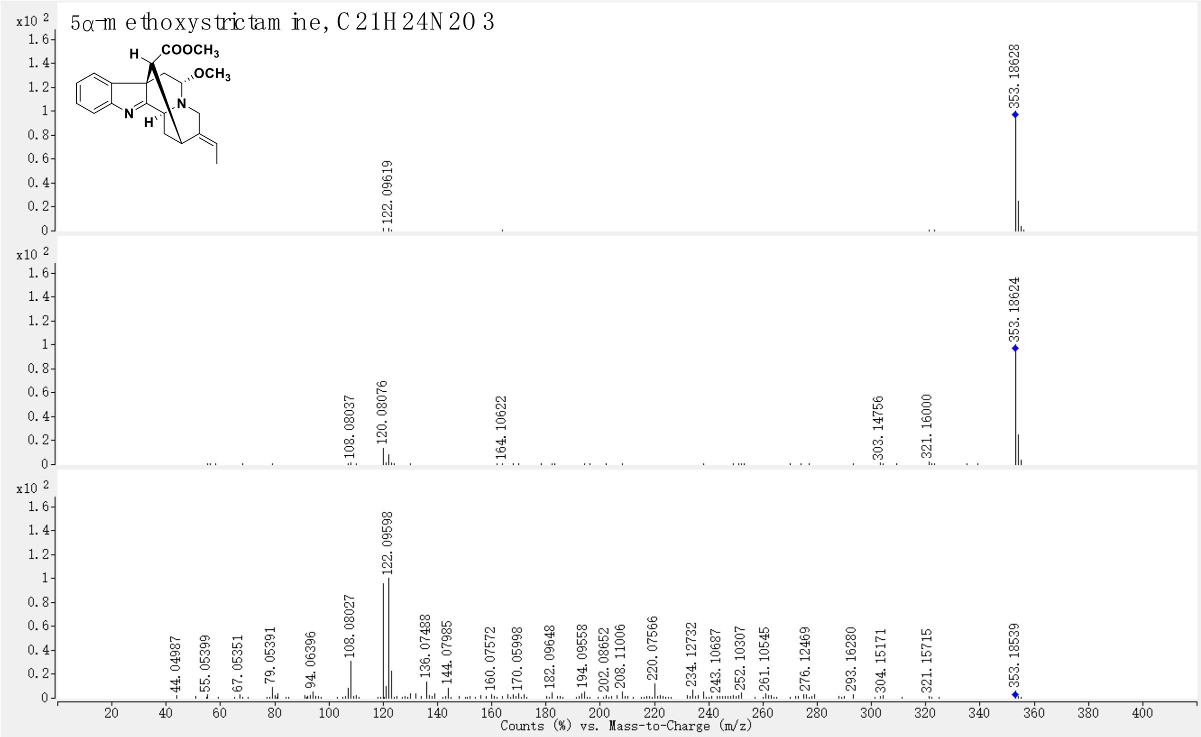

Figure S15. MCEs/MS^2 spectra of 5*α*-methoxystrictamine (**P150**).
GNPS spectrum ID: CCMSLIB00006709959

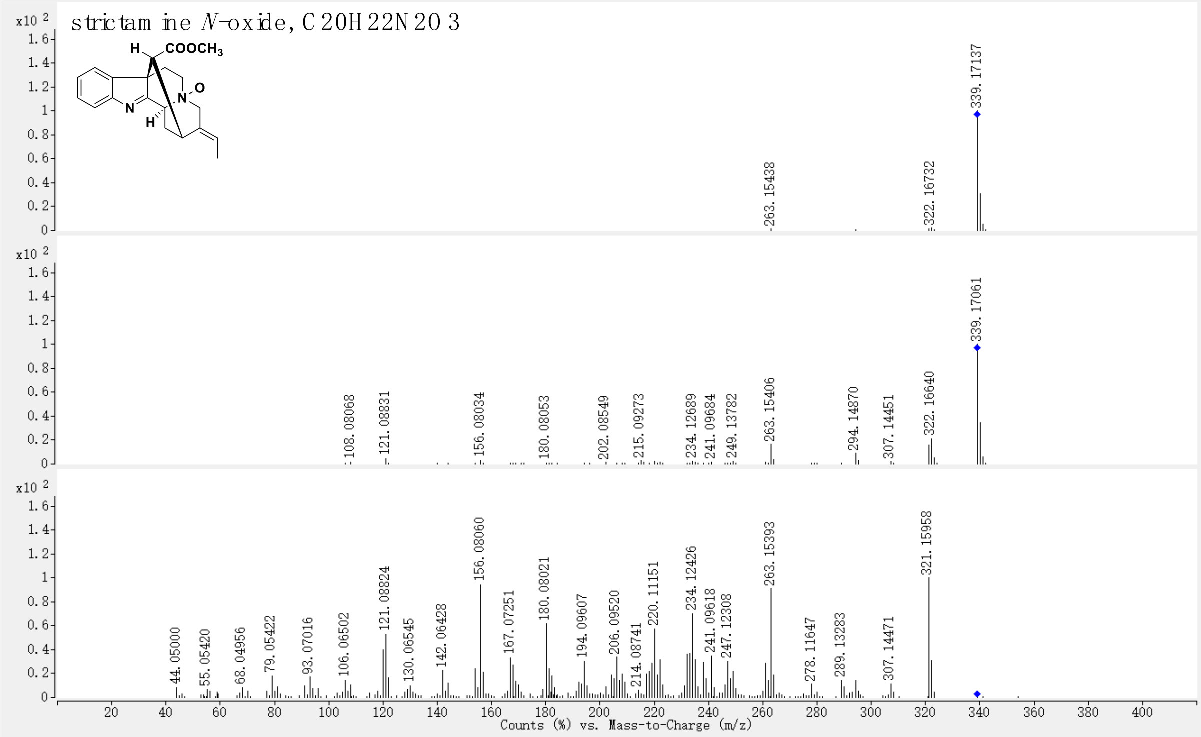

Figure S16. MCEs/MS[2] spectra of strictamine *N*-oxide (**P139**).
GNPS spectrum ID: CCMSLIB00006709958

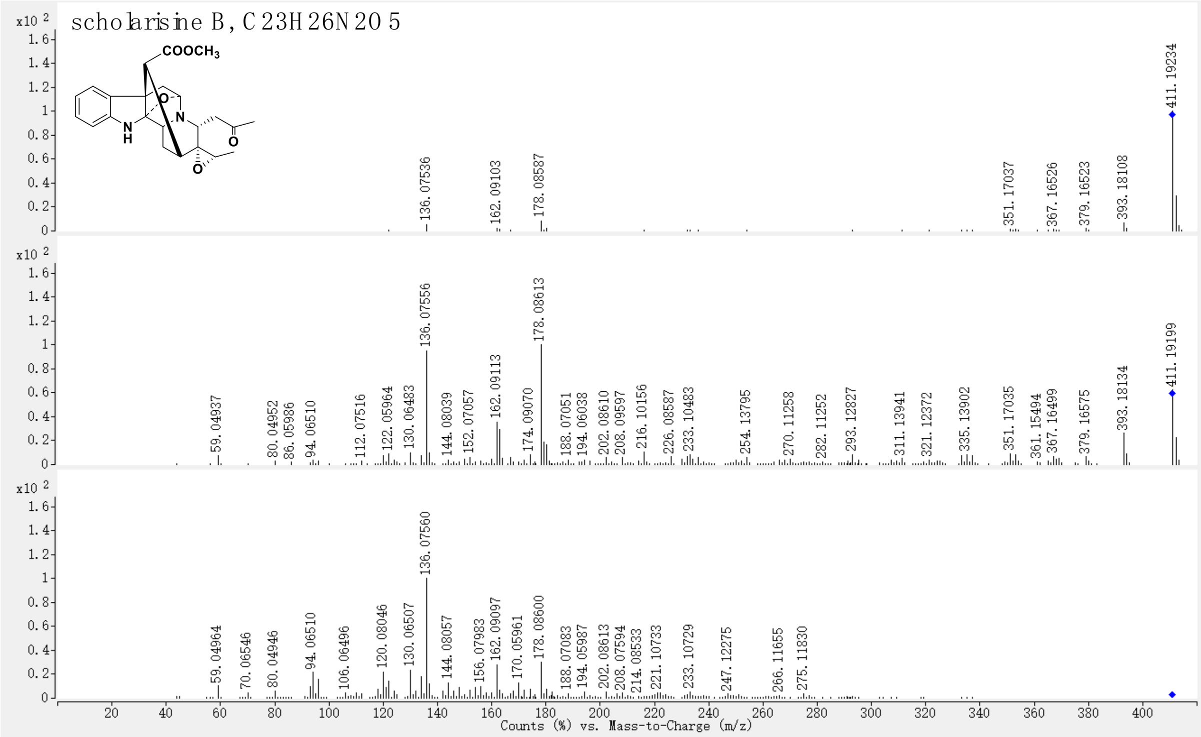

Figure S17. MCEs/MS[2] spectra of scholarisine B (**P202**).
GNPS spectrum ID: CCMSLIB00006709961

Vallesamine-type MIAs

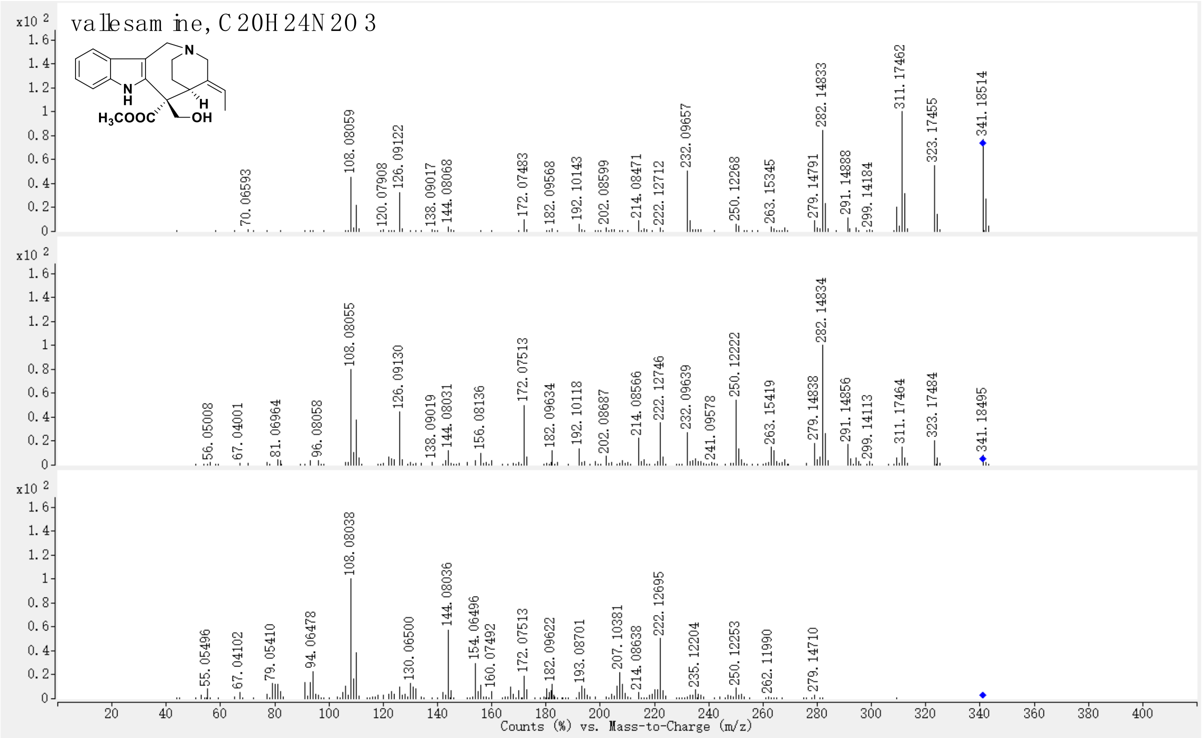

Figure S18. MCEs/MS^2 spectra of vallesamine (**P91**).
GNPS spectrum ID: CCMSLIB00006709976

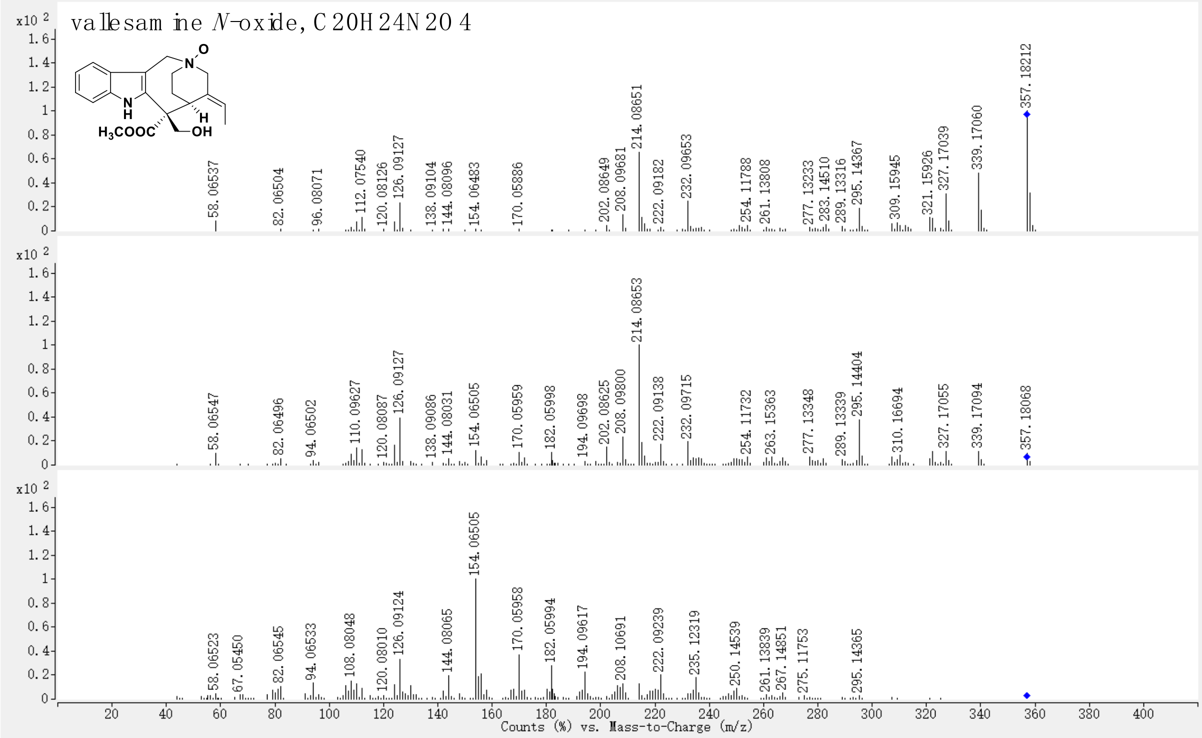

Figure S19. MCEs/MS^2 spectra of vallesamine *N*-oxide (**P116**).
GNPS spectrum ID: CCMSLIB00006709977

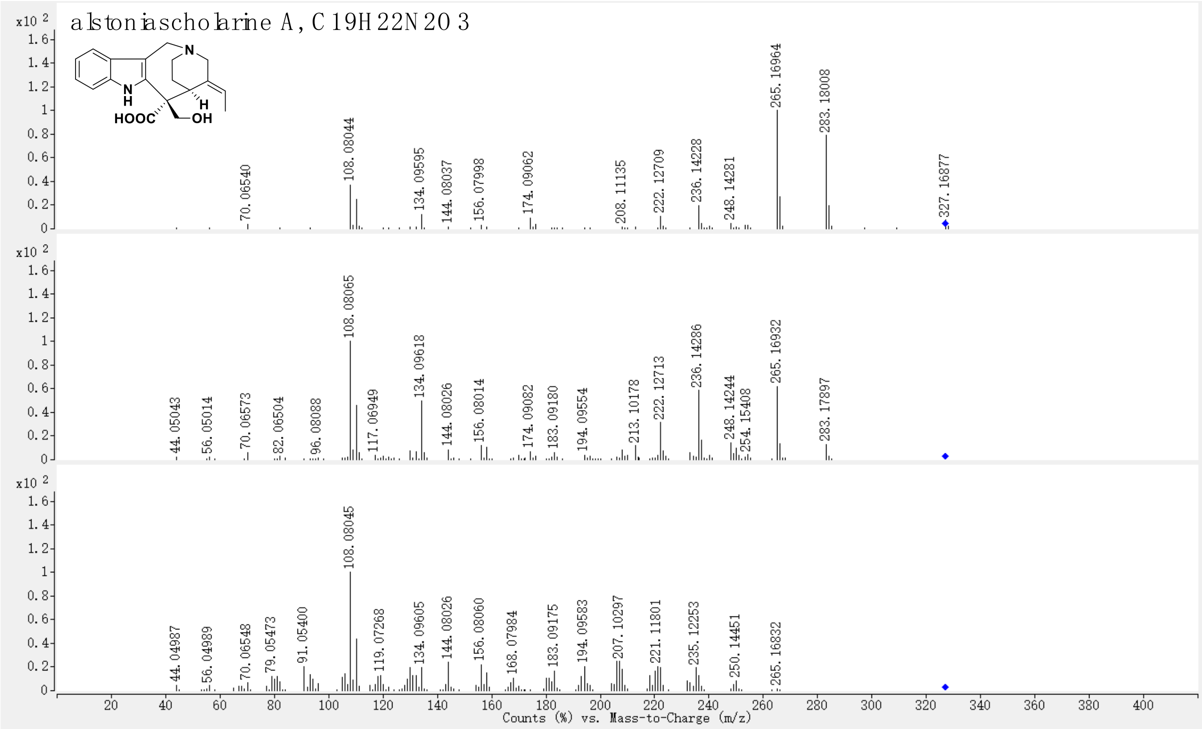

Figure S20. MCEs/MS^2 spectra of alstoniascholarine A (**P53**).
GNPS spectrum ID: CCMSLIB00009919294

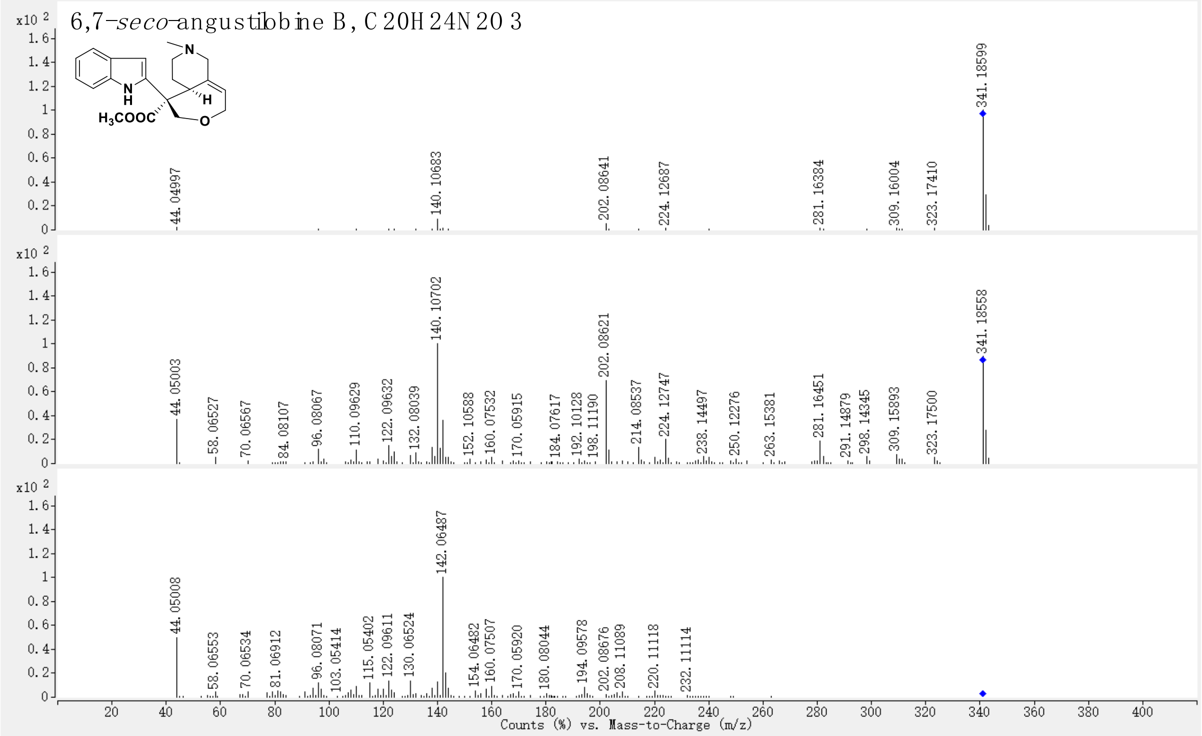

Figure S21. MCEs/MS^2 spectra of 6,7-*seco*-angustilobine B (**P137**).
GNPS spectrum ID: CCMSLIB00006709978

Alstolactine-type MIAs

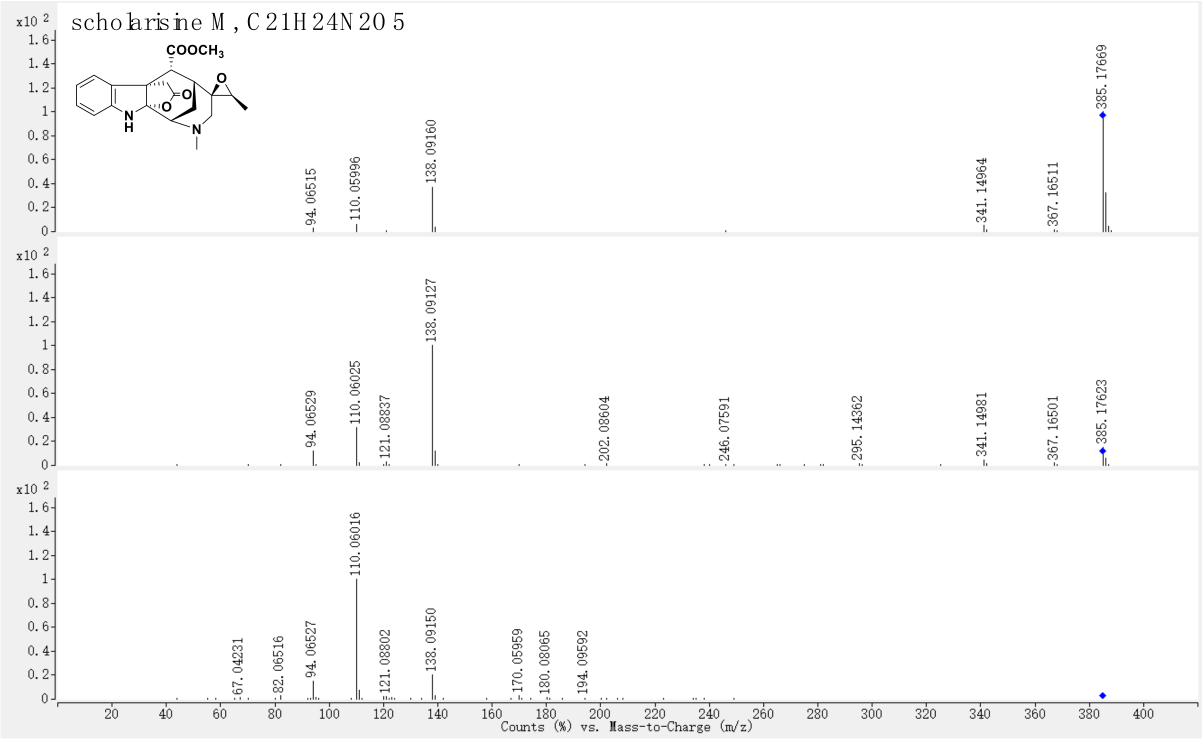

Figure S22. MCEs/MS^2 spectra of scholarisine M (**P220**).
GNPS spectrum ID: CCMSLIB00006709987

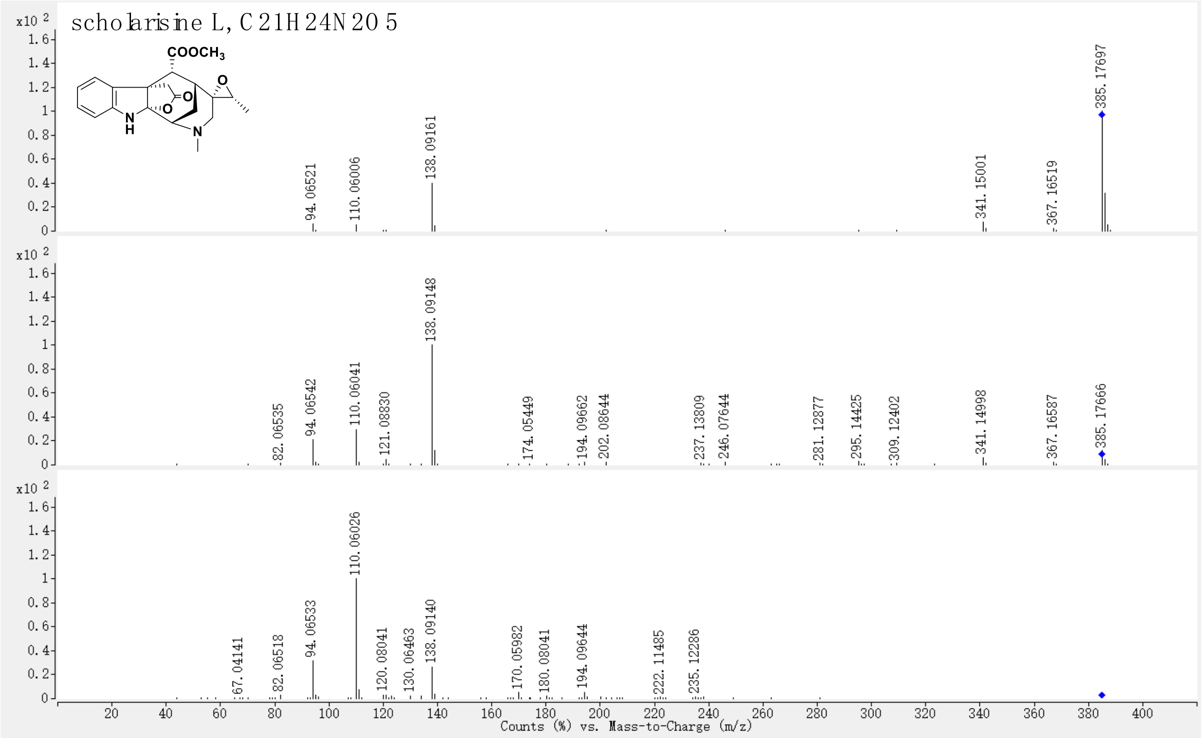

Figure S23. MCEs/MS^2 spectra of scholarisine L (**P221**).
GNPS spectrum ID: CCMSLIB00006709988

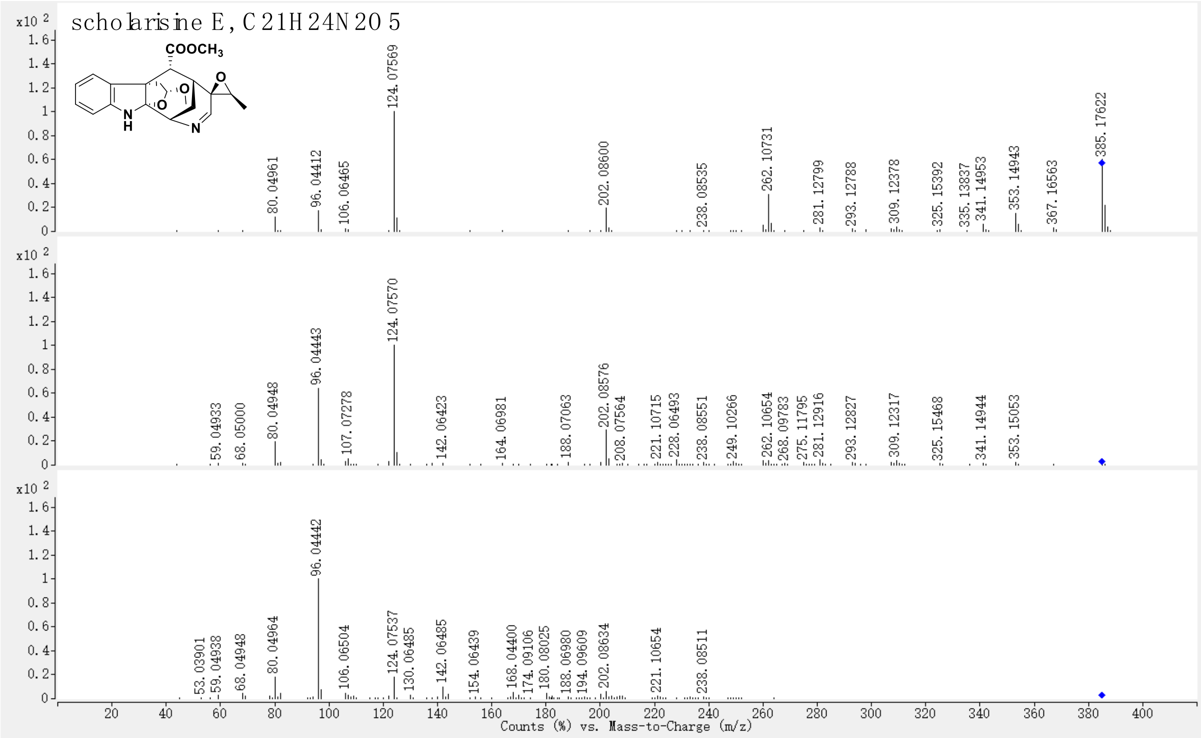

Figure S24. MCEs/MS^2 spectra of scholarisine E (**P176**).
GNPS spectrum ID: CCMSLIB00006709960

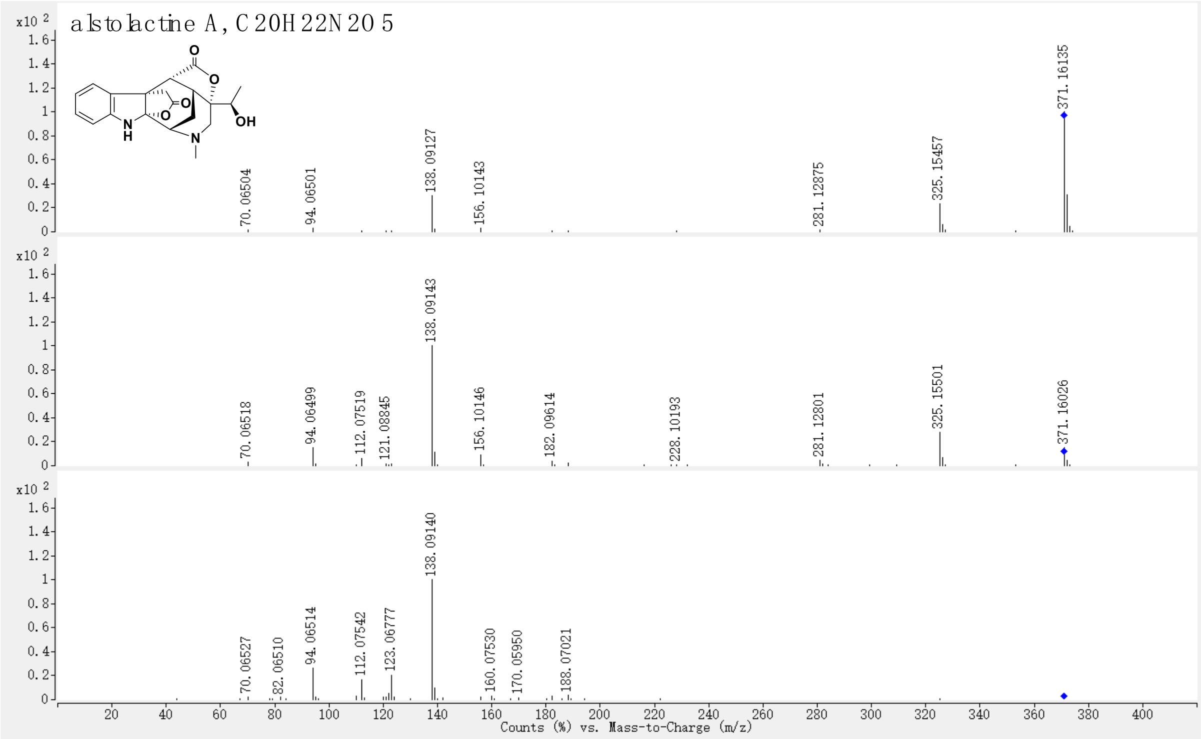

Figure S25. MCEs/MS^2 spectra of alstolactine A (**P171**).
GNPS spectrum ID: CCMSLIB00009919296

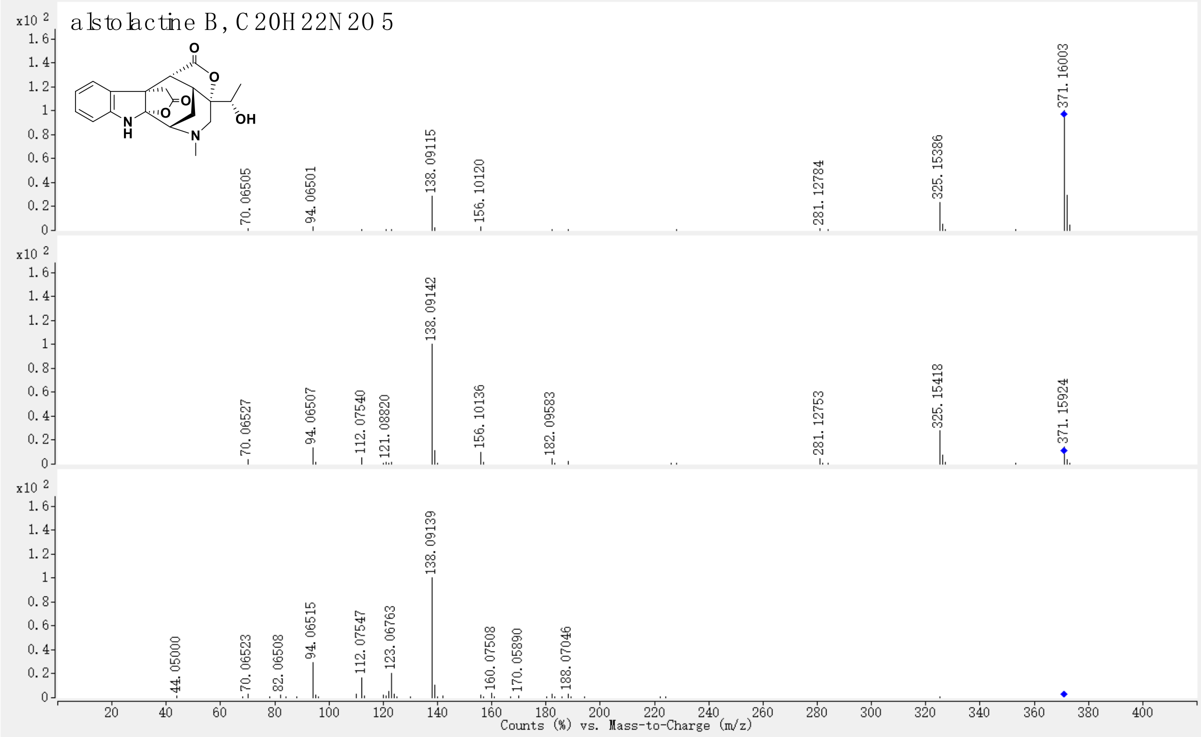

Figure S26. MCEs/MS^2 spectra of alstolactine B (**P172**).
GNPS spectrum ID: CCMSLIB00006709982

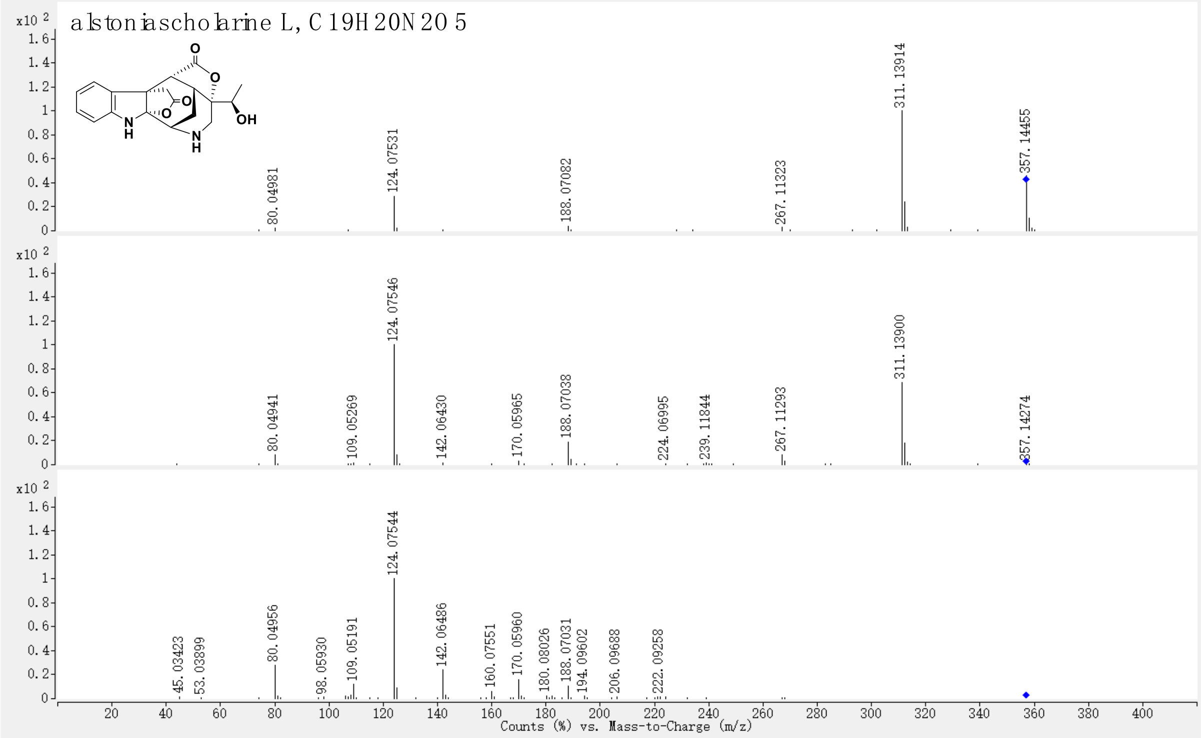

Figure S27. MCEs/MS^2 spectra of alstoniascholarine L (**P111**).
GNPS spectrum ID: CCMSLIB00006709984

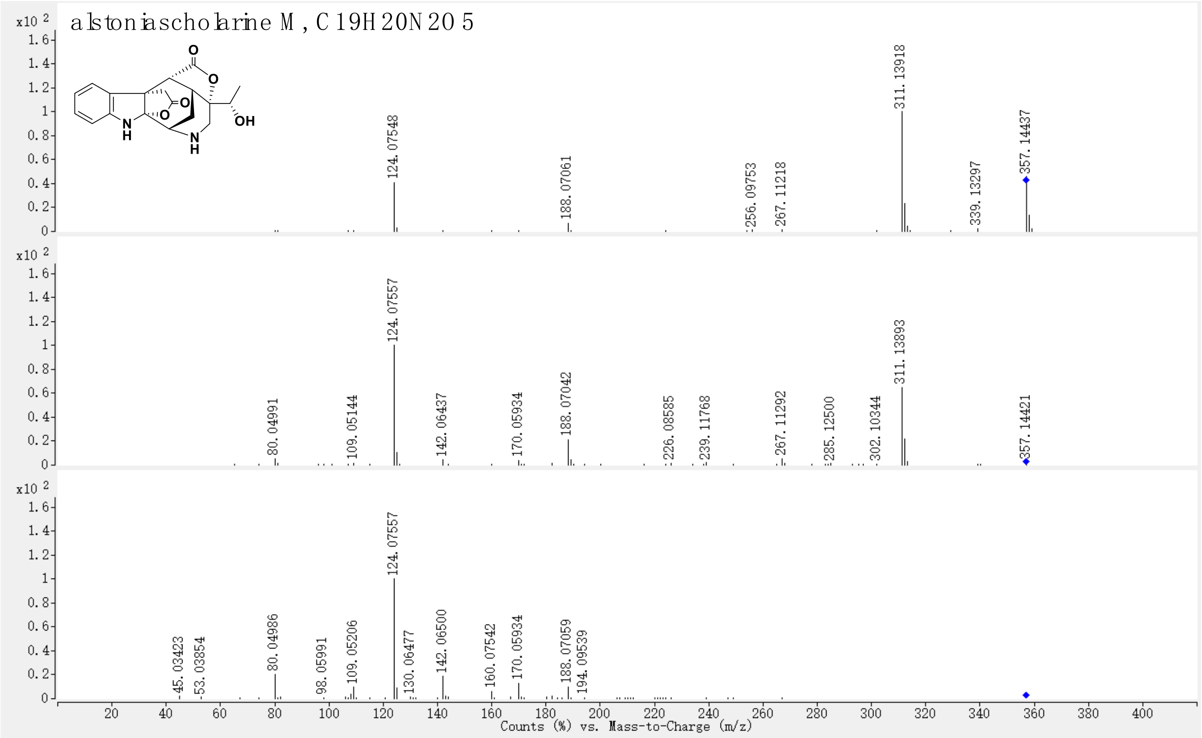

Figure S28. MCEs/MS^2 spectra of alstoniascholarine M (**P118**).
GNPS spectrum ID: CCMSLIB00006709985

Yohimbine-type MIAs

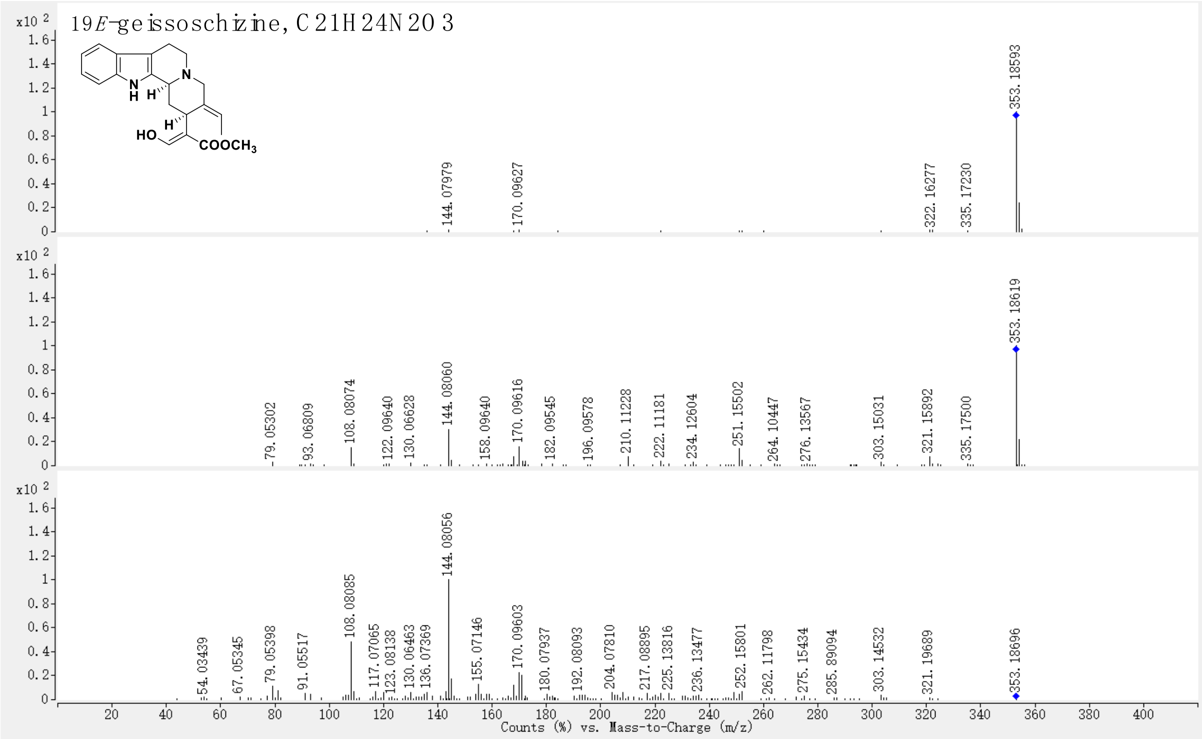

Figure S29. MCEs/MS^2 spectra of 19*E*-geissoschizine (**P147**).
GNPS spectrum ID: CCMSLIB00006710009

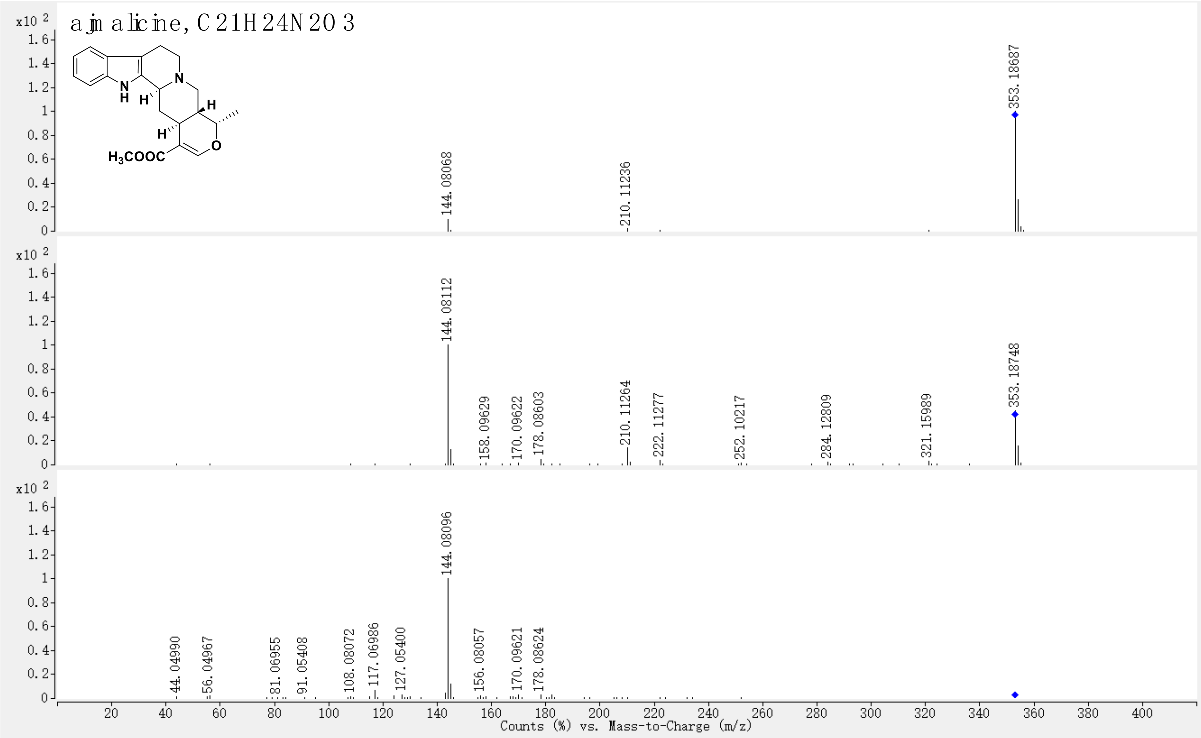

Figure S30. MCEs/MS^2 spectra of ajmalicine (**P157**).
GNPS spectrum ID: CCMSLIB00006710010

Alstoscholarisine-type MIAs

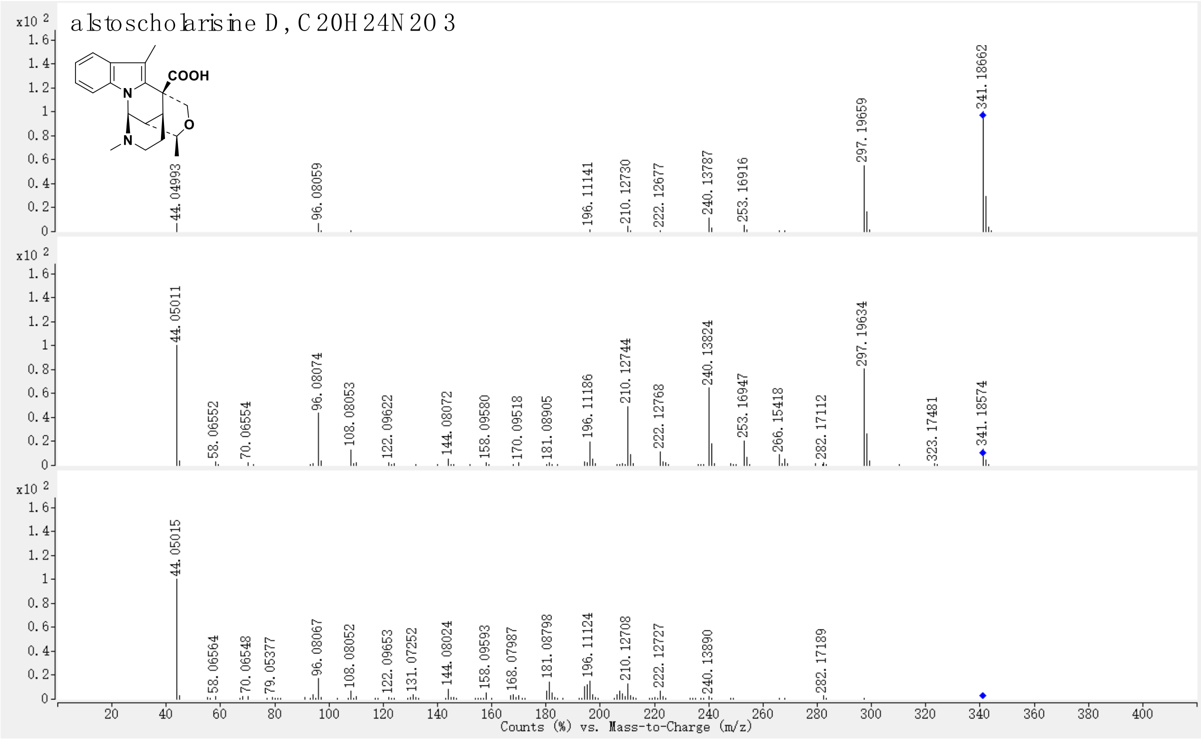

Figure S31. MCEs/MS^2 spectra of alstoscholarisine D (**P95**).
GNPS spectrum ID: CCMSLIB00006709989

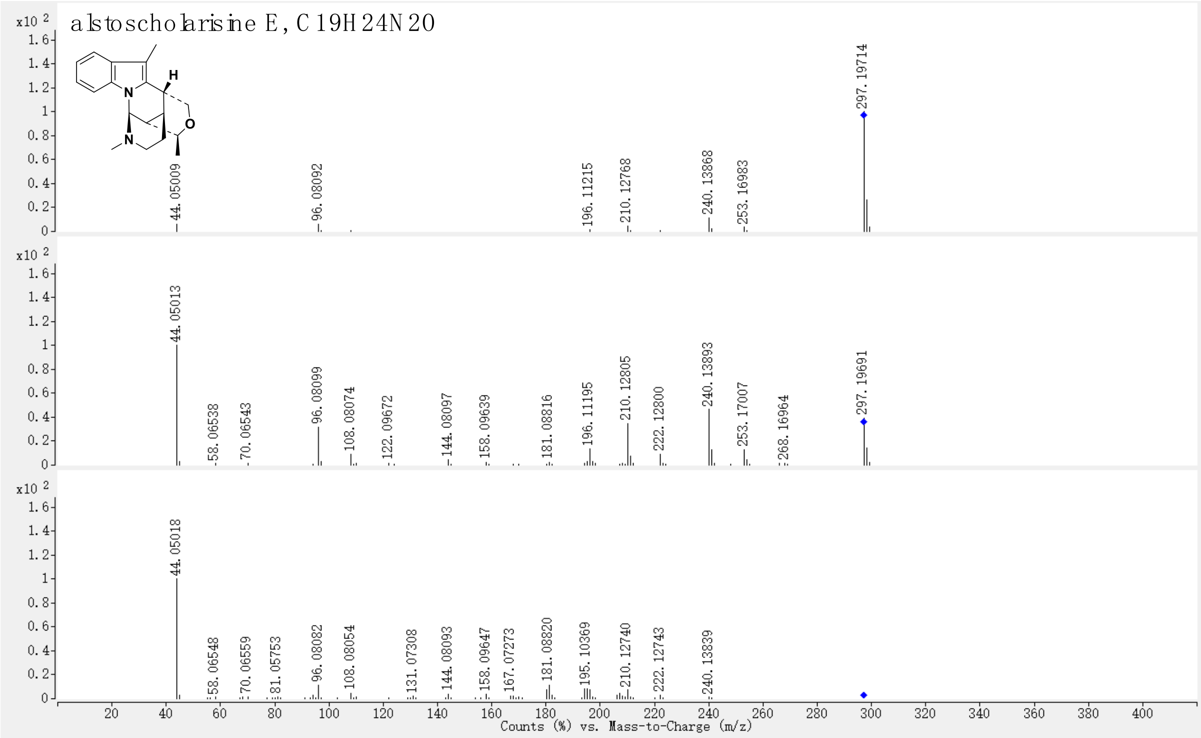

Figure S32. MCEs/MS^2 spectra of alstoscholarisine E (**P141**).

GNPS spectrum ID: CCMSLIB00006709990

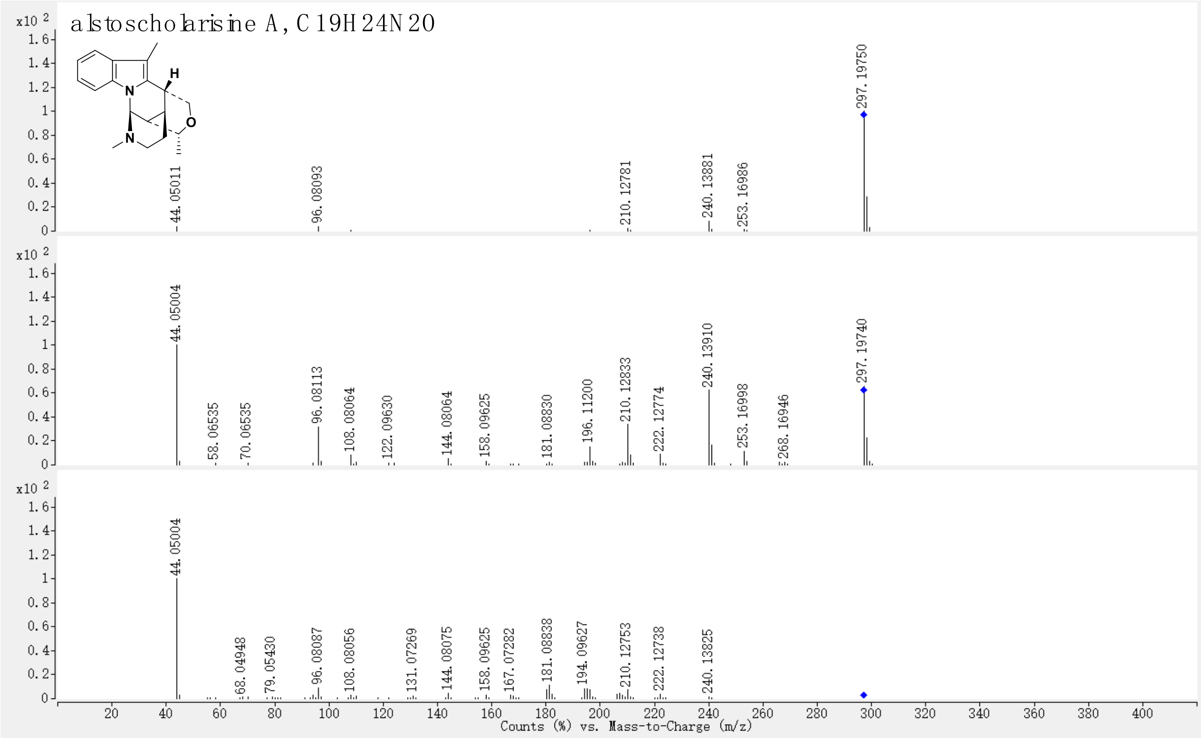

Figure S33. MCEs/MS^2 spectra of alstoscholarisine A (**P124**).

GNPS spectrum ID: CCMSLIB00006709991

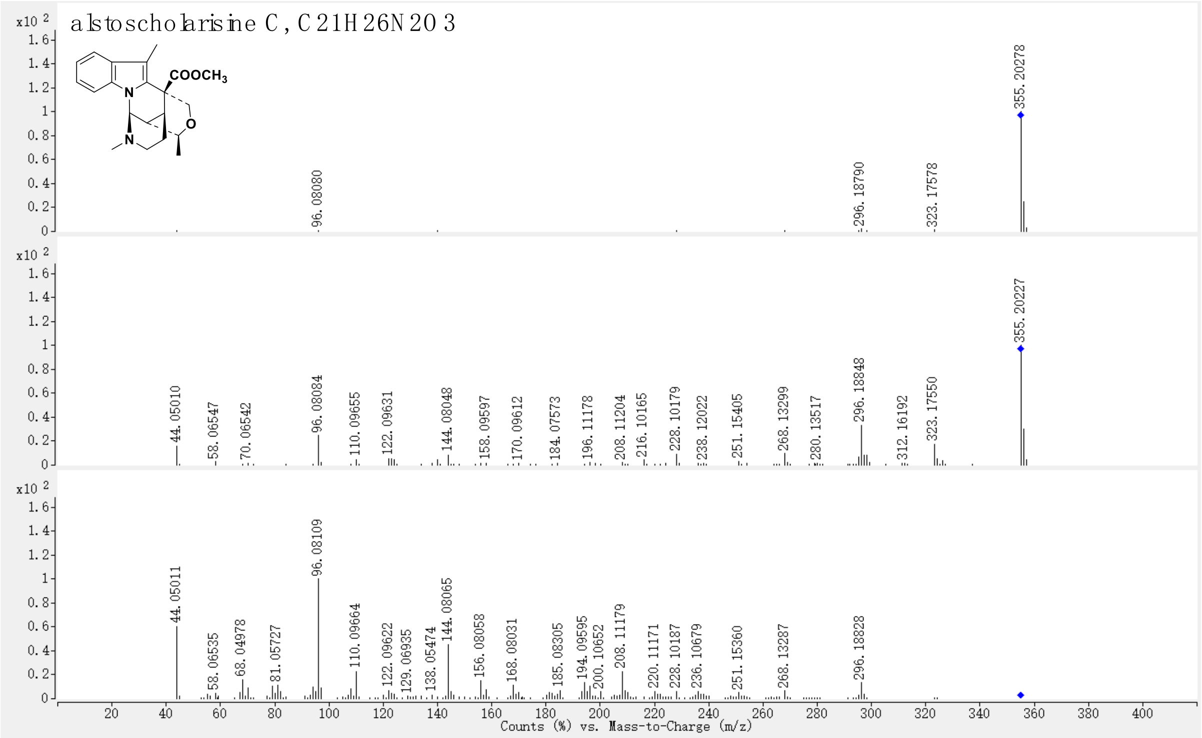

Figure S34. MCEs/MS^2 spectra of alstoscholarisine C (**P179**).
GNPS spectrum ID: CCMSLIB00006709992

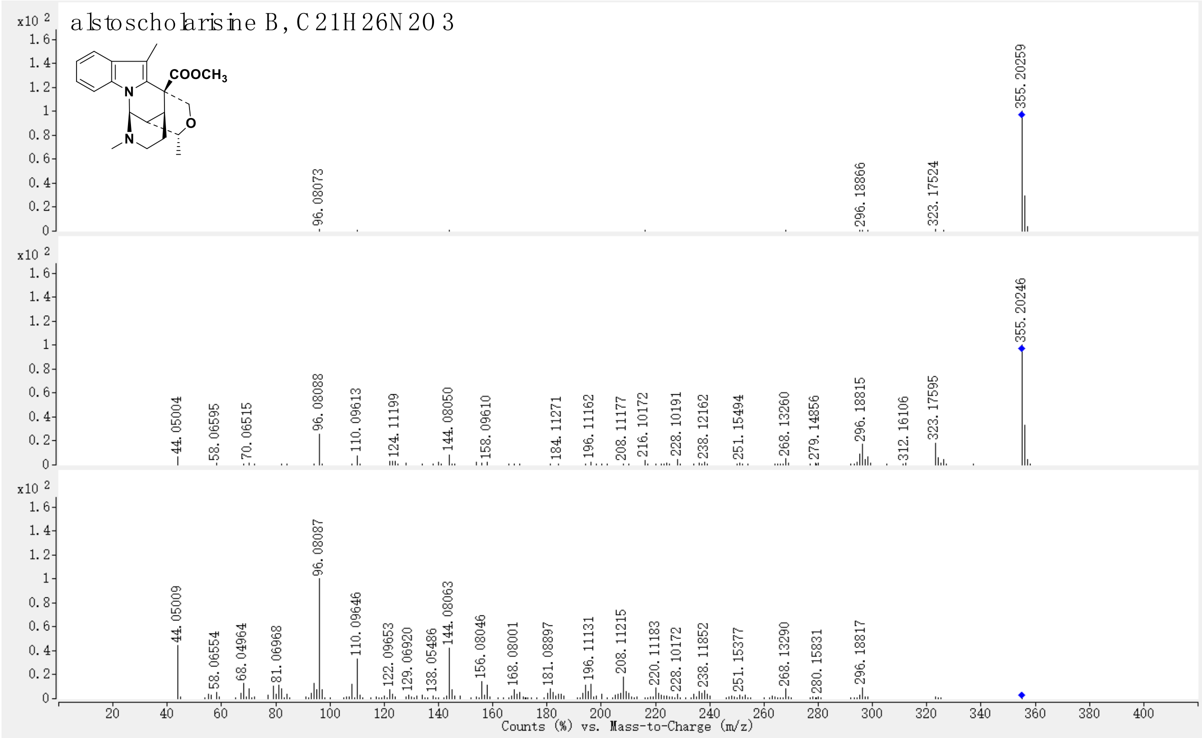

Figure S35. MCEs/MS^2 spectra of alstoscholarisine B (**P187**).
GNPS spectrum ID: CCMSLIB00006709993

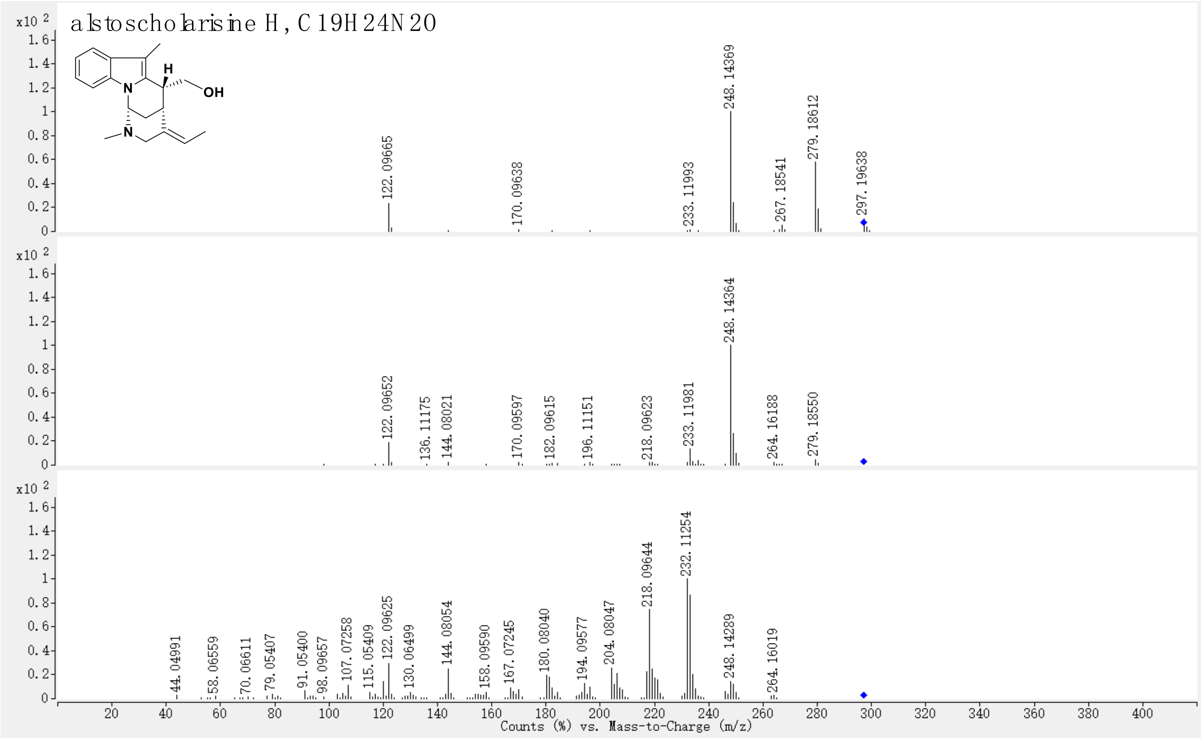

Figure S36. MCEs/MS^2 spectra of alstoscholarisine H (**P112**).
GNPS spectrum ID: CCMSLIB00006709995

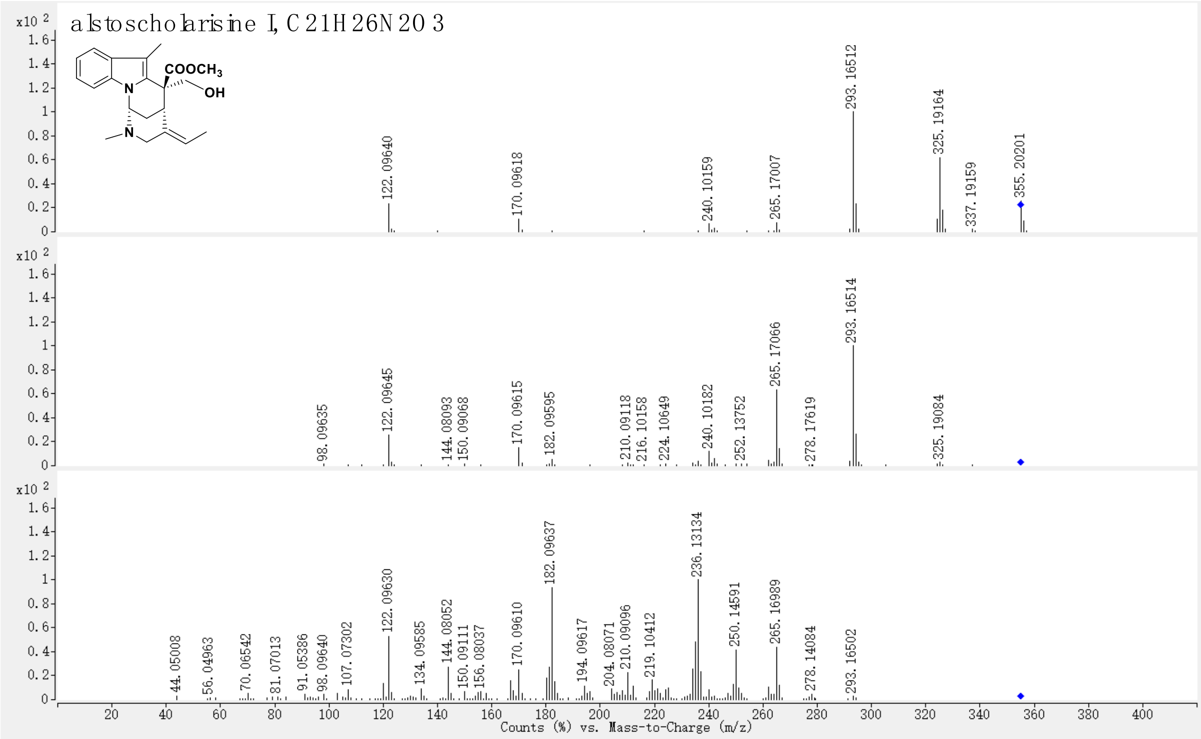

Figure S37. MCEs/MS^2 spectra of alstoscholarisine I (**P135**).
GNPS spectrum ID: CCMSLIB00006709994

Scholarisine-type MIAs

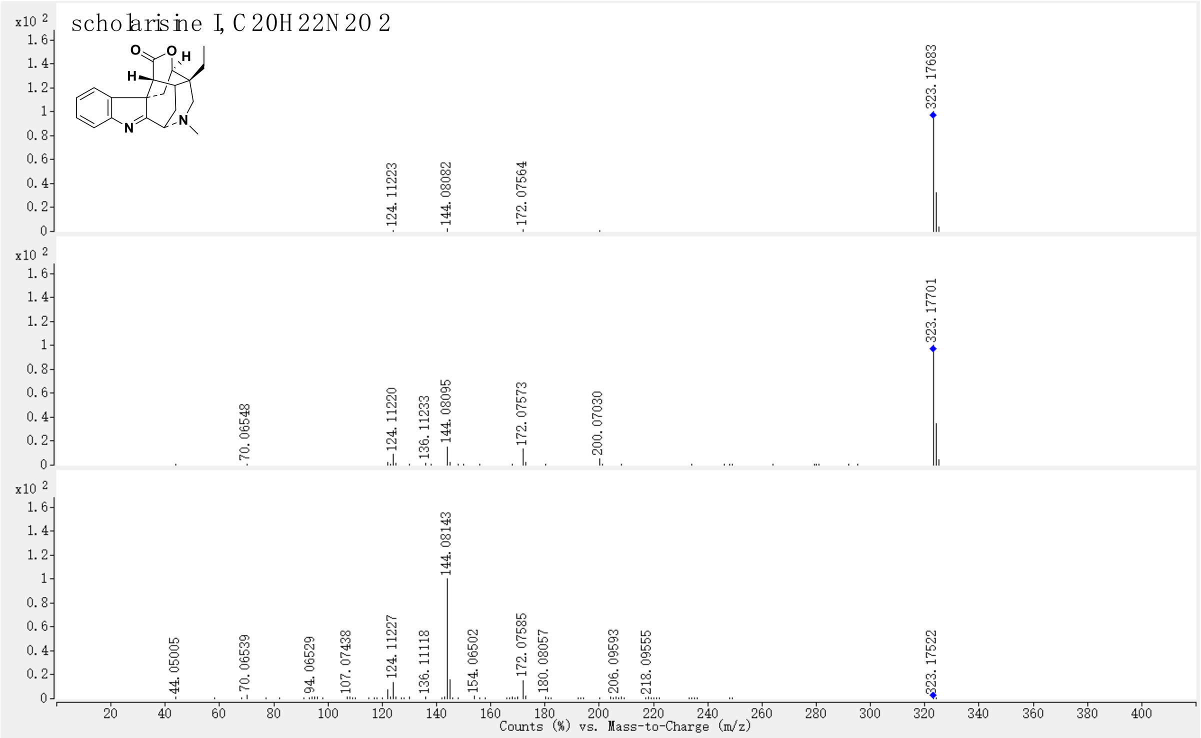

Figure S38. MCEs/MS^2 spectra of scholarisine I (**P67**).
GNPS spectrum ID: CCMSLIB00006709979

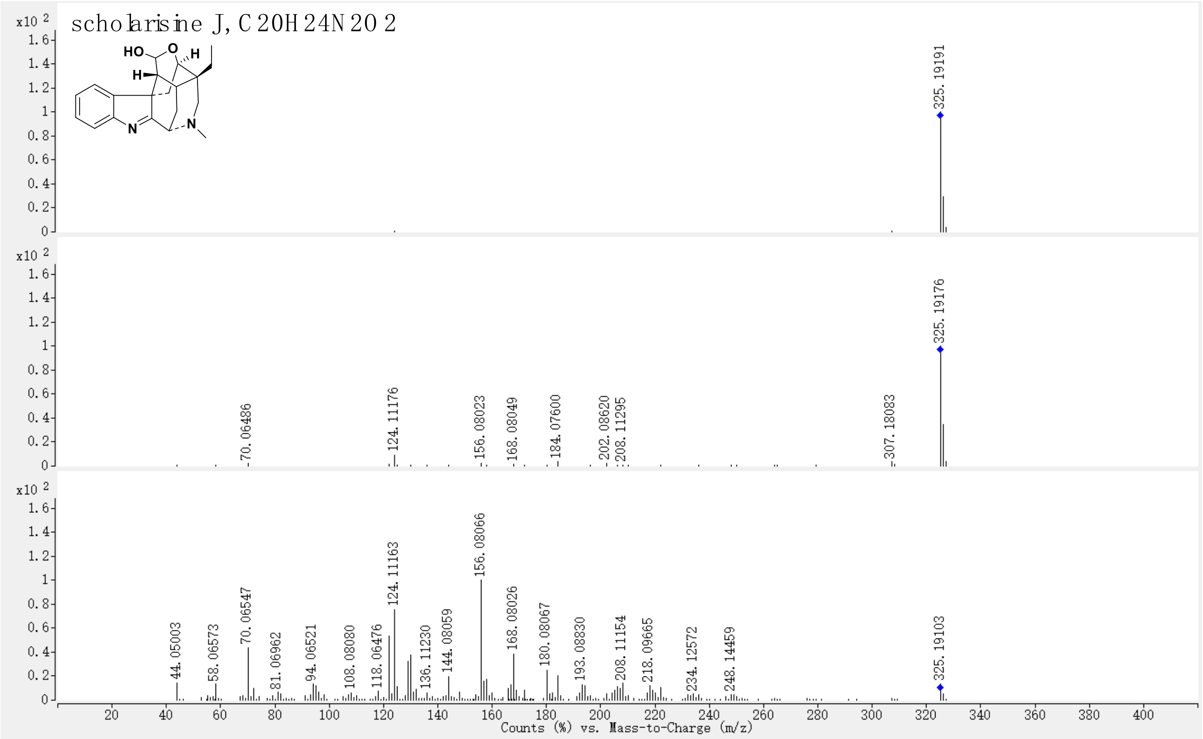

Figure S39. MCEs/MS^2 spectra of scholarisine J (**P10**).
GNPS spectrum ID: CCMSLIB00006709980

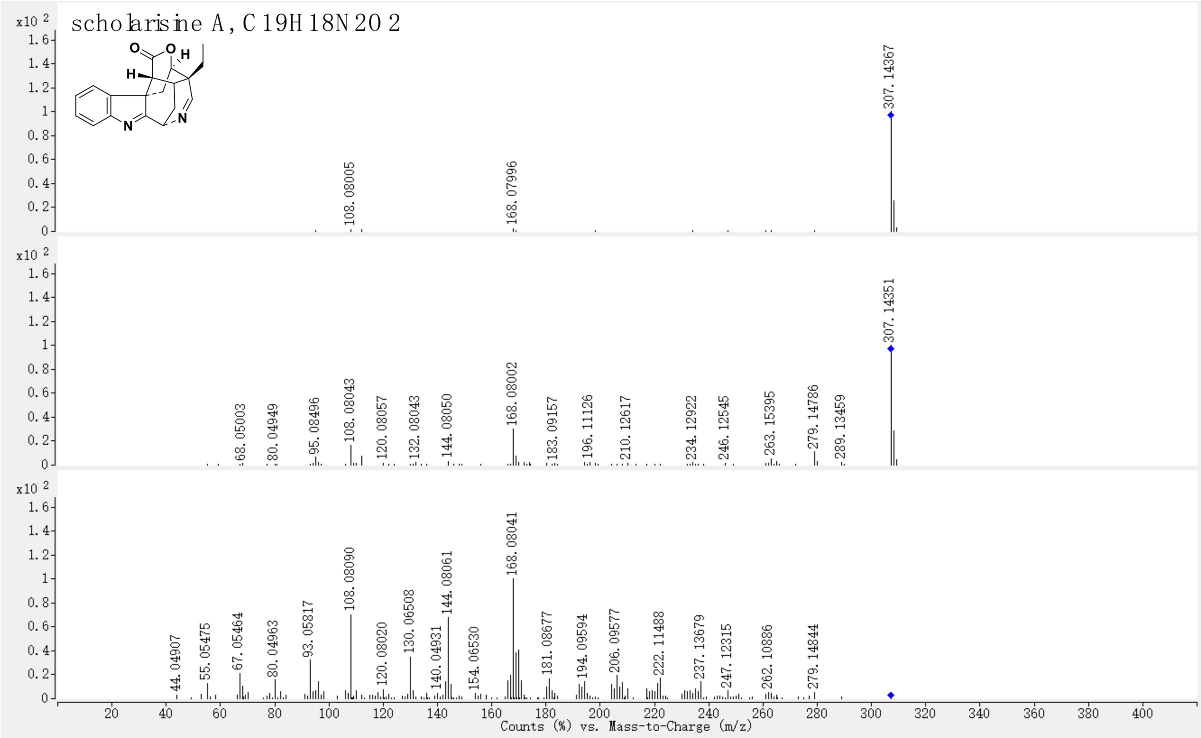

Figure S40. MCEs/MS^2 spectra of scholarisine A (**P158**).

GNPS spectrum ID: CCMSLIB00009919295

Vallesiachotamine-type MIAs

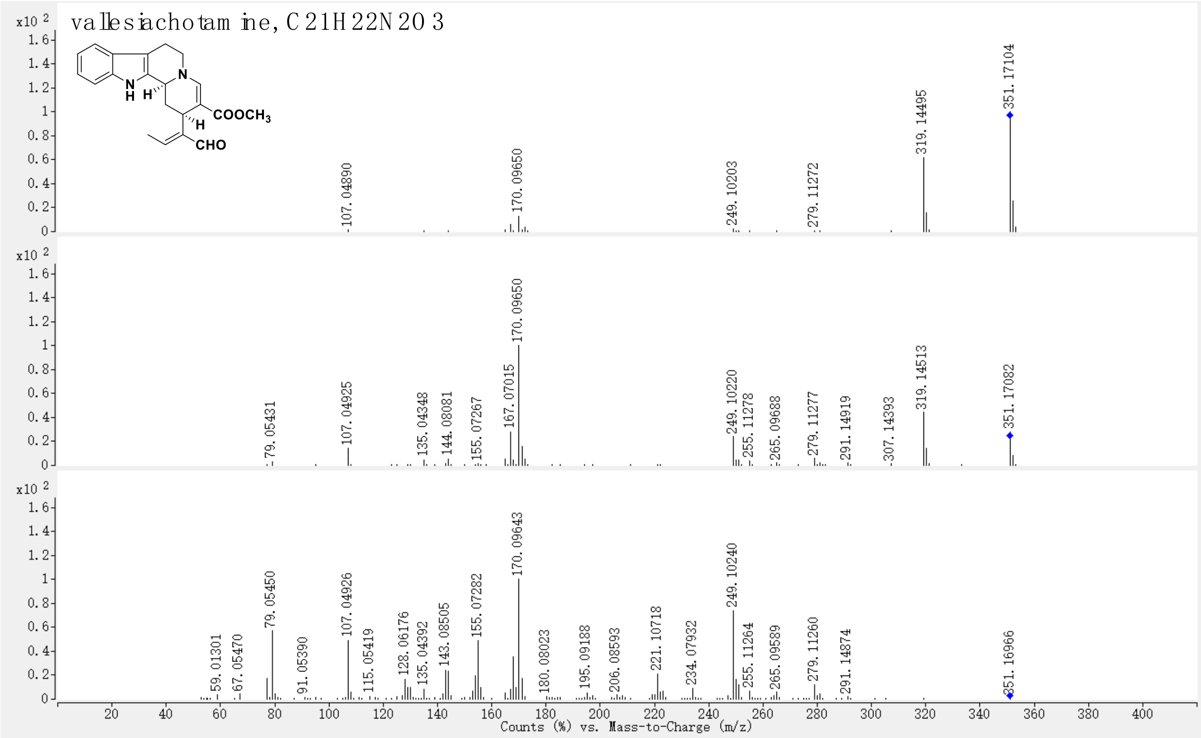

Figure S41. MCEs/MS^2 spectra of vallesiachotamine (**P225**).

GNPS spectrum ID: CCMSLIB00006709998

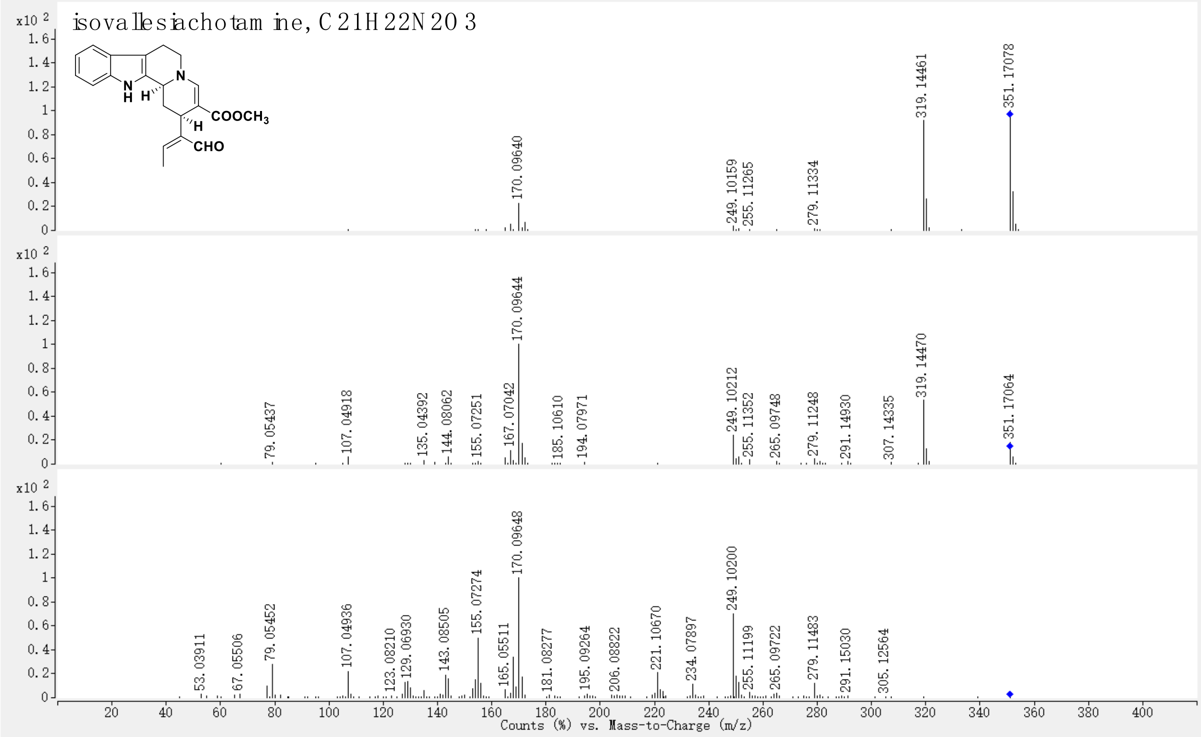

Figure S42. MCEs/MS[2] spectra of isovallesiachotamine (**P226**).
GNPS spectrum ID: CCMSLIB00006709999

Alstoscholarine-type MIAs

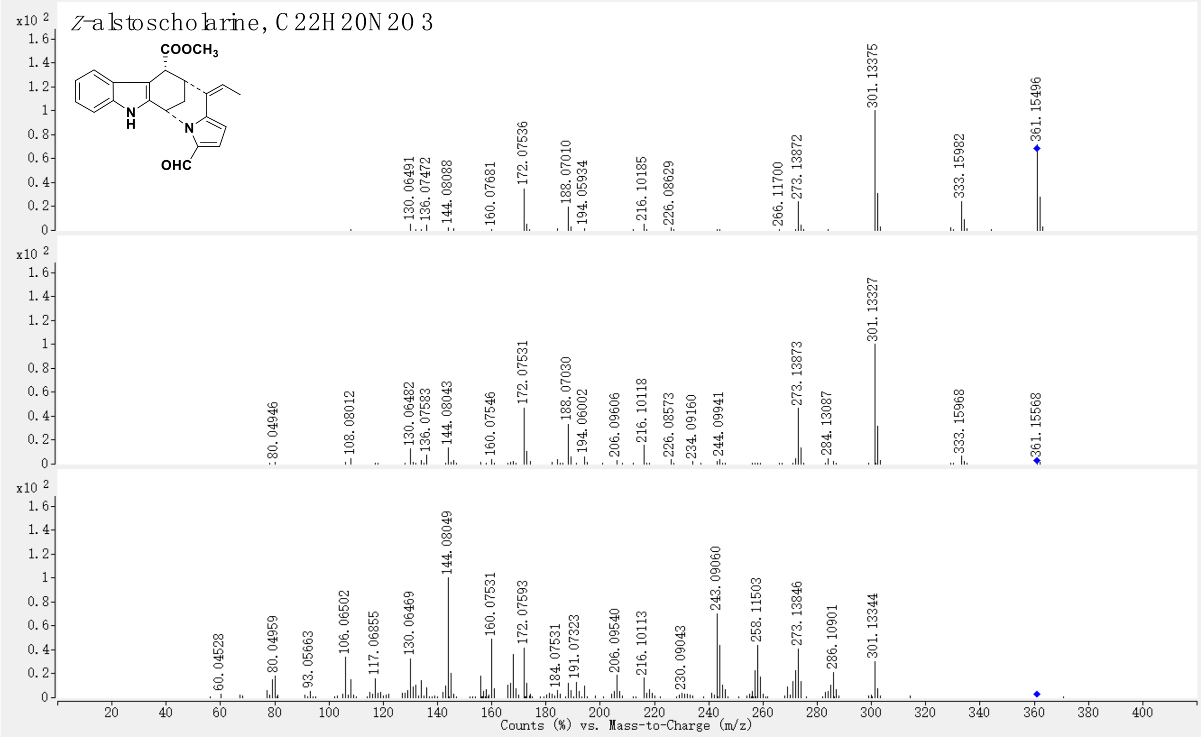

Figure S43. MCEs/MS[2] spectra of *Z*-alstoscholarine (**P227**).
GNPS spectrum ID: CCMSLIB00006709996

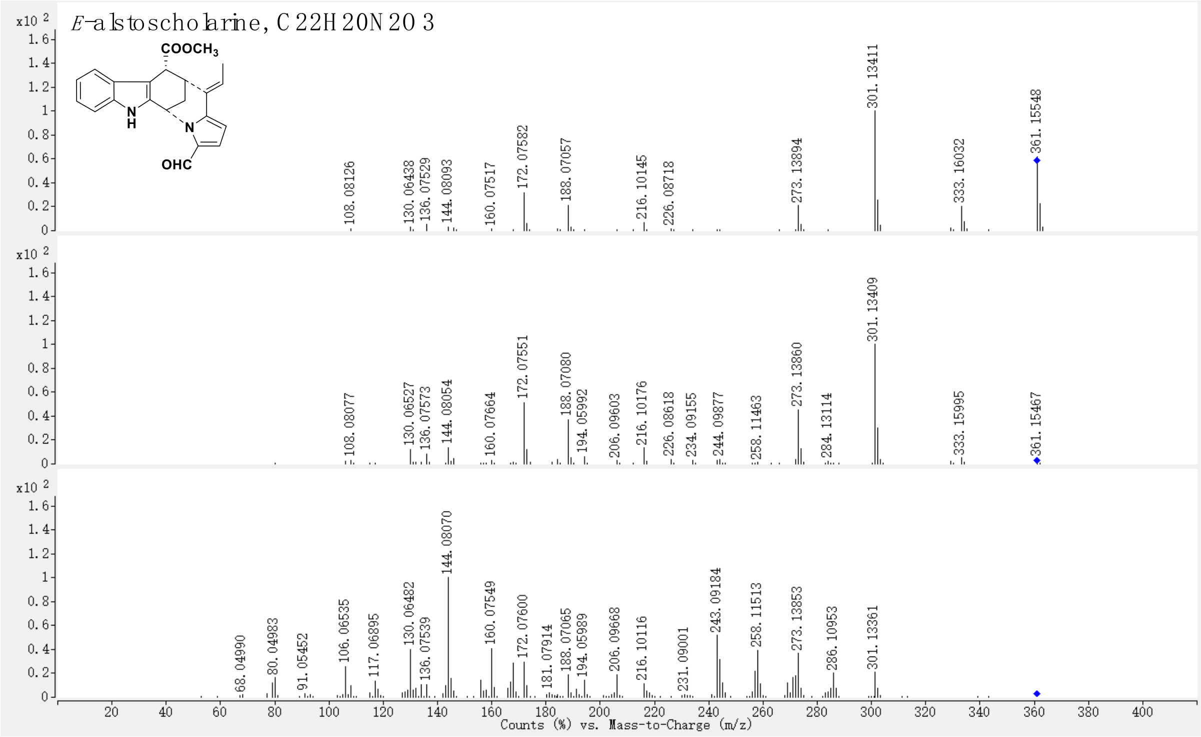

Figure S44. MCEs/MS^2 spectra of *E*-alstoscholarine (**P228**).
GNPS spectrum ID: CCMSLIB00006709997

Unclassified MIAs

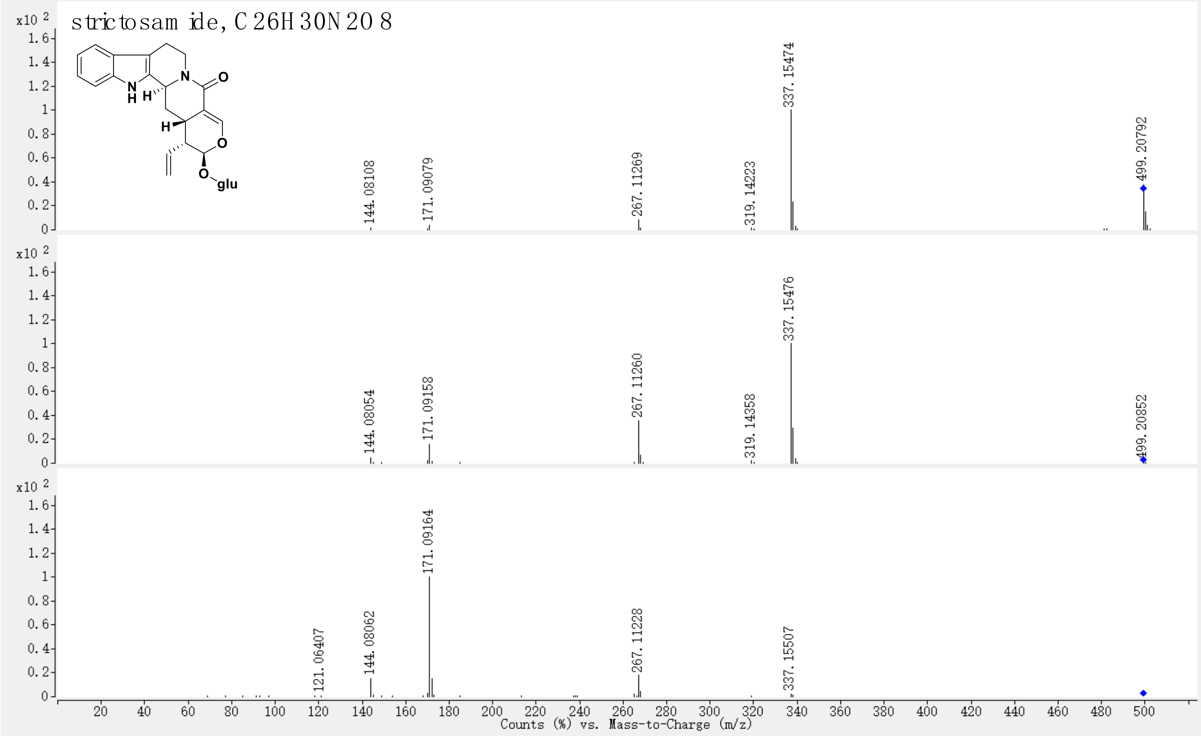

Figure S45. MCEs/MS^2 spectra of strictosamide (**P188**).
GNPS spectrum ID: CCMSLIB00006710011

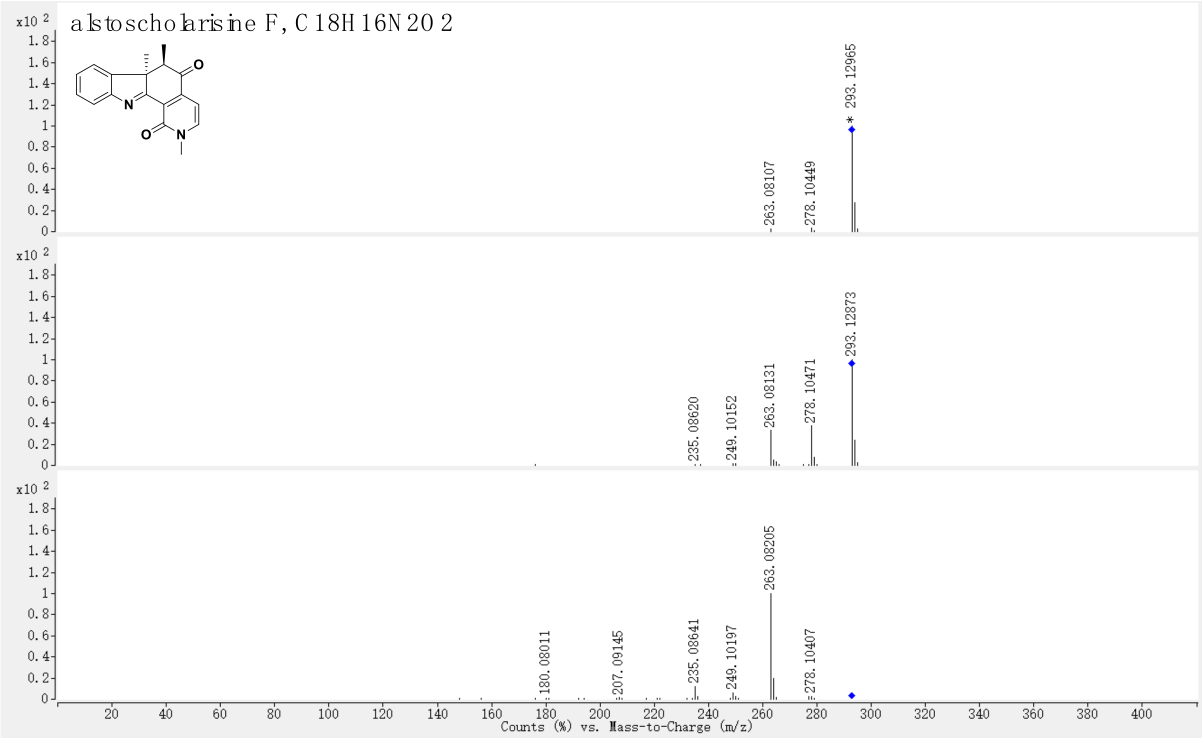

Figure S46. MCEs/MS[2] spectra of alstoscholarisine F (**P159**).
GNPS spectrum ID: CCMSLIB00006710013

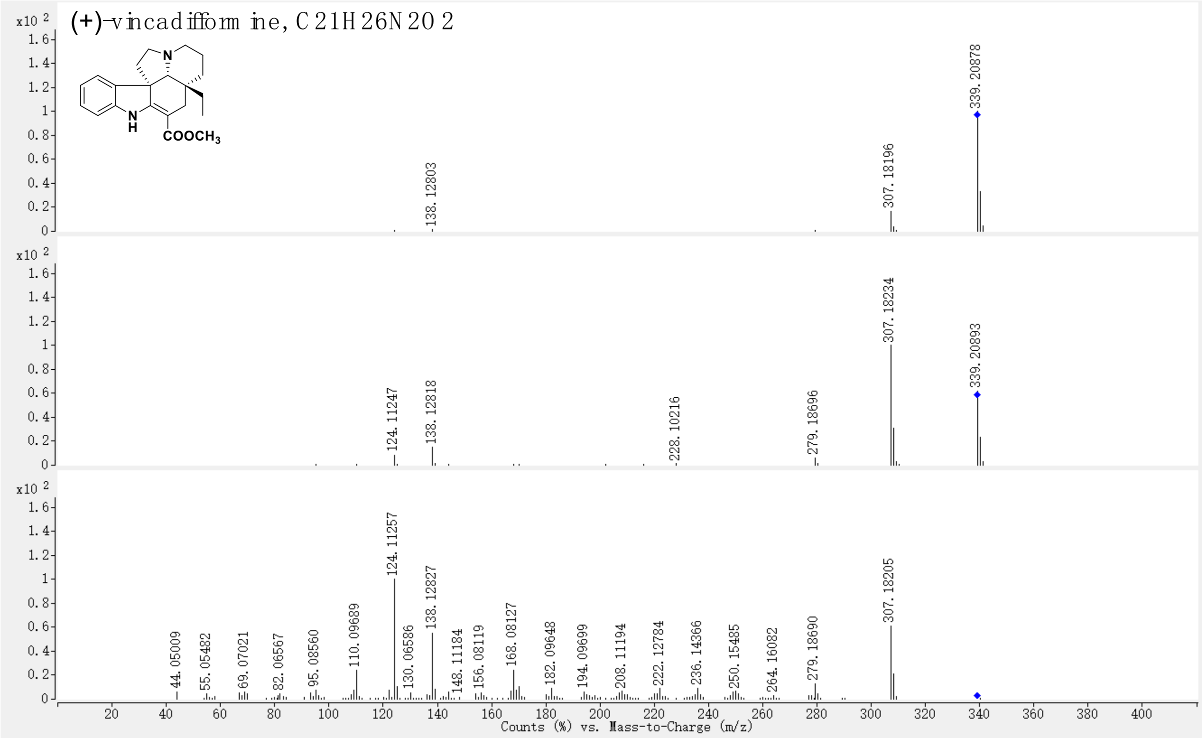

Figure S47. MCEs/MS[2] spectra of (+)-vincadifformine (**P181**).
GNPS spectrum ID: CCMSLIB00006710015

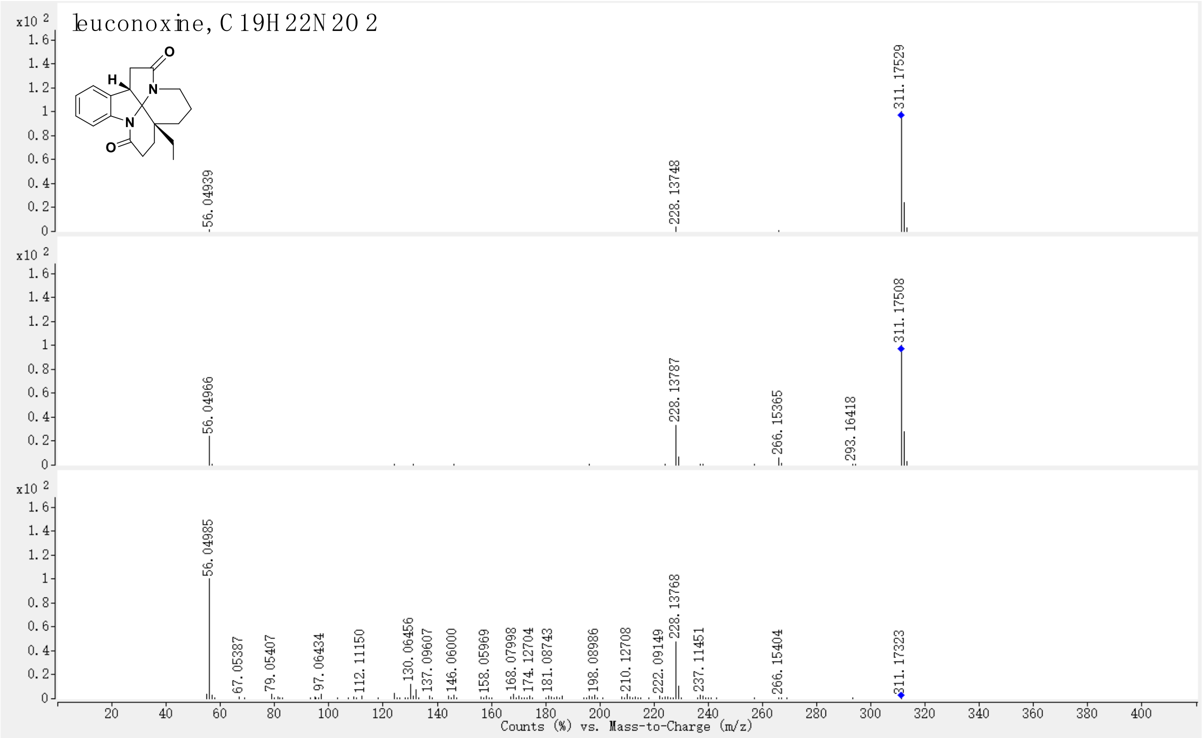

Figure S48. MCEs/MS^2 spectra of leuconoxine (**P205**).
GNPS spectrum ID: CCMSLIB00006710016

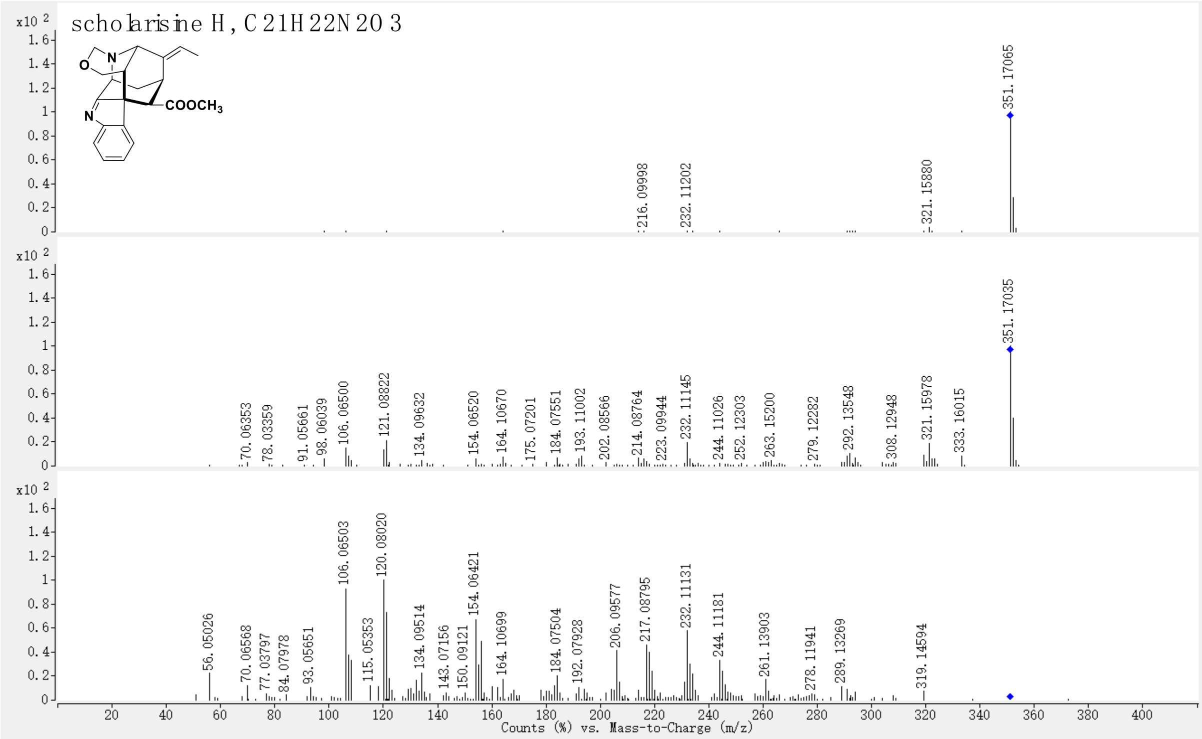

Figure S49. MCEs/MS^2 spectra of scholarisine H (**P213**).
GNPS spectrum ID: CCMSLIB00006710017

Characteristic fragmentation patterns of the subtype of MIAs

Alstolactine-type MIAs

Seven alstolactine-type alkaloid standards, including scholarisine M (**P220**), scholarisine L (**P221**), scholarisine E (**P176**), alstolactine A (**P171**), alstolactine B (**P172**), alstoniascholarine L (**P111**), and alstoniascholarine M (**P118**), were analyzed. The seven standards showed $[M+H]^+$ as the precursor ion in their MS^1 spectra (**P220**, **P221**, **P176**: *m/z* 385.1758 $[C_{21}H_{25}N_2O_5]^+$; **P171**, **P172**: *m/z* 371.1601 $[C_{20}H_{23}N_2O_5]^+$; **P111**, **P118**: *m/z* 357.1445 $[C_{19}H_{21}N_2O_5]^+$).

Alstolactine-type MIAs could be classified into two groups according to the existence of an F-lactone ring or not (group with F-lactone ring: **P171**, **P172**, **P111**, **P118**; group without F-lactone ring: **P220**, **P221**, **P176**). The structures within each group mainly differed in ring D. MS2 spectra of alstolactine-type MIAs were very simple, with high intensity of a series of ring D fragment ions (*m/z* < 200), and low intensity from *m/z* 400 to *m/z* 200. Hence, accurately identifying ring D moieties (*m/z* < 200) was the key to alstolactine-type MIAs recognition under MCEs/MS^2.

As shown in Figure S49, **P220** and **P221**, a pair of diastereoisomers, were taken as examples to illustrate common fragment patterns of alstolactine-type MIAs without F-lactone ring: (1) a DPI *m/z* 246.08 ($[C_{13}H_{12}NO_4]$+) corresponding to the indole ring skeleton and its complementary ring D DPI *m/z* 138.09 ($[C_8H_{12}NO]^+$) was generated through d_1-cleavage; (2) three concomitant DPIs *m/z* 110.06 ($[C_6H_8NO]^+$), *m/z* 94.06 ($[C_6H_8N]^+$) and *m/z* 82.06 ($[C_5H_8N]^+$) were created due to NLs of C_2H_4 (28.03 Da), C_2H_4O (44.03 Da) and C_2H_4+CO (56.02 Da) from *m/z* 138.09, respectively, which were consistent with a 2-methyl-1-oxiranyl group substituted on C-20 position of the ring D. Compared with **P220**, an isomer with identical molecular formula, **P176** differed in an OCH3 substituted on C-5 and demethylation on N-4 position, which resulted in a distinct indole ring skeleton DPI *m/z* 262.11 (d_1-cleavage) and four characteristic ring D DPIs *m/z* 124.08 (d_1-cleavage), *m/z* 96.04 (the NL of C2H4 from *m/z* 124.08), *m/z* 80.05 (the NL of C2H4O from *m/z* 124.08) and *m/z* 68.05 (the NL of C_2H_4+CO from *m/z* 124.08) in MCEs/MS^2 spectra of **P176**.

P171, **P172**, **P111,** and **P118** were alstolactine-type alkaloids with a F-lactone ring, and their fragment patterns were illustrated in Figure S50: (1) the DPI $[M+H-CH_2O_2]^+$ was created due to elimination of the F-lactone ring; (2) the ring D DPI $[M+H-215.06\ Da]^+$ was generated by d_2-cleavage; (3) a DPI $[M+H-215.06\ Da-H_2O]^+$ was generated through the elimination of the residual OH group caused by the incomplete elimination of the F-lactone ring; (4) two DPIs $[M+H-CH_2O_2-C_2H_4O]$+ and $[M+H-215.06\ Da-H_2O-C_2H_4O]$+ were created by the NL of C_2H_4O (44.03 Da) from $[M+H-CH_2O_2]$+ and $[M+H-215.06\ Da-H_2O]$+, respectively, which corresponded to a 2-hydroxyethyl group substituted on the C-20 position of ring D.

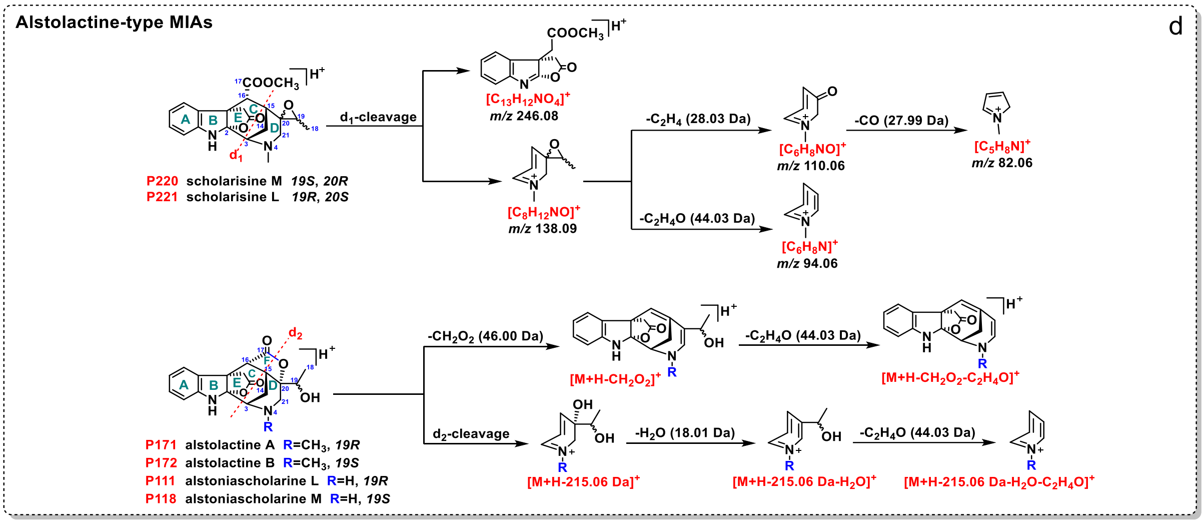

Figure S50. Characteristic fragment patterns of alstolactine-type MIAs.

Yohimbine-type MIAs

19*E*-geissoschizine (**P147**) and ajmalicine (**P157**), a pair of yohimbine-type MIA isomers, differed in the existence of ring E and showed $[M+H]^+$ as the precursor ion in MS^1 spectra (*m/z* 353.1860 $[C_{21}H_{24}N_2O_3]^+$).

As shown in Figure S51, the common fragment patterns of **P147** and **P157** were that a pair of complementary DPIs, m/z 210.11 ([C11H16NO3]+) and m/z 144.07 ([C10H10N]+), were generated by e-cleavage. Notably, for **P147**, a particular DPI *m/z* 251.15 ($[C_{17}H_{19}N_2]^+$) was generated through the complete elimination of the C-15 side chain due to the inexistence of ring E. Furthermore, in MS^2 spectra of **P147**, a pair of complementary DPIs *m/z* 108.08 ($[C_7H_{10}N]^+$) and *m/z* 144.07 ($[C_{10}H_{10}N]^+$) were created by e-cleavage on *m/z* 251.15. Hence, two special DPIs, m/z 251.15 and m/z 108.08, could be applied to distinguish the existence of ring E in yohimbine-type MIAs.

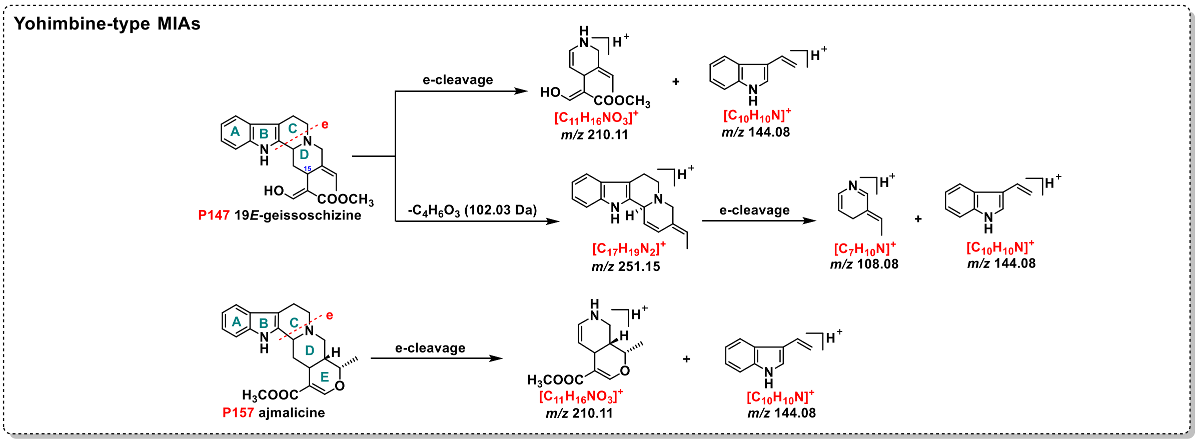

Figure S51. Characteristic fragment patterns of yohimbine-type MIAs.

Alstoscholarisine-type MIAs

Seven alstoscholarisine-type MIA standard compounds, including alstoscholarisine D (**P95**), alstoscholarisine E (**P141**), alstoscholarisine A (**P124**), alstoscholarisine C (**P179**), alstoscholarisine B (**P187**), alstoscholarisine H (**P112**), and alstoscholarisine I (**P135**), were analyzed. Alstoscholarisine-type MIAs could be classified into two groups (group 1: **P95**, **P141**, **P124**, **P179**, and **P187**; group 2: **P112** and **P135**) because of the rotation of a single bond between C-15 and C-16 during biosynthesis pathways. The seven alstoscholarisine-type MIAs showed $[M+H]^+$ as the precursor ion in MS^1 spectra (**P95**: *m/z* 341.1860 $[C_{20}H_{25}N_2O_3]^+$; **P141**, **P124**, **P112**: *m/z* 297.1961 $[C_{19}H_{25}N_2O]^+$; **P179**, **P187**, **P135**: *m/z* 355.2016 $[C_{21}H_{27}N_2O_3]^+$).

P95, **P141**, **P124**, **P179**, and **P187** differed in the substituent of C-16 (**P95**: COOH; **P141**, **P124**: H; **P179**, **P187**: $COOCH_3$) and absolute configuration of C-19 position (**P95**, **P141**, **P179**: *19S*; **P124**, **P187**: *19R*). As shown in Figure S52, common fragment patterns of **P95**, **P141**, **P124**, **P179**, and **P187** were generalized as: (1) two abundant DPIs *m/z* 44.05 ($[C_2H_6N]^+$) and *m/z* 96.08 ($[C_6H_{10}N]^+$) were generated by f_1-cleavage and rings C/E cleavage, respectively; (2) For **P95**, a particular DPI *m/z* 297.19 ($[C_{19}H_{25}N_2O]^+$) was generated through the NL of CO_2 (43.99 Da) corresponding to the COOH group on C-16 position; (3) For **P95**, **P141**, and **P124**, two common DPIs *m/z* 240.13 ($[C_{16}H_{18}NO]^+$) and *m/z* 210.12 ($[C_{15}H_{16}N]^+$) were caused by the ring D cleavage and ring E cleavage, respectively; (4) For **P179** and **P187**, a common DPI *m/z* 296.18 ($[M+H-{\bullet}COOCH_3]^{\bullet+}$) was generated by the radical loss of $\bullet COOCH_3$ corresponding to the $COOCH_3$ group on C-16 position.

P112 and **P135** differed in the substituent of the C-16 position (**P112**: H; **P135**: $COOCH_3$). As illustrated in Figure S52, the common fragment patterns of **P112** and **P135** were included: (1) a ring D DPI *m/z* 122.09 ($[C_8H_{12}N]^+$) was generated by f_2-cleavage; (2) two DPIs *m/z* 182.09 ($[C_{13}H_{12}N]^+$) and *m/z* 144.08 ($[C_{10}H_{10}N]^+$) corresponding to the indole ring skeleton were created by the ring D cleavage and f_2-cleavage after complete elimination of substituents on the C-16 position, respectively.

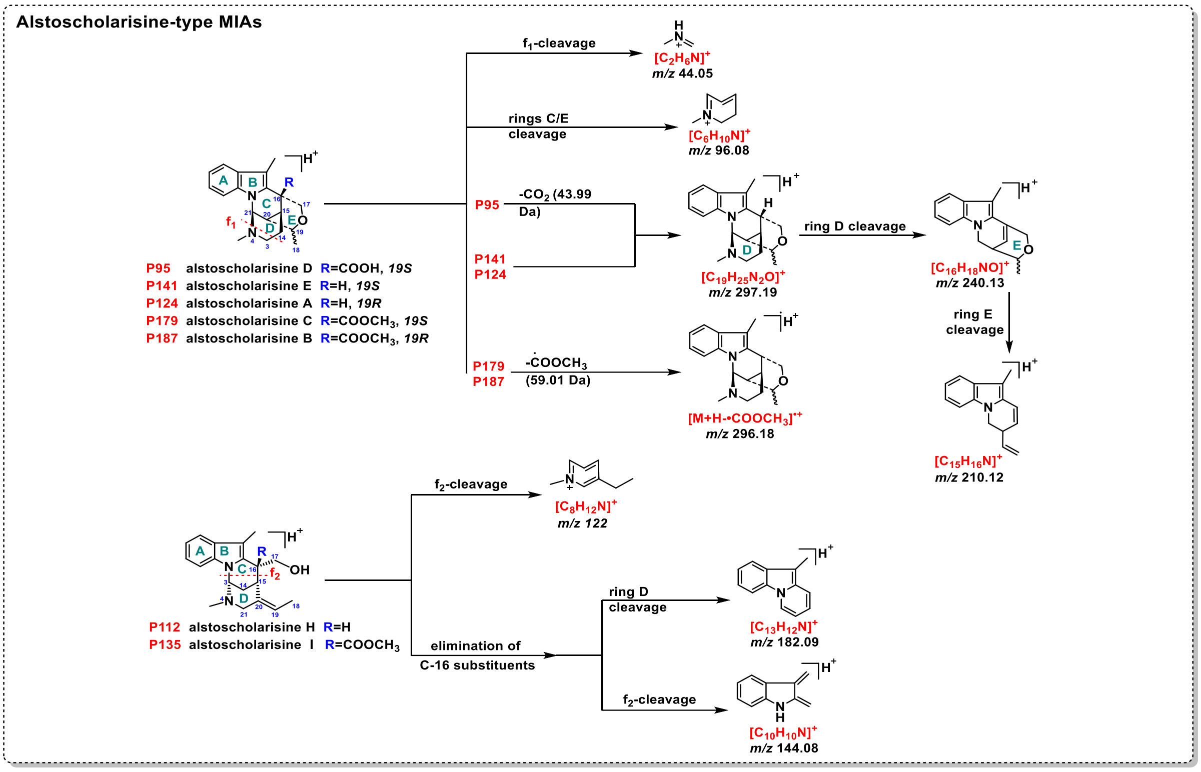

Figure S52. Characteristic fragmentation patterns of alstoscholarisine-type MIAs.

Scholarisine-type MIAs

Three scholarisine-type MIAs, including scholarisine I (**P67**), scholarisine J (**P10**), and scholarisine A (**P158**), differed in substituents of the N-4 and C-17 positions, showing $[M+H]^+$ as the precursor ion in their MS[1] spectra (**P67**: *m/z* 323.1754 $[C_{20}H_{23}N_2O_2]^+$; **P10**: *m/z* 325.1911 $[C_{20}H_{25}N_2O_2]^+$; **P158**: *m/z* 307.1441 $[C_{19}H_{19}N_2O_2]^+$).

The fragmentation behaviors of **P67**, **P10**, and **P158** were investigated, which were rarely reported previously. Although numerous and complex fragment ions were observed in MS^2 spectra of the three scholarisine-type MIAs, the common fragment patterns of **P67**, **P10**, and **P158** could be generalized as a weak protonated indole ring skeleton DPI (**P67**: *m/z* 200.07; **P10**: *m/z* 202.08; **P158**: *m/z* 198.05) and its abundant complementary DPI (**P67** and **P10**: *m/z* 124.00; **P158**: *m/z* 108.08) generated by g-cleavage (Figure S53). Due to the featureless fragment patterns, the identification of scholarisine-type MIAs in complex matrices should be rigorously compared with the standard compounds.

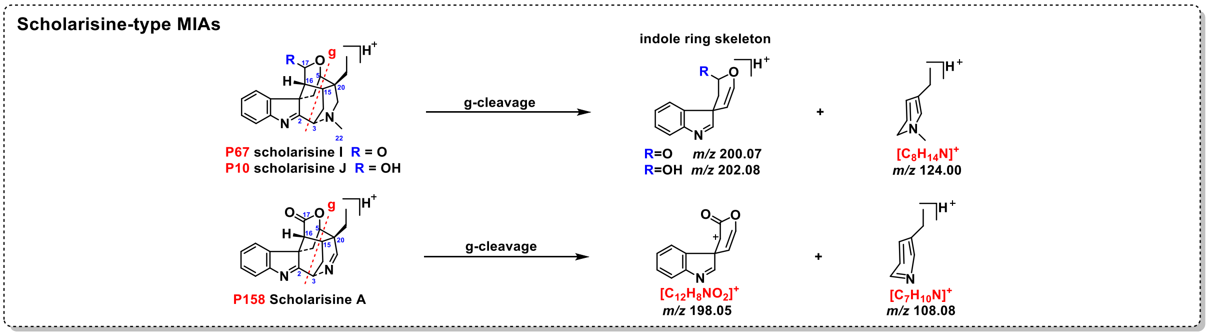

Figure S53. Characteristic fragmentation patterns of scholarisine-type MIAs.

Vallesiachotamine-type MIAs

Vallesiachotamine (**P225**) and isovallesiachotamine (**P226**), a pair of *cis*/*trans*-19,20-vallesiachotamine-type MIA isomers (**P225**: *trans*; **P226**: *cis*), showed $[M+H]^+$ as the precursor ion in their MS^1 spectra (*m/z* 351.1703 $[C_{21}H_{23}N_2O_3]^+$). Identical fragment ions, ion intensities, and ion ratios were observed in the MS^2 spectra of **P225** and **P226**. Therefore, **P226** was taken as an example to illustrate common fragment patterns of vallesiachotamine-type MIAs (Figure S54): (1) a DPI *m/z* 319.14 ($[M+H-CH_4O]^+$) was generated due to the incomplete elimination of the $COOCH_3$ group on the C-16 position; (2) the second DPI *m/z* 249.10 ($[M+H-CH_4O-C_4H_6O]^+$) was created by the complete elimination of the C-15 side chain on *m/z* 319.14; (3) two DPIs *m/z* 170.09 ($[C_{12}H_{12}N]^+$) and *m/z* 167.07 ($[C_9H_{11}O_3]^+$) were caused by h_1-cleavage and h_2-cleavage on $[M+H]^+$, respectively; (4) two DPIs *m/z* 107.05 ($[C_7H_7O]^+$) and *m/z* 79.05 ($[C_6H_7]^+$) were generated by the NL of $C_2H_4O_2$ (60.02 Da) and $C_2H_4O_2$+CO (88.01 Da) from *m/z* 167.07, respectively, which corresponded to the $COOCH_3$ group on the C-16 position and the CHO group on the C-20 position.

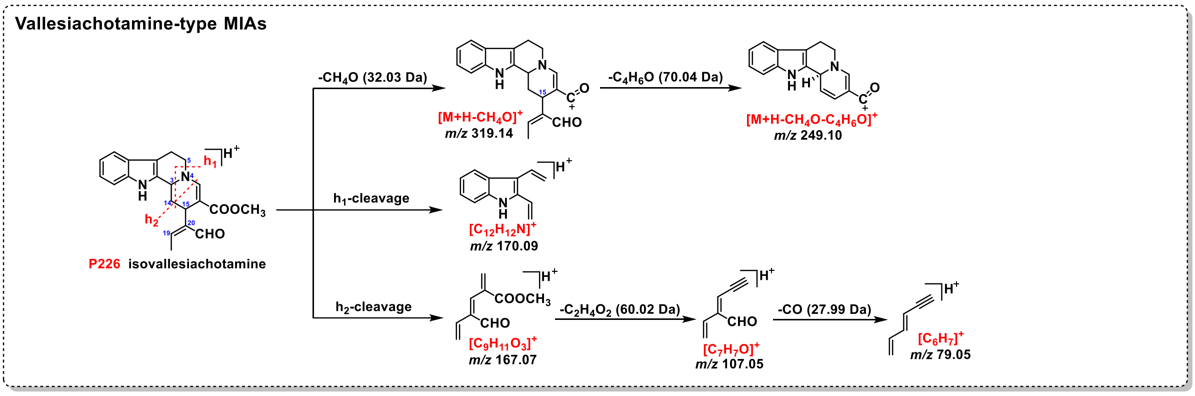

Figure S54. Characteristic fragmentation patterns of vallesiachotamine-type MIAs.

Alstoscholarine-type MIAs

Z-alstoscholarine (**P227**) and *E*-alstoscholarine (**P228**), a pair of *cis*/*trans*-19,20- isomers (**P227**: *cis*; **P228**: *trans*), were used to investigate fragment patterns of alstoscholarine-type MIAs. Both **P227** and **P228** showed $[M+H]^+$ as the precursor ion in their MS^1 spectra (**P227** and **P228**: *m/z* 361.1547 $[C_{22}H_{21}N_2O_3]^+$).

Identical fragment patterns were observed in MS^2 spectra of **P227** and **P228** due to insignificant structural differences between them. As shown in Figure S55, **P227** was taken as an example to illustrate common fragment patterns of alstoscholarine-type MIAs: (1) a predominant DPI *m/z* 301.13 ($[M+H-C_2H_4O_2]^+$) was generated due to the complete elimination of the $COOCH_3$ group on the C-16 position; (2) a pair of complementary DPIs *m/z* 172.07 ($[C_{11}H_{10}NO]^+$) and *m/z* 188.07 ($[C_{11}H_{10}NO_2]^+$) were generated through i-cleavage on *m/z* 301.13; (3) two DPIs *m/z* 273.13 ($[M+H-C_2H_4O_2-CO]^+$) and *m/z* 144.08 ($[C_{10}H_{10}N]^+$) were generated by the elimination of the CHO group on the C-22 position; (4) the DPI *m/z* 130.06 ($[C_9H_8N]^+$) which corresponded to the indole ring skeleton was created by the NL of $C_2H_2O_2$ (58.00 Da) from *m/z* 188.07 or i-cleavage on *m/z* 273.13.

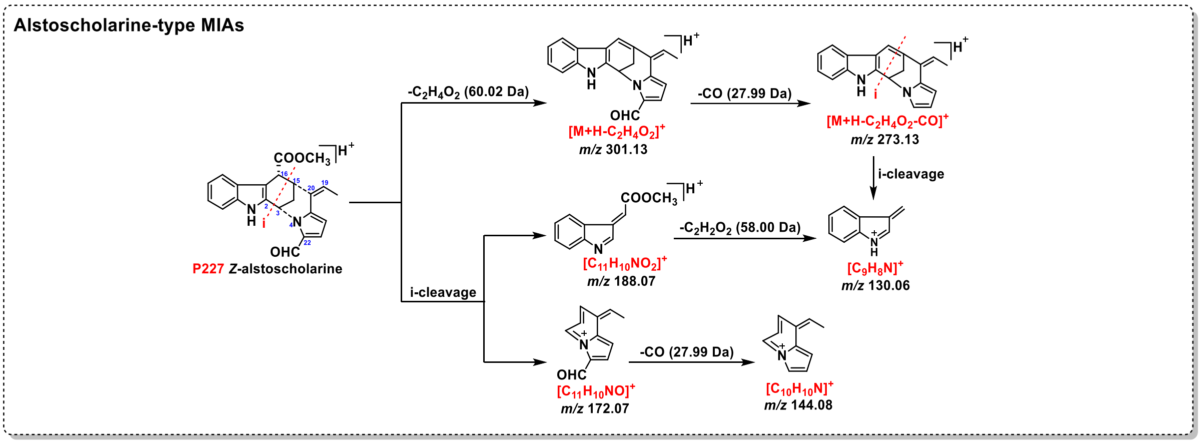

Figure S55. Characteristic fragmentation patterns of alstoscholarine-type MIAs.

Unclassified MIAs

Five unclassified MIAs, including strictosamide (**P188**), alstoscholarisine F (**P159**), (+)-vincadifformine (**P181**), leuconoxine (**P205**), and scholarisine H (**P213**), were analyzed. The five unclassified MIAs showed $[M+H]^+$ as the precursor ion in MS^1 spectra (**P188**: *m/z* 499.2074 $[C_{26}H_{31}N_2O_8]^+$; **P159**: *m/z* 293.1284 $[C_{18}H_{17}N_2O_2]^+$; **P181**: *m/z* 339.2067 $[C_{21}H_{27}N_2O_2]^+$; **P205**: *m/z* 311.1754 $[C_{19}H_{23}N_2O_2]^+$; **P213**: *m/z* 351.1703 $[C_{21}H_{23}N_2O_3]^+$).

The fragmentation behaviors of **P188**, **P159**, **P181**, **P205** and **P213** were investigated (Figure S56). Fragment patterns of **P188** (strictosamide) were included: (1) the DPI *m/z* 337.15 was generated by eliminating Glc (162.05 Da); (2) a pair of DPIs *m/z* 267.11 and 171.09 was caused due to j_1-cleavage and j_2-cleavage, respectively. For **P159**, two DPIs *m/z* 278.10 ($[M+H-\bullet CH_3]^{\bullet+}$) and 263.08 ($[M+H-\bullet CH_3-\bullet CH_3]^+$) were produced through the elimination of two CH_3 groups on the C-7 and C-19 positions. For **P181**, fragment patterns were generalized as: (1) the abundant DPI *m/z* 307.18 was generated by eliminating CH_4O (32.03 Da); (2) two DPIs *m/z* 138.12 and 124.11 were caused due to the k_1-cleavage and k_2-cleavage, respectively. For **P205**, two abundant DPIs *m/z* 228.13 and *m/z* 56.04 were generated through l_1-cleavage and l_2-cleavage, respectively. For **P213**, DPIs contained *m/z* 164.10 (by m-cleavage) and 134.09 (by neutral loss of CH_2O after m-cleavage).

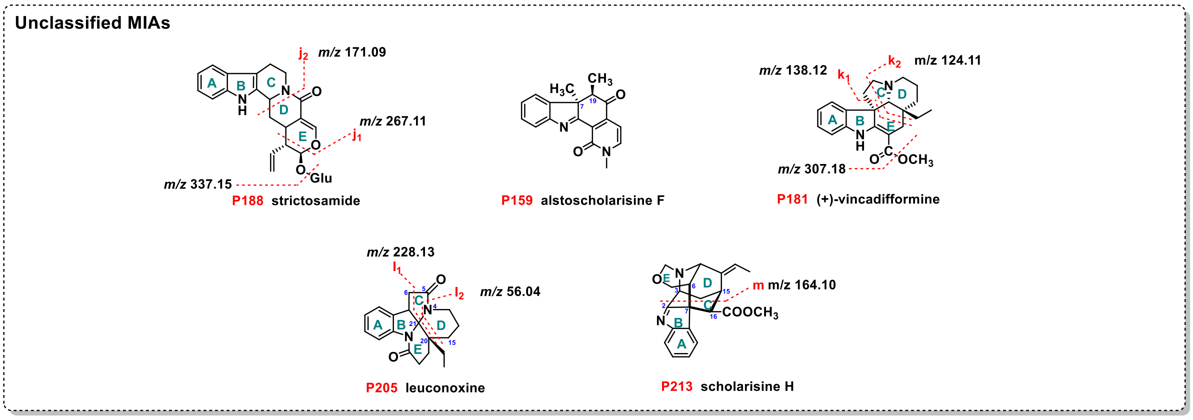

Figure S56. Characteristic fragmentation patterns of unclassified MIAs.

Common neutral losses and radical losses of MIAs

Table S2. Common neutral losses and radical losses of MIAs.

Common NLs/RLs		
Molecular weight (Da)	NLs/RLs	Generated by MIAs (subunits)
15.02	$\bullet CH_3$	R-OCH_3; N-CH_3
15.99	O	MIAs *N*-oxide
17.00	•OH	MIAs *N*-oxide
18.01	H_2O	R-COOH; R-OH; MIAs *N*-oxide
26.01	C_2H_2	R-CH=$CHCH_3$; R-CH=CH_2
27.99	CO	R-$COOCH_3$; R-CHO; R-CO-R’; R-COO-R’
28.03	C_2H_4	R-CH_2CH_3; R-CH=$CHCH_3$; R-CH=CH_2
30.01	CH_2O	R-CH_2OH; R-OCH_3; R-CH_2-O-R’
32.02	CH_4O	R-$COOCH_3$; R-OCH_3
43.98	CO_2	R-COOH
44.02	C_2H_4O	R-CHOH-CH_3; R-CH_2CH_2O-R’
46.00	CH_2O_2	R-COO-R’; R-CHOH-O-R’; R-COOH
58.00	$C_2H_2O_2$	R-$COOCH_3$
59.01	$\bullet C_2H_3O_2$	R-$COOCH_3$
60.02	$C_2H_4O_2$	R-$COOCH_3$

MZmine 2 processing of LC-MS2 datasets

MSconvert was employed to convert the original ".d" data to ".mZML" format that was submitted to MZmine 2 by referring to the previous processing flow [32, 33], and was improved in the present study to screen MIA features in ALAS (Figure S57). In brief, (1) the dataset of ".mZML" format was firstly imported into MZmine 2; (2) in the module of feature detection, filtering thresholds of MS^1 and MS^2 were set at 1E3 and 1E1, respectively; (3) ADAP chromatography builder module was used to create a feature list; (4) Chromatogram deconvolution module was used to cover most of the isomeric MIAs using the local minimum search function in Algorithm; (5) Isotopes module was used to apply the peaks grouper function to elimitate isotope mass information; (6) Alignment module was used to apply the join aligner function to align the peak lists from each sample in one aligned peak list; (7) Filtering module used the feature list row filter function to filter MS^2 features; (8) Gap filling enabled the retrieval of the intensity of a peak in all the samples; (9) the most important step in this study is filtering MS2 features of non-MIAs; molecular weight was set in the range of 180 Da-500 Da, elemental composition was set at C 0-50, H 0-80, O 0-30, N 0-2, and DBE was set at 5-20; charge of precursor ion was set at 1 and molecular formula with ppm < 2 was generated accurately; finally, the RT-consistent MS^2 features were manually compared to filter the fake features generated by the in-source collision induced dissociation (IS-CID); (10) ".csv" and ".mgf" files were exported to GNPS for FBMN analysis [34].

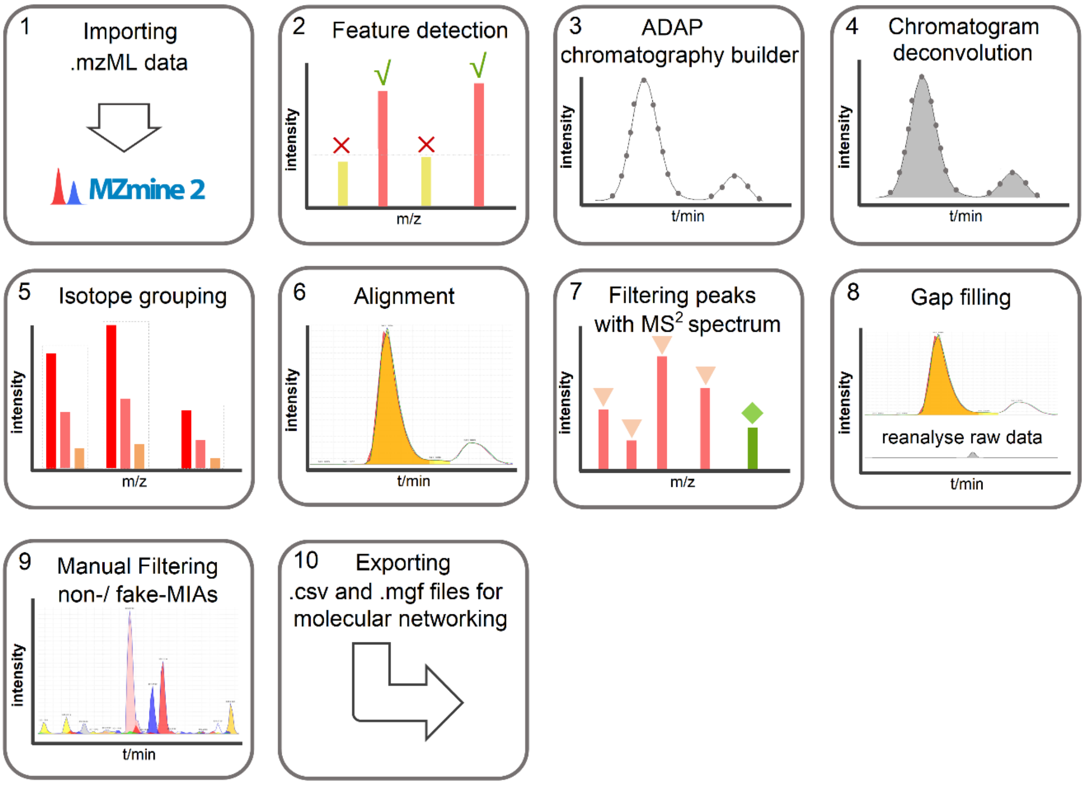

Figure S57. The scheme of the LC-MS^2 dataset processing steps with MZmine 2.

FBMN construction of ALAS and automatic annotation of MIAs

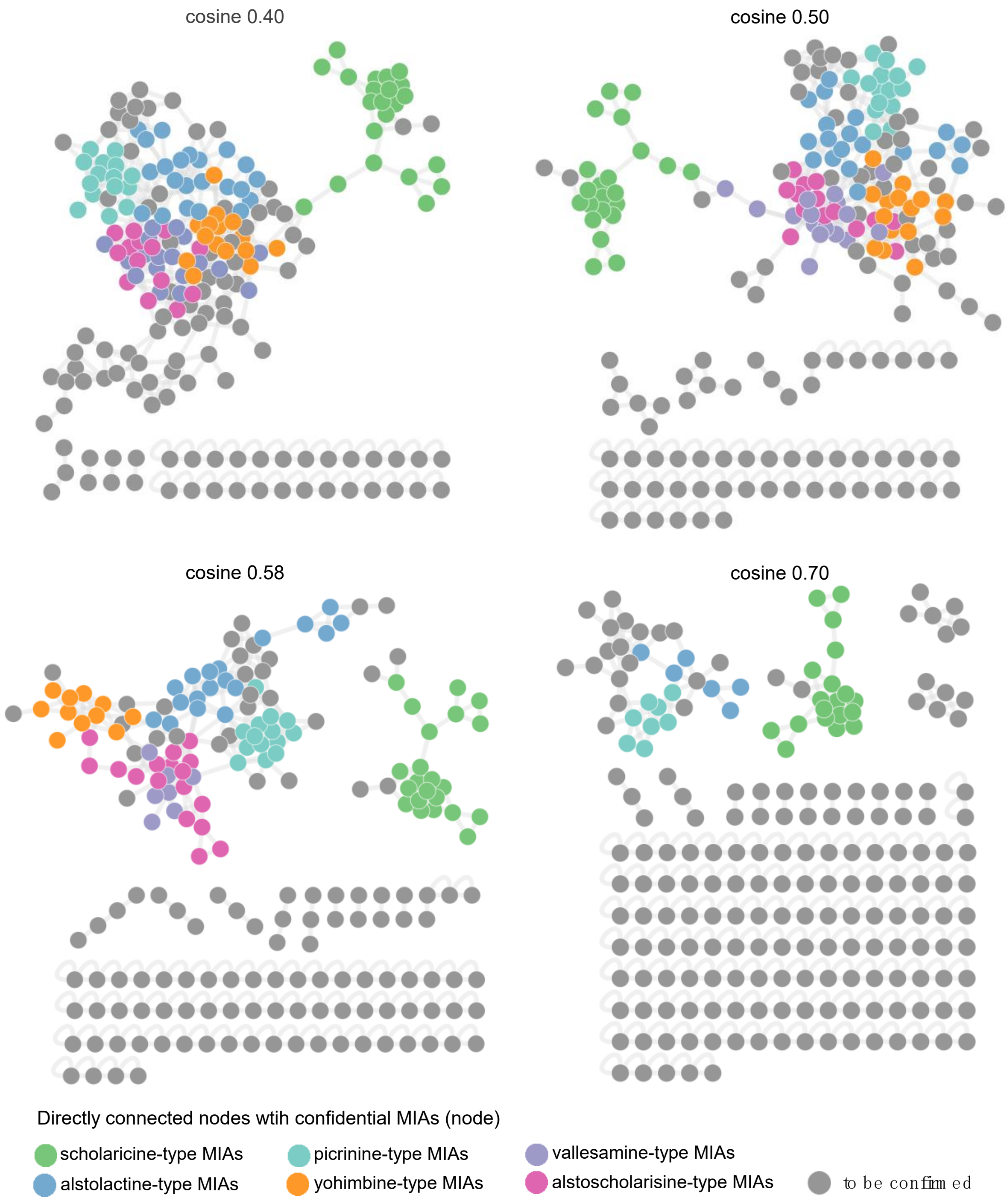

Figure S58. Construction of feature-based molecular networking (FBMN) of MIAs in ALAS with different cosine values.

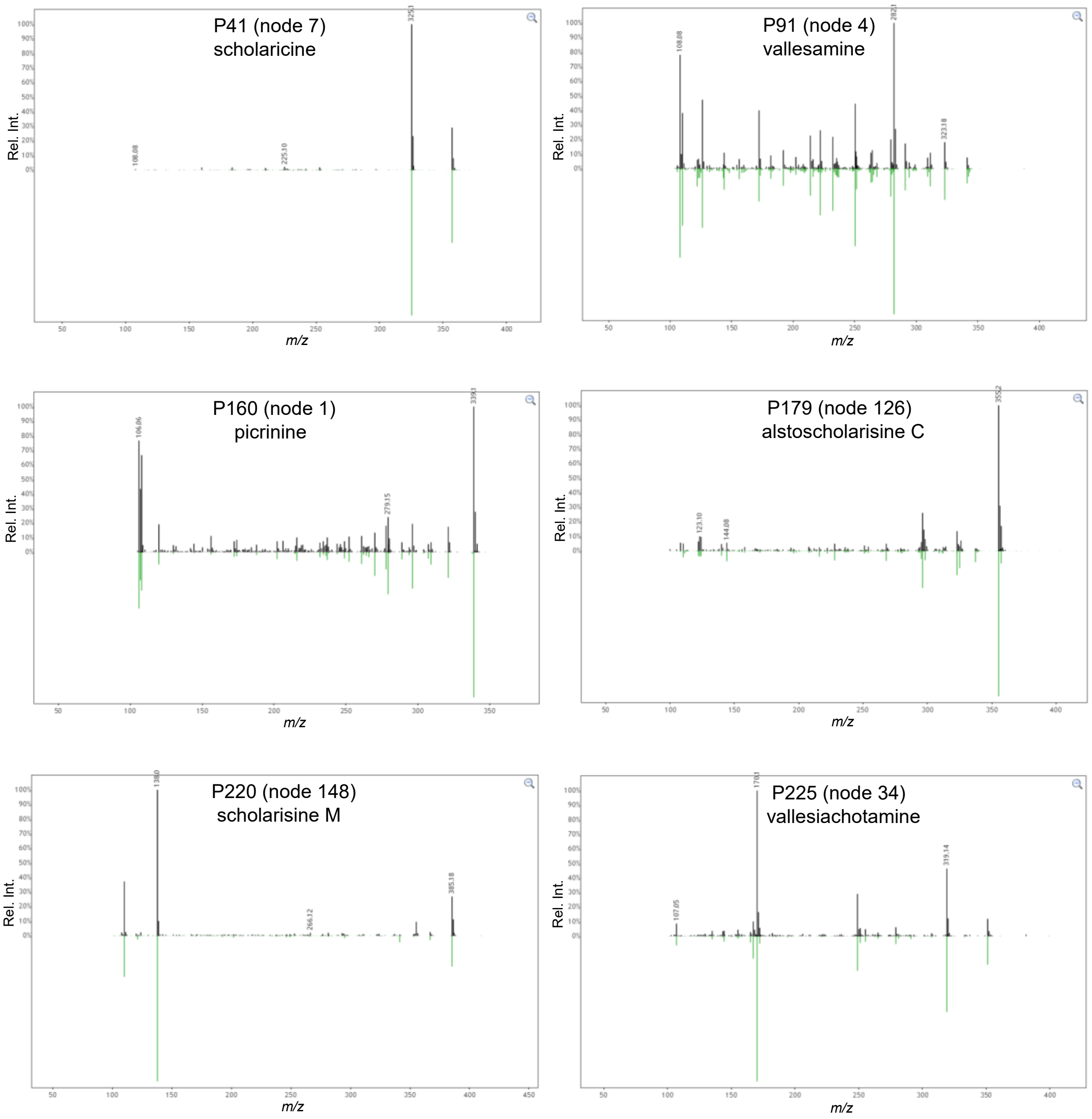

Figure S59. Representative nodes automatically matched by reference MIAs.

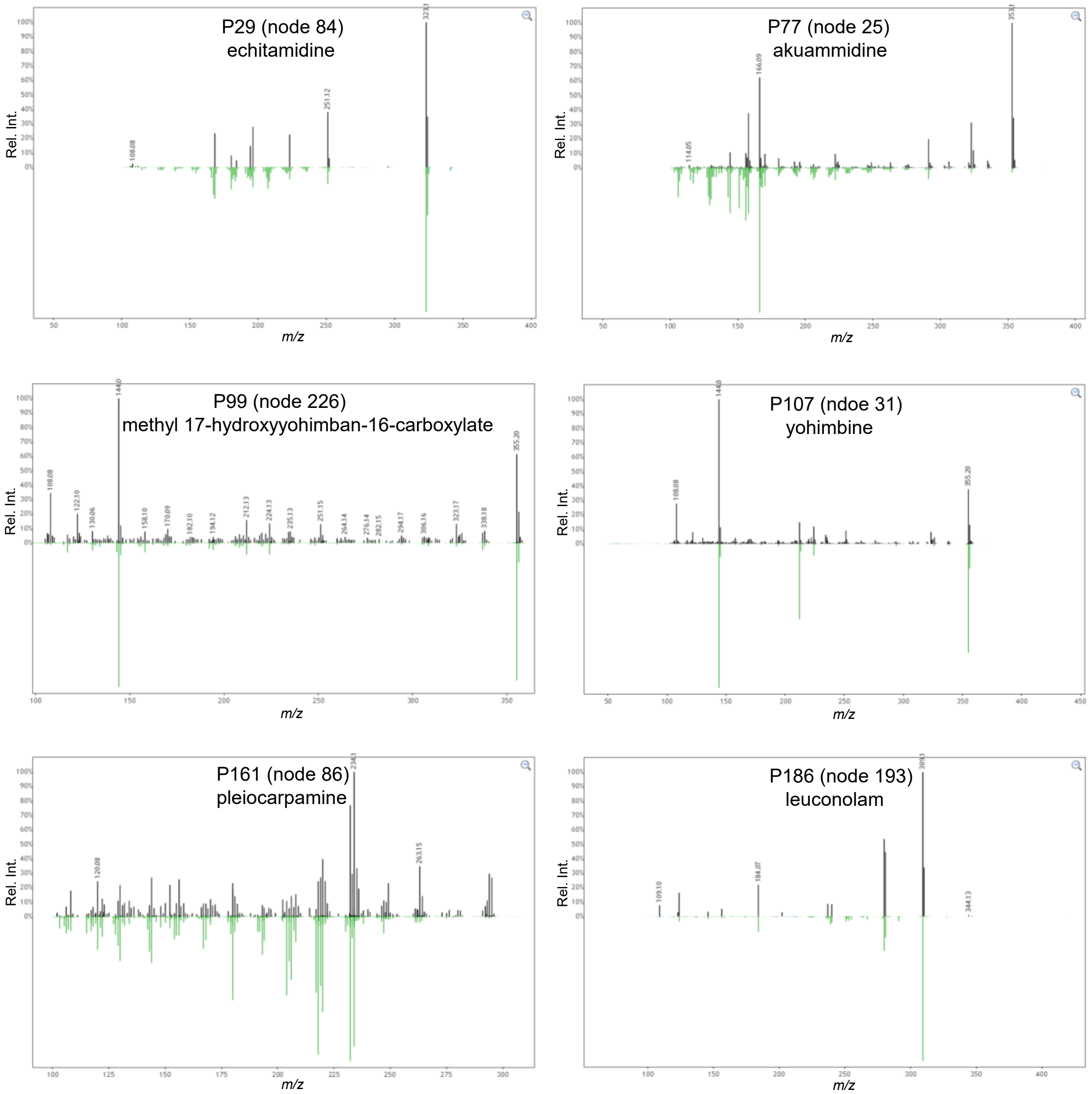

Figure S60. Representative nodes automatically matched in GNPS Libraries.

Hypothetical biogenetic pathways of *Alstonia* MIAs

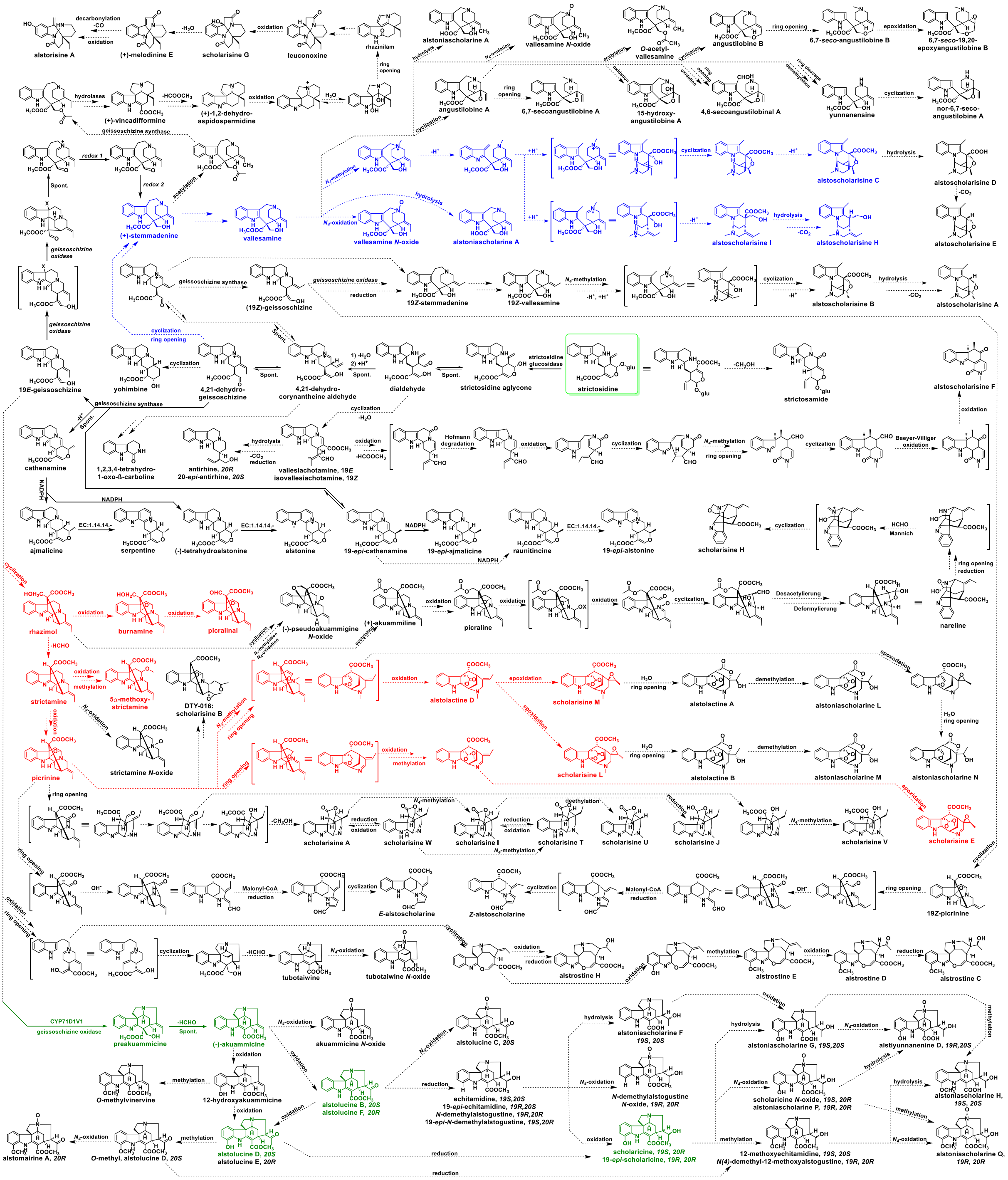

Figure S61. Hypothetical biogenetic pathways of *Alstonia* MIAs.

Construction of in silico database of *Alstonia* MIAs

skeletons | **common substituents** | ***in silico* database**

Scholaricine-type MIAs

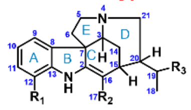

C-12	C-16	C-19	N-4
H	CHO	alkene(C19,C20)	no substituent
OH	COOH	O	O
OCH_3	$COOCH_3$	OH	

71 MIAs

Picrinine-type MIAs

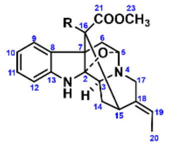

C-16		C-10	C-11	N-1
$COOCH_3$	H	H	H	H
$COOCH_3$	CH_2OH	OH	OH	CH_3
$COOCH_3$	CHO	OCH_3	OCH_3	

56 MIAs

Vallesamine-type MIAs

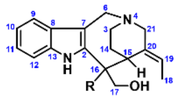

C-16		C-6	N-4
COOH	CH_2OH	no substituent	no substituent
$COOCH_3$	CH_2OH	O	O
COOH	CH_2OCOCH_3		
$COOCH_3$	CH_2OCOCH_3		

17 MIAs

Alstolactine-type MIAs

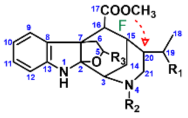

ring F	C-5	C-19	N-4
No	alkene(C5,C6)	expodation(C19,C20)	no substituent
Yes	O	alkene(C19,C20)	H
	OH		CH_3
	OCH_3		O

54 MIAs

Figure S62. Construction of in silico database of *Alstonia* MIAs.

Systematically annotated MIAs in ALAS by MCEs/MS^2-FBMN/BPs

Table S3. Investigation of MIAs in ALAS by UHPLC-ESI-QTOF-MS^E.

Peak No.	FBMN node	RT (min)	Ion type	Precursor ion (calc.)	Precursor ion (exp.)	Δppm	DBE	Formula	Fragment ions	Annotation	Level ※	Average area (%)
							**Scholaricine-type MIAs**					
**P8**	1161	8.12	$[M+H]^+$	355.16523	355.16520	0.10	11	$C_{20}H_{22}N_2O_4$	339.17, 311.18, 267.11, 239.12, 146.06	dehydro-alstoniascholarine H	L2	<0.1
**P9**	214	8.20	$[M+H]^+$	357.18088	357.18069	0.54	10	$C_{20}H_{24}N_2O_4$	339.17, 311.18, 267.11, 239.12, 212.10	alstoniascholarine H isomer 1	L2	<0.1
**P12**	39	8.51	$[M+H]^+$	357.18088	357.18058	0.85	10	$C_{20}H_{24}N_2O_4$	339.17, 311.18, 267.11, 239.12, 212.10, 184.08, 146.06	alstoniascholarine H★ 12-methoxy-alstoniascholarine F	L1	0.69
**P14**	777	8.68	$[M+H]^+$	353.18597	353.18595	0.05	11	$C_{21}H_{24}N_2O_3$	335.17, 291.18, 222.13, 144.08, 108.08	scholaricine-like	L3	<0.1
**P15**	21	8.90	$[M+H]^+$	343.16523	343.16513	0.30	10	$C_{19}H_{22}N_2O_4$	325.15, 297.16, 253.10, 225.10, 210.09, 196.08, 184.08, 146.06	alstoniascholarine G 12-hydroxy-alstoniascholarine F scholaricine acid	L2	0.85
**P16**	1327	9.17	$[M+H]^+$	369.18088	369.18081	0.20	11	$C_{22}H_{24}N_2O_4$	351.17, 339.17	scholaricine-like	L3	<0.1
**P21**	376	9.95	$[M+H]^+$	373.17580	373.17566	0.37	10	$C_{20}H_{24}N_2O_5$	355.16, 312.12, 295.14, 280.10, 252.10, 239.09, 160.07	alstoniascholarine H oxide	L2	<0.1
**P22**	268	10.24	$[M+H]^+$	357.18088	357.18072	0.46	10	$C_{20}H_{24}N_2O_4$	339.17, 204.10, 172.07, 160.08, 152.07, 146.06, 132.08, 120.04, 108.08	alstoniascholarine H isomer 2	L2	<0.1
**P23**	326	10.41	$[M+H]^+$	357.18088	357.18053	0.99	10	$C_{20}H_{24}N_2O_4$	339.17, 325.15, 198.11, 160.08, 146.06, 132.08, 108.08	alstoniascholarine H isomer 3	L2	<0.1
**P26**	59	10.95	$[M+H]^+$	281.16484	281.16466	0.64	10	$C_{18}H_{20}N_2O$	264.14, 252.14, 238.12, 210.09, 174.09, 160.08, 134.10, 122.10, 108.08	(de)CH_2O_2-alstoniascholarine F	L2	<0.1
**P28**	10824	11.10	$[M+H]^+$	357.18088	357.18054	0.97	10	$C_{20}H_{24}N_2O_4$	325.16, 268.16, 224.10, 160.08, 108.08	alstoniascholarine H isomer 4	L2	<0.1
**P29**	84	11.11	$[M+H]^+$	341.18597	341.18577	0.59	10	$C_{20}H_{24}N_2O_3$	323.17, 251.12, 223.12, 208.08, 196.10, 168.08	12-methyl-alstoniascholarine F	L2	<0.1
**P31**	10846	11.41	$[M+H]^+$	355.16523	355.16515	0.24	11	$C_{20}H_{22}N_2O_4$	325.16, 311.18, 224.11, 185.08, 160.08, 146.06,	dehydro-alstoniascholarine H	L2	<0.1

Peak No.	FBMN node	RT (min)	Ion type	Precursor ion (calc.)	Precursor ion (exp.)	Δppm	DBE	Formula	Fragment ions	Annotation	Level ※	Average area (%)
									122.06, 108.08	isomer 1		
**P33**	448	11.57	$[M+H]^+$	355.16523	355.16516	0.21	11	$C_{20}H_{22}N_2O_4$	325.15, 239.13, 224.11, 185.08, 160.08, 144.08, 122.10, 108.08	dehydro-alstoniascholarine H	L2	<0.1
**P35**	299	11.86	$[M+H]^+$	355.16523	355.16516	0.21	11	$C_{20}H_{22}N_2O_4$	326.14, 325.16, 266.12, 239.13, 224.11, 214.11, 185.08, 160.08, 141.05, 124.07, 122.10	dehydro-alstoniascholarine H isomer 2	L2	<0.1
**P36**	93	11.89	$[M+H]^+$	327.17032	327.17031	0.03	10	$C_{19}H_{22}N_2O_3$	309.16, 237.10, 209.11, 168.01	alstoniascholarine F★	L1	<0.1
**P37**	19	11.97	$[M+H]^+$	371.19653	371.19637	0.44	10	$C_{21}H_{26}N_2O_4$	339.17, 311.17, 267.11, 250.12, 239.12, 236.07, 224.10, 212.09, 200.09, 196.07, 183.07, 146.06, 108.08	scholarine▲ 12-methoxy-echitamidine	L2	1.17
**P38**	11	12.13	$[M+H]^+$	371.19653	371.19642	0.31	10	$C_{21}H_{26}N_2O_4$	311.17, 285.12, 267.11, 250.12, 239.12, 224.10, 212.09, 200.09, 196.08, 184.08, 166.07, 146.06, 108.08	12-methoxy-echitamidine isomer	L2	1.5
**P39**	132	12.25	$[M+H]^+$	369.18088	369.18072	0.44	11	$C_{21}H_{24}N_2O_4$	339.17, 337.15, 267.11, 239.12, 212.09, 210.05, 184.08, 160.07, 156.08, 146.06	methyl-alstolucine D	L2	<0.1
**P41**	7	12.48	$[M+H]^+$	357.18088	357.18069	0.54	10	$C_{20}H_{24}N_2O_4$	325.15, 279.15, 253.10, 242.08, 225.10, 210.05, 196.08, 184.07, 166.06, 160.08, 142.06, 132.08, 108.08	scholaricine★	L1	3.87
**P42**	52	12.65	$[M+H]^+$	355.16523	355.16519	0.12	11	$C_{20}H_{22}N_2O_4$	323.14, 285.12, 253.10, 225.10, 210.06, 196.07, 183.07, 182.06, 146.06	alstolucine D▲ 19-oxo-scholaricine	L2	<0.1
**P43**	147	12.72	$[M+H]^+$	371.19653	371.19636	0.47	10	$C_{21}H_{26}N_2O_4$	339.17, 311.18, 267.11, 239.12, 224.09, 212.10, 210.09, 184.08, 146.06, 140.11, 108.08	12-methoxy-echitamidine isomer	L2	<0.1
**P44**	70	12.74	$[M+H]^+$	355.20162	355.20151	0.31	10	$C_{21}H_{26}N_2O_3$	325.16, 323.18, 307.14, 297.16, 279.15, 253.10, 225.10, 208.11, 194.10, 172.08, 168.08, 108.08	alstolucine D isomer 1	L2	<0.1

Peak No.	FBMN node	RT (min)	Ion type	Precursor ion (calc.)	Precursor ion (exp.)	Δppm	DBE	Formula	Fragment ions	Annotation	Level ※	Average area (%)
**P45**	10	12.84	$[M+H]^+$	357.18088	357.18055	0.94	10	$C_{20}H_{24}N_2O_4$	325.15, 307.14, 279.15, 253.10, 225.10, 210.05, 196.08, 183.07, 172.08, 108.08	19-*epi*-scholaricine★ alstoniascholarine O	L1	1.54
**P51**	66	13.42	$[M+H]^+$	357.18088	357.18066	0.63	10	$C_{20}H_{24}N_2O_4$	325.15, 307.14, 279.15, 253.10, 225.10, 210.05, 196.08, 183.07, 172.08, 108.08	scholaricine isomer	L2	<0.1
**P52**	121	13.49	$[M+H]^+$	369.18088	369.18078	0.28	11	$C_{21}H_{24}N_2O_4$	351.17, 337.15, 319.14, 309.16, 265.13, 208.10, 196.10, 184.08, 174.09, 166.09, 158.09, 152.07, 144.08, 124.08, 108.08	hydroxy-akuammidine	L2	<0.1
**P54**	48	13.86	$[M+H]^+$	355.16523	355.16521	0.07	11	$C_{20}H_{22}N_2O_4$	323.14, 295.14, 285.12, 253.10, 242.08, 230.08, 225.10, 210.05, 198.09, 146.06,	alstolucine D isomer	L2	<0.1
**P55**	45	13.91	$[M+H]^+$	341.18597	341.18575	0.64	10	$C_{20}H_{24}N_2O_3$	309.16, 291.15, 237.10, 209.11, 194.06	echitamidine	L2	<0.1
**P61**	51	14.39	$[M+H]^+$	373.17580	373.17568	0.32	10	$C_{20}H_{24}N_2O_5$	355.17, 341.15, 251.12, 241.07, 209.05, 122.10, 100.08	scholaricine *N*-oxide★	L1	<0.1
**P66**	85	14.97	$[M+H]^+$	373.17580	373.17571	0.24	10	$C_{20}H_{24}N_2O_5$	341.14, 311.14, 296.15, 281.13, 257.13, 251.12, 122.10, 108.08	alstoniascholarine P★ 19-*epi*-scholaricine *N*-oxide	L1	<0.1
**P70**	no	15.19	$[M+H]^+$	357.18088	357.18066	0.63	10	$C_{20}H_{24}N_2O_4$	339.17, 267.11, 239.12, 224.09, 198.09, 55.05	alstoniascholarine H☆	L1	<0.1
**P75**	55	15.95	$[M+H]^+$	371.19653	371.19639	0.39	10	$C_{21}H_{26}N_2O_4$	353.19, 339.17, 321.16, 297.16, 293.16, 280.13, 265.13, 250.12, 220.11, 208.11, 194.10, 144.08, 122.10, 120.08, 108.08	CH_2O_2-tubotaiwine	L2	<0.1
**P77**	25	16.21	$[M+H]^+$	353.18597	353.18588	0.25	11	$C_{21}H_{24}N_2O_3$	335.18, 323.17, 291.15, 263.15, 222.13, 166.09, 158.10, 156.08, 144.08, 108.08	akuammidine▲	L2	1.15
**P79**	390	16.51	$[M+H]^+$	341.18597	341.18588	0.26	10	$C_{20}H_{24}N_2O_3$	323.18, 309.16, 297.20, 220.11, 194.06, 188.07, 108.08	deoxy-alstoniascholarine H	L2	<0.1
**P80**	246	16.56	$[M+H]^+$	353.18597	353.18589	0.22	11	$C_{21}H_{24}N_2O_3$	323.18, 166.09, 144.08,	akuammidine isomer	L2	<0.1

Peak No.	FBMN node	RT (min)	Ion type	Precursor ion (calc.)	Precursor ion (exp.)	Δppm	DBE	Formula	Fragment ions	Annotation	Level ※	Average area (%)
									122.10			
**P81**	57	16.58	$[M+H]^+$	355.20162	355.20150	0.34	10	$C_{21}H_{26}N_2O_3$	323.18, 251.12, 223.12, 208.08, 196.10	19-methoxy-tubotaiwine	L2	<0.1
**P84**	67	16.92	$[M+H]^+$	355.20162	355.20141	0.59	10	$C_{21}H_{26}N_2O_3$	323.18, 295.18, 251.12, 223.12, 208.08, 196.10, 180.08, 168.08, 139.13, 122.06, 110.10	19-methoxy-tubotaiwine isomer	L2	0.82
**P94**	33	18.33	$[M+H]^+$	341.18597	341.18573	0.70	10	$C_{20}H_{24}N_2O_3$	309.16, 291.15, 237.10, 209.11, 194.06	echitamidine isomer	L2	1.57
**P97**	382	18.93	$[M+H]^+$	351.17032	351.17015	0.48	12	$C_{21}H_{22}N_2O_3$	321.16, 291.15, 148.11	dehydrated hydroxy-akuammidine	L2	<0.1
**P101**	61	19.20	$[M+H]^+$	339.17032	339.17013	0.56	11	$C_{20}H_{22}N_2O_3$	307.14, 279.15, 254.12, 240.14, 222.13, 160.08, 144.08, 122.10, 108.08	O-akuammicine	L2	0.84
**P109**	222	20.01	$[M+H]^+$	357.18088	357.18077	0.32	10	$C_{20}H_{24}N_2O_4$	339.17, 325.15, 235.12, 108.08	*N*-demethylalstogustine *N*-oxide★	L1	<0.1
**P132**	87	22.46	$[M+H]^+$	371.19653	371.19646	0.20	10	$C_{21}H_{26}N_2O_4$	339.17, 311.18, 267.11, 224.07, 210.09, 198.09, 144.08, 139.14, 122.10, 122.09	12-hydroxy-19-methoxy-tubotaiwine	L2	<0.1
**P134**	140	22.87	$[M+H]^+$	371.19653	371.19637	0.44	10	$C_{21}H_{26}N_2O_4$	353.18, 229.17, 321.16, 299.14, 256.10, 224.07, 160.07, 122.10	12-hydroxy-19-methoxy-tubotaiwine	L2	<0.1
**P140**	118	23.23	$[M+H]^+$	337.19105	337.19099	0.19	11	$C_{21}H_{24}N_2O_2$	321.16, 305.16, 246.13, 234.12, 220.11, 196.10, 183.09, 108.08,	methyl-akuammicine	L2	<0.1
**P143**	no	23.49	$[M+H]^+$	387.19145	387.19129	0.41	10	$C_{21}H_{26}N_2O_5$	355.16, 265.13, 94.06, 82.06	alstoniascholarine Q☆	L1	<0.1
**P148**	69	23.86	$[M+H]^+$	323.17540	323.17525	0.48	11	$C_{20}H_{22}N_2O_2$	291.15, 263.15, 234.12, 220.11, 182.09, 169.08, 156.08, 144.08, 130.07, 122.10, 108.08	akuammicine▲	L2	<0.1
**P162**	18	24.62	$[M+H]^+$	325.19105	325.19087	0.57	10	$C_{20}H_{24}N_2O_2$	293.16, 265.13/ 265.17, 236.14, 222.13, 208.11, 194.10, 180.08, 168.08	tubotaiwine★	L1	<0.1
**P174**	3	25.25	$[M+H]^+$	385.21218	385.21217	0.04	10	$C_{22}H_{28}N_2O_4$	353.19, 321.16, 303.15, 293.16, 208.11, 122.10, 120.08, 108.08	12-methoxy-19-methoxy-tubotaiwine	L2	6.13
**P184**	169	26.13	$[M+H]^+$	341.18597	341.18591	0.17	10	$C_{20}H_{24}N_2O_3$	309.16, 291.15, 264.16, 229.11, 221.11, 207.09, 110.10, 108.08	tubotaiwine *N*-oxide★	L1	<0.1

Peak No.	FBMN node	RT (min)	Ion type	Precursor ion (calc.)	Precursor ion (exp.)	*Δ*ppm	DBE	Formula	Fragment ions	Annotation	Level ※	Average area (%)
**P201**	8	26.92	$[M+H]^+$	385.21218	385.21211	0.19	10	$C_{22}H_{28}N_2O_4$	353.19, 321.16, 303.15, 293.16, 208.11, 122.10, 120.08, 108.08	12-methoxy-19-methoxy-tubotaiwine isomer	L2	3.09
						**Picrinine-type MIAs**						
**P18**	114	9.59	$[M+H]^+$	355.16523	355.16511	0.35	11	$C_{20}H_{22}N_2O_4$	337.16, 325.16, 307.14, 293.16, 256.09, 224.11, 212.11, 168.08, 156.08, 144.08, 108.08, 107.07, 106.06	burnamine acid	L2	<0.1
**P20**	158	9.73	$[M+H]^+$	355.16523	355.16518	0.15	11	$C_{20}H_{22}N_2O_4$	337.15, 325.16, 307.14, 275.12, 247.12, 238.08, 214.09, 192.08, 108.08, 107.07, 106.06	burnamine acid isomer	L2	<0.1
**P40**	46	12.40	$[M+H]^+$	325.15467	325.15461	0.18	11	$C_{19}H_{20}N_2O_3$	307.14, 289.14, 279.15, 238.12, 220.11, 212.11, 206.10, 202.09, 194.09, 180.08, 174.09, 156.08, 144.08, 132.08, 120.08, 108.08, 107.07, 106.06	picrinine acid	L2	<0.1
**P68**	280	15.14	$[M+H]^+$	401.20710	401.20689	0.52	10	$C_{22}H_{28}N_2O_5$	369.18, 351.17, 340.17, 108.08, 106.06	CH_4O-burnamine	L2	<0.1<0.1
**P96**	77	18.38	$[M+H]^+$	369.18088	369.18068	0.55	11	$C_{21}H_{24}N_2O_4$	351.17, 339.17, 321.16, 289.13, 270.11, 252.10, 202.08, 184.08, 168.08, 156.08, 144.08, 108.08, 107.07, 106.06	burnamine★	L1	<0.1
**P104**	587	19.66	$[M+H]^+$	309.15975	309.15964	0.37	11	$C_{19}H_{20}N_2O_2$	291.14, 237.11, 222.09, 197.07, 180.08, 108.08	16-formyl-picrinine isomer	L2	<0.1
**P115**	60	20.81	$[M+H]^+$	309.15975	309.15961	0.47	11	$C_{19}H_{20}N_2O_2$	291.15, 279.15, 263.15, 246.13, 240.10, 222.09, 186.09, 136.08, 108.08, 106.06	16-formyl-picrinine	L2	<0.1
**P119**	20	21.26	$[M+H]^+$	309.15975	309.15958	0.57	11	$C_{19}H_{20}N_2O_2$	291.15, 281.16, 263.15, 249.14, 238.12, 220.11, 186.09, 172.08, 132.08, 124.08, 108.08, 107.07, 106.06	16-formyl-picrinine isomer	L2	0.53
**P120**	72	21.39	$[M+H]^+$	341.18597	341.18595	0.06	10	$C_{20}H_{24}N_2O_3$	325.15, 309.16, 291.15, 279.15, 249.14, 238.12,	dihydro-picrinine	L2	<0.1

Peak No.	FBMN node	RT (min)	Ion type	Precursor ion (calc.)	Precursor ion (exp.)	*Δ*ppm	DBE	Formula	Fragment ions	Annotation	Level ※	Average area (%)
									202.08, 186.09, 174.09, 124.08, 108.08, 107.07, 106.06			
**P121**	88	21.52	$[M+H]^+$	341.18597	341.18593	0.11	10	$C_{20}H_{24}N_2O_3$	325.15, 309.16, 291.15, 279.15, 249.14, 238.12, 202.08, 186.09, 174.09, 124.08, 108.08, 107.07, 106.06	dihydro-picrinine isomer	L2	<0.1
**P139**	no	23.16	$[M+H]^+$	339.17032	339.17001	0.91	11	$C_{20}H_{22}N_2O_3$	321.16, 263.15, 156.08, 121.08, 108.08	strictamine *N*-oxide☆	L2	<0.1
**P149**	113	23.91	$[M+H]^+$	309.15975	309.15966	0.31	11	$C_{19}H_{20}N_2O_2$	291.15, 240.10, 222.09, 186.09, 108.08, 106.06	16-formyl-picrinine like	L3	<0.1
**P150**	58	23.91	$[M+H]^+$	353.18597	353.18589	0.22	11	$C_{21}H_{24}N_2O_3$	321.16, 303.15, 293.17, 220.08, 144.08, 122.10, 120.08, 108.08	5*α*-methoxystrictamine★	L1	<0.1
**P160**	1	24.47	$[M+H]^+$	339.17032	339.17019	0.38	11	$C_{20}H_{22}N_2O_3$	321.16, 307.18, 279.15, 270.11, 261.14, 252.10, 237.11, 206.10, 174.09, 156.08, 144.08, 120.08, 108.08, 107.07, 106.06	picrinine★	L1	14.8
**P163**	37	24.62	$[M+H]^+$	371.19653	371.19641	0.33	10	$C_{21}H_{26}N_2O_4$	339.17, 321.16, 309.16, 296.13, 279.15, 270.11, 216.10, 202.09, 120.08, 108.08, 107.07, 106.06	CH_4O-picrinine	L2	<0.1
**P165**	117	24.78	$[M+H]^+$	339.17032	339.17015	0.50	11	$C_{20}H_{22}N_2O_3$	321.16, 309.16, 296.13, 289.13, 279.15, 206.10, 174.09, 156.08, 144.08, 120.08, 108.08, 107.07, 106.06	picrinine isomer▲	L2	<0.1
**P168**	215	24.96	$[M+H]^+$	383.19653	383.19637	0.43	11	$C_{22}H_{26}N_2O_4$	337.15, 277.13, 268.10, 231.09, 202.09, 122.06, 108.08, 106.06	C_2H_4O-picrinine	L2	<0.1
**P169**	6	25.00	$[M+H]^+$	367.16523	367.16499	0.67	12	$C_{21}H_{22}N_2O_4$	349.15, 335.14, 321.16, 307.14, 289.13, 279.15, 270.11, 252.10, 220.08, 184.08, 156.08, 108.08, 107.07, 106.06	picralinal★	L1	3.49
**P170**	228	25.03	$[M+H]^+$	337.19105	337.19095	0.31	11	$C_{21}H_{24}N_2O_2$	305.16, 279.18, 270.11, 222.09, 144.08, 108.08, 107.07, 106.06	dideoxy-burnamine	L2	<0.1

Peak No.	FBMN node	RT (min)	Ion type	Precursor ion (calc.)	Precursor ion (exp.)	*Δ*ppm	DBE	Formula	Fragment ions	Annotation	Level ※	Average area (%)
**P190**	485	26.41	$[M+H]^+$	381.21727	381.21701	0.68	11	$C_{23}H_{28}N_2O_3$	138.09, 110.05	16-formyl-5*α*-methoxylstrictamine▲	L2	<0.1
**P196**	136	26.61	$[M+H]^+$	371.19653	371.19650	0.09	10	$C_{21}H_{26}N_2O_4$	339.17, 321.16, 309.16, 296.13, 279.15, 270.11, 216.10, 120.08, 108.08, 107.07, 106.06	CH_4O-picrinine isomer	L2	<0.1
**P197**	28	26.61	$[M+H]^+$	339.17032	339.17022	0.29	11	$C_{20}H_{22}N_2O_3$	339.17, 321.16, 309.16, 296.13, 279.15, 270.11, 120.08, 108.08, 107.07, 106.06	picrinine isomer▲	L2	0.93
**P202**	no	27.21	$[M+H]^+$	411.19145	411.19129	0.39	12	$C_{23}H_{26}N_2O_5$	393.18, 178.08, 136.08	scholarisine B☆	L1	<0.1
**P206**	no	27.51	$[M+H]^+$	383.19653	383.19628	0.66	11	$C_{22}H_{26}N_2O_4$	280.13, 158.10, 144.08, 121.08	(-)-pseudoakuammigine *N*-oxide▲	L2	<0.1
**P212**	171	28.23	$[M+H]^+$	385.21218	385.21214	0.11	10	$C_{22}H_{28}N_2O_4$	339.17, 120.08, 108.08, 107.07, 106.06	C_2H_6O-picrinine	L2	0.84
**P219**	244	30.49	$[M+H]^+$	355.16523	355.16509	0.41	11	$C_{20}H_{22}N_2O_4$	108.08	O-picrinine	L2	<0.1
								**Vallesamine-type MIAs**				
**P2**	394	5.51	$[M+H]^+$	357.18088	357.18062	0.74	10	$C_{20}H_{24}N_2O_4$	339.17, 329.18, 311.18, 250.09, 241.13, 158.110, 156.08, 152.11, 114.09, 110.10	O-6,7-*seco*-angustilobine B	L2	<0.1
**P6**	1165	7.54	$[M+H]^+$	355.16523	355.16522	0.04	11	$C_{20}H_{22}N_2O_4$	339.17, 337.15, 311.17, 307.14, 293.17, 289.13, 282.15, 264.14, 200.07, 144.08, 120.08, 108.08	dehydro-O-6,7-*seco*-angustilobine B	L2	<0.1
**P27**	200	11.01	$[M+H]^+$	373.17580	373.17579	0.02	10	$C_{20}H_{24}N_2O_5$	224.11, 216.10, 156.08	O-6,7-*seco*-angustilobine B hydrate	L2	<0.1
**P46**	437	12.93	$[M+H]^+$	343.16523	343.16519	0.13	10	$C_{19}H_{22}N_2O_4$	325.15, 299.21, 281.20, 269.10, 110.10, 108.08	alstoniascholarine A oxide	L2	<0.1
**P48**	322	13.06	$[M+H]^+$	355.16523	355.16518	0.15	11	$C_{20}H_{22}N_2O_4$	325.15, 216.10, 156.08, 140.07	O-6,7-*seco*-angustilobine B like	L3	<0.1
**P53**	22	13.69	$[M+H]^+$	327.17032	327.17023	0.27	10	$C_{19}H_{22}N_2O_3$	283.18, 265.17, 253.17, 236.14, 222.13, 174.09, 156.08, 144.08, 134.10, 110.10, 108.08	alstoniascholarine A★	L1	2.39
**P59**	417	14.20	$[M+H]^+$	383.19653	383.19631	0.59	11	$C_{22}H_{26}N_2O_4$	368.17, 353.18, 290.12, 280.13, 262.12, 248.11, 220.11, 212.07, 180.10, 166.09, 150.09, 138.09,	C_2H_2-vallesamine *N*-oxide	L2	<0.1

Peak No.	FBMN node	RT (min)	Ion type	Precursor ion (calc.)	Precursor ion (exp.)	*Δ*ppm	DBE	Formula	Fragment ions	Annotation	Level ※	Average area (%)
									130.07, 120.08			
**P60**	38	14.32	$[M+H]^+$	341.18597	341.18571	0.76	10	$C_{20}H_{24}N_2O_3$	297.20, 279.18, 264.16, 236.14, 222.13, 148.11, 124.11, 122.10, 108.08	methyl-alstoniascholarine A	L2	2.05
**P64**	279	14.68	$[M+H]^+$	369.18088	369.18064	0.66	11	$C_{21}H_{24}N_2O_4$	353.19, 282.15, 252.10, 214.08, 188.07, 168.10, 122.10,	CO-6,7-*seco*-angustilobine B	L2	<0.1
**P74**	945	15.65	$[M+H]^+$	369.18088	369.18073	0.42	11	$C_{21}H_{24}N_2O_4$	309.16, 252.10, 144.08, 110.10, 108.08	vallesamine hydrate	L2	<0.1
**P76**	234	16.05	$[M+H]^+$	309.15975	309.15974	0.05	11	$C_{19}H_{20}N_2O_2$	265.17, 236.14, 122.10, 108.08	dehydrated alstoniascholarine A	L2	<0.1
**P86**	185	17.07	$[M+H]^+$	325.15467	325.15456	0.34	11	$C_{19}H_{20}N_2O_3$	307.18, 294.14, 278.12, 263.12, 252.10, 246.09, 202.09, 196.11, 130.07, 108.08, 106.06	dehydro-alstoniascholarine A	L2	<0.1
**P88**	80	17.38	$[M+H]^+$	343.16523	343.16514	0.27	10	$C_{19}H_{22}N_2O_4$	325.15, 299.18, 282.17, 281.16, 263.15, 236.14, 222.13, 208.11, 194.10, 172.07, 156.08, 150.09, 144.08, 132.08, 126.09, 108.08	dimethyl-vallesamine *N*-oxide	L2	<0.1
**P91**	4	17.57	$[M+H]^+$	341.18597	341.18592	0.14	10	$C_{20}H_{24}N_2O_3$	323.18, 311.18, 291.15, 282.15, 279.15, 250.12, 232.10, 222.13, 214.09, 202.09, 192.10, 182.10, 172.08, 144.08, 110.10, 108.08	vallesamine★	L1	12.6
**P92**	9	17.72	$[M+H]^+$	355.20162	355.20159	0.08	10	$C_{21}H_{26}N_2O_3$	337.19, 323.18, 282.15, 323.10, 140.11, 124.11, 123.10, 122.10, 108.08	methyl-vallesamine	L2	<0.1
**P93**	161	18.02	$[M+H]^+$	341.18597	341.18588	0.26	10	$C_{20}H_{24}N_2O_3$	323.18, 311.18, 282.15, 279.15, 232.10, 214.09, 192.10, 172.08, 144.08, 126.09, 110.10, 108.08	vallesamine isomer▲	L2	<0.1
**P98**	669	18.96	$[M+H]^+$	309.15975	309.15965	0.34	11	$C_{19}H_{20}N_2O_2$	156.08, 122.10, 108.08	(de)CH_4O-vallesamine	L2	<0.1
**P116**	17	21.02	$[M+H]^+$	357.18088	357.18072	0.46	10	$C_{20}H_{24}N_2O_4$	339.17, 327.17, 322.17, 295.14, 232.10, 222.09, 214.09, 208.10, 202.09, 154.06, 126.09, 124.08, 112.08, 108.08	vallesamine *N*-oxide★	L1	2.13

Peak No.	FBMN node	RT (min)	Ion type	Precursor ion (calc.)	Precursor ion (exp.)	Δppm	DBE	Formula	Fragment ions	Annotation	Level ※	Average area (%)
**P117**	75	21.09	$[M+H]^+$	337.19105	337.19089	0.49	11	$C_{21}H_{24}N_2O_2$	322.17, 294.15, 277.17, 263.15, 248.11, 234.13, 220.11, 208.10, 196.10, 183.09, 154.07, 136.11, 108.08	dehydrated methyl-vallesamine	L2	<0.1
**P137**	74	23.02	$[M+H]^+$	341.18597	341.18581	0.47	10	$C_{20}H_{24}N_2O_3$	323.17, 281.16, 224.13, 214.08, 202.09, 142.06, 140.11, 130.06, 122.10, 110.10	6,7-*seco*-angustilobine B★	L1	<0.1
**P151**	107	24.03	$[M+H]^+$	327.17032	327.17026	0.18	10	$C_{19}H_{22}N_2O_3$	310.14, 265.13, 254.12, 222.09, 190.09, 158.09, 144.08, 130.06, 108.08, 106.06	demethyl-vallesamine	L2	<0.1
**P210**	359	28.06	$[M+H]^+$	371.19653	371.19638	0.42	10	$C_{21}H_{26}N_2O_4$	267.15, 250.12, 202.09, 152.11, 142.06, 110.10, 108.08	CH_2-vallesamine *N*-oxide	L2	<0.1
								**Alstolactine-type MIAs**				
**P19**	255	9.59	$[M+H]^+$	401.20710	401.20688	0.55	10	$C_{22}H_{28}N_2O_5$	369.18, 339.17, 122.10, 120.08, 108.08	alstolactine-like	L3	<0.1
**P50**	393	13.27	$[M+H]^+$	371.19653	371.19623	0.82	10	$C_{21}H_{26}N_2O_4$	341.19, 321.16, 180.10, 160.08, 138.09, 122.10, 110.10, 108.08	alstolactine-like	L3	<0.1
**P71**	96	15.29	$[M+H]^+$	415.22275	415.22271	0.09	10	$C_{23}H_{30}N_2O_5$	383.20, 353.19, 333.16, 321.16, 230.08, 208.13, 122.10, 120.08, 108.08	alstolactine-like	L3	<0.1
**P72**	63	15.39	$[M+H]^+$	373.21218	373.21199	0.52	9	$C_{21}H_{28}N_2O_4$	341.19, 323.18, 309.16, 291.15, 281.16, 218.12, 186.09, 144.08, 124.08, 108.08, 106.06	alstolactine-like	L3	<0.1
**P82**	81	16.67	$[M+H]^+$	387.22783	387.22768	0.40	9	$C_{22}H_{30}N_2O_4$	369.21, 355.20, 337.19, 323.17, 305.17, 166.12, 152.11, 138.09, 122.10, 120.08	alstolactine-like	L3	0.84
**P83**	323	16.72	$[M+H]^+$	385.21218	385.21201	0.45	10	$C_{22}H_{28}N_2O_4$	367.20, 355.20, 337.19, 323.18, 220.11, 166.12, 138.09, 122.10, 120.08	alstolactine-like	L3	<0.1
**P111**	no	20.22	$[M+H]^+$	357.14450	357.14421	0.81	11	$C_{19}H_{20}N_2O_5$	311.14, 124.08	alstoniascholarine L☆	L1	<0.1
**P114**	91	20.64	$[M+H]^+$	355.20162	355.20139	0.65	10	$C_{21}H_{26}N_2O_3$	323.18, 305.17, 293.17, 240.10, 144.08, 122.10, 110.10	alstolactine-like	L3	<0.1

Peak No.	FBMN node	RT (min)	Ion type	Precursor ion (calc.)	Precursor ion (exp.)	Δppm	DBE	Formula	Fragment ions	Annotation	Level ※	Average area (%)
**P118**	no	21.15	$[M+H]^+$	357.14450	357.14433	0.47	11	$C_{19}H_{20}N_2O_5$	311.14, 124.08	alstoniascholarine M☆	L1	<0.1
**P125**	116	21.88	$[M+H]^+$	387.22783	387.22773	0.27	9	$C_{22}H_{30}N_2O_4$	355.20, 337.19, 323.18, 305.16, 138.09, 122.10, 120.08	alstolactine-like	L3	<0.1
**P127**	106	22.02	$[M+H]^+$	355.20162	355.20160	0.05	10	$C_{21}H_{26}N_2O_3$	323.18, 205.16, 295.18, 186.09, 172.07, 144.08, 138.09, 122.10	alstolactine-like	L3	<0.1
**P128**	111	22.07	$[M+H]^+$	385.17580	385.17556	0.62	11	$C_{21}H_{24}N_2O_5$	367.16, 353.19, 339.17, 289.13, 281.13, 202.09, 138.09, 120.08, 110.06	alstolactine D isomer▲	L2	<0.1
**P130**	195	22.31	$[M+H]^+$	357.21727	357.21701	0.73	9	$C_{21}H_{28}N_2O_3$	339.17, 238.12, 216.10, 150.09, 124.11, 110.10, 108.08, 106.06	CO-alstolactine D	L2	<0.1
**P136**	190	22.97	$[M+H]^+$	401.20710	401.20692	0.45	10	$C_{22}H_{28}N_2O_5$	369.18, 351.18, 337.15, 309.16, 295.14, 138.09, 110.06	alstolactine D isomer *N*-oxide 1	L2	<0.1
**P138**	173	23.11	$[M+H]^+$	401.20710	401.20694	0.40	10	$C_{22}H_{28}N_2O_5$	369.18, 351.18, 337.15, 309.16, 295.14, 138.09, 110.06	CH_4-alstolactine D isomer	L2	<0.1
**P164**	199	24.77	$[M+H]^+$	355.20162	355.20156	0.17	10	$C_{21}H_{26}N_2O_3$	323.18, 305.16, 295.18, 186.09, 172.08, 138.09, 122.10, 120.08, 108.08	alstolactine-like	L3	<0.1
**P145**	403	23.65	$[M+H]^+$	401.20710	401.20698	0.30	10	$C_{22}H_{28}N_2O_5$	383.19, 369.18, 337.15, 325.15, 138.09, 122.10, 110.10	alstolactine D isomer *N*-oxide 2	L2	<0.1
**P146**	253	23.70	$[M+H]^+$	355.20162	355.20139	0.65	10	$C_{21}H_{26}N_2O_3$	339.17, 323.18, 305.17, 268.13, 240.10, 210.09, 202.08, 122.10, 110.10, 108.08	alstolactine-like	L3	<0.1
**P166**	311	24.83	$[M+H]^+$	399.19145	399.19131	0.35	11	$C_{22}H_{26}N_2O_5$	310.11, 180.08, 138.09, 110.06	CH_2-scholarisine L	L2	<0.1
**P167**	164	24.88	$[M+H]^+$	369.21727	369.21702	0.68	10	$C_{22}H_{28}N_2O_3$	337.19, 248.10, 138.09, 108.08, 107.07, 106.06	alstolactine-like	L3	<0.1
**P171**	no	25.05	$[M+H]^+$	371.16015	371.16001	0.37	11	$C_{20}H_{22}N_2O_5$	325.15, 281.13, 156.10, 138.09, 121.09, 112.08, 110.06, 94.07	alstolactine A☆	L1	<0.1
**P172**	no	25.07	$[M+H]^+$	371.16015	371.16005	0.27	11	$C_{20}H_{22}N_2O_5$	325.15, 281.13, 156.10, 138.09, 121.09, 112.08, 110.06, 94.07	alstolactine B☆	L1	<0.1
**P173**	439	25.08	$[M+H]^+$	399.19145	399.19119	0.65	11	$C_{22}H_{26}N_2O_5$	369.18, 339.14, 320.12,	CH_2-alstolactine D like	L3	<0.1

Peak No.	FBMN node	RT (min)	Ion type	Precursor ion (calc.)	Precursor ion (exp.)	Δppm	DBE	Formula	Fragment ions	Annotation	Level ※	Average area (%)
									310.11, 263.12, 184.08, 142.09, 136.08, 124.07			
**P176**	97	25.45	$[M+H]^+$	385.17580	385.17567	0.33	11	$C_{21}H_{24}N_2O_5$	353.15, 341.15, 262.11, 202.09, 124.08, 96.04, 80.05	scholarisine E★	L1	<0.1
**P177**	83	25.64	$[M+H]^+$	369.18088	369.18077	0.31	11	$C_{21}H_{24}N_2O_4$	122.10, 120.08, 108.08, 106.06	alstolactine-like	L3	<0.1
**P178**	272	25.64	$[M+H]^+$	399.19145	399.19141	0.10	11	$C_{22}H_{26}N_2O_5$	367.16, 310.11, 263.12, 208.07, 180.08, 136.07, 110.06	CH_2-alstolactine D	L2	<0.1
**P191**	110	26.47	$[M+H]^+$	385.17580	385.17549	0.80	11	$C_{21}H_{24}N_2O_5$	367.16, 341.15, 309.12, 295.14, 156.10, 138.09, 121.09, 112.08, 110.06, 94.07	alstolactine D	L2	<0.1
**P194**	no	26.60	$[M+H]^+$	385.17580	385.17556	0.62	11	$C_{21}H_{24}N_2O_5$	367.16, 341.15, 309.12, 295.14, 156.10, 138.09, 121.09, 112.08, 110.06, 94.07	alstolactine E	L2	<0.1
**P195**	138	26.61	$[M+H]^+$	353.14958	353.14955	0.10	12	$C_{20}H_{20}N_2O_4$	293.13, 280.13, 220.11, 122.06, 108.08	(de)CH_4O-scholarisine E 1	L2	<0.1
**P200**	82	26.83	$[M+H]^+$	353.14958	353.14951	0.21	12	$C_{20}H_{20}N_2O_4$	335.14, 307.14, 293.13, 280.13, 266.12, 247.12, 230.08, 220.11, 170.06, 122.10, 120.08, 108.08, 106.06	(de)CH_4O-scholarisine E 2	L2	<0.1
**P209**	217	27.90	$[M+H]^+$	399.19145	399.19126	0.47	11	$C_{22}H_{26}N_2O_5$	353.15, 293.13, 266.12	CH_2-scholarisine E 1	L2	<0.1
**P211**	309	28.11	$[M+H]^+$	399.19145	399.19136	0.22	11	$C_{22}H_{26}N_2O_5$	353.15, 294.11, 293.13, 266.12	CH_2-scholarisine E 2	L2	<0.1
**P214**	265	28.55	$[M+H]^+$	353.18597	353.18588	0.25	11	$C_{21}H_{24}N_2O_3$	335.14, 294.11, 293.13, 275.12, 266.12, 232.11, 122.10, 120.08, 108.08	alstolactine-like	L3	<0.1
**P215**	445	28.62	$[M+H]^+$	399.19145	399.19138	0.17	11	$C_{22}H_{26}N_2O_5$	353.19, 294.11, 293.13, 275.12, 266.12, 232.11, 122.10, 120.08, 108.08	CH_2-scholarisine E 3	L2	<0.1
**P217**	78	29.40	$[M+H]^+$	355.16523	355.16509	0.41	11	$C_{20}H_{22}N_2O_4$	337.15, 265.13, 122.10, 112.11, 111.10, 110.10	CH_2O-scholarisine M	L2	<0.1
**P218**	1498	29.62	$[M+H]^+$	355.16523	355.16510	0.38	11	$C_{20}H_{22}N_2O_4$	138.09, 110.06	CH_2O-scholarisine L	L2	<0.1
**P220**	148	31.76	$[M+H]^+$	385.17580	385.17551	0.75	11	$C_{21}H_{24}N_2O_5$	367.16, 341.15, 295.14, 138.09, 121.09, 110.06, 94.07	scholarisine M★	L1	<0.1
**P221**	89	32.03	$[M+H]^+$	385.17580	385.17545	0.91	11	$C_{21}H_{24}N_2O_5$	367.16, 341.15, 295.14,	scholarisine L★	L1	<0.1

Peak No.	FBMN node	RT (min)	Ion type	Precursor ion (calc.)	Precursor ion (exp.)	Δppm	DBE	Formula	Fragment ions	Annotation	Level ※	Average area (%)
									138.09, 121.09, 110.06, 94.07			
								**Yohimbine-type MIAs**				
**P62**	400	14.51	$[M+H]^+$	355.16523	355.16515	0.24	11	$C_{20}H_{22}N_2O_4$		19*E*-geissoschizine acid oxide	L2	<0.1
**P63**	582	14.59	$[M+H]^+$	355.20162	355.20148	0.39	10	$C_{21}H_{26}N_2O_3$	337.19	methyl-yohimbine acid	L2	<0.1
**P78**	721	16.29	$[M+H]^+$	369.21727	369.21699	0.76	10	$C_{22}H_{28}N_2O_3$	351.21, 296.17, 124.11	dimethyl-yohimbine acid	L2	<0.1
**P99**	226	19.11	$[M+H]^+$	355.20162	355.20145	0.48	10	$C_{21}H_{26}N_2O_3$	323.17, 251.15, 212.13, 144.08, 122.10, 108.08	yohimbine isomer methyl-17-hydroxyyohimban-16-carboxylate	L2	<0.1
**P102**	641	19.27	$[M+H]^+$	371.19653	371.19643	0.28	10	$C_{21}H_{26}N_2O_4$	341.19, 323.17, 253.17, 252.16, 251.15, 228.12, 144.08, 122.10, 108.08	yohimbine *N*-oxide	L2	<0.1
**P107**	31	19.83	$[M+H]^+$	355.20162	355.20156	0.17	10	$C_{21}H_{26}N_2O_3$	323.17, 251.15, 224.13, 212.13, 144.08, 122.10, 108.08	yohimbine▲	L2	0.27
**P110**	127	20.03	$[M+H]^+$	369.21727	369.21698	0.79	10	$C_{22}H_{28}N_2O_3$	338.17, 326.17, 222.13, 197.11, 144.08, 130.06, 122.10, 108.08	methyl-yohimbine	L2	<0.1
**P123**	44	21.66	$[M+H]^+$	355.20162	355.20146	0.45	10	$C_{21}H_{26}N_2O_3$	323.17, 251.15, 224.13, 212.13, 144.08, 122.10, 108.08	yohimbine isomer	L2	<0.1
**P129**	343	22.16	$[M+H]^+$	369.18088	369.18066	0.61	11	$C_{21}H_{24}N_2O_4$	337.16, 309.16, 295.14, 160.08, 138.09, 122.10, 110.06	ajmalicine *N*-oxide	L2	<0.1
**P147**	281	23.71	$[M+H]^+$	353.18597	353.18591	0.17	11	$C_{21}H_{24}N_2O_3$	321.16, 251.16, 210.11, 170.10, 144.08, 108.08	19*E*-geissoschizine★	L1	0.43
**P152**	119	24.08	$[M+H]^+$	339.17032	339.17002	0.88	11	$C_{20}H_{22}N_2O_3$	144.08, 122.10, 108.08	demethyl-ajmalicine	L2	<0.1
**P154**	10801	24.21	$[M+H]^+$	353.18597	353.18594	0.08	11	$C_{21}H_{24}N_2O_3$	335.17, 323.17, 251.15, 210.11, 170.10, 144.08, 122.10, 108.08	ajmalicine isomer	L2	<0.1
**P157**	218	24.37	$[M+H]^+$	353.18597	353.18588	0.25	11	$C_{21}H_{24}N_2O_3$	335.17, 323.17, 251.15, 210.11, 178.09, 170.10, 144.08	ajmalicine★	L1	<0.1
**P180**	286	25.76	$[M+H]^+$	325.19105	325.19089	0.51	10	$C_{20}H_{24}N_2O_2$	293.16, 222.09, 144.08, 108.08	16-formyl-17-hydroxyl-yohimban	L2	<0.1
**P199**	285	26.81	$[M+H]^+$	367.20162	367.20139	0.63	11	$C_{22}H_{26}N_2O_3$	324.16, 280.13, 144.08, 108.08	yohimbine-like	L3	<0.1

Peak No.	FBMN node	RT (min)	Ion type	Precursor ion (calc.)	Precursor ion (exp.)	Δppm	DBE	Formula	Fragment ions	Annotation	Level ※	Average area (%)
									**Alstoscholarisine-type MIAs**			
**P34**	543	11.80	$[M+H]^+$	369.18088	369.18066	0.61	11	$C_{21}H_{24}N_2O_4$	351.17, 339.17, 321.16, 279.15, 256.13, 250.09, 224.11, 196.11, 180.08, 170.09, 146.05	CO-alstoscholarisine I acid	L2	<0.1
**P32**	317	11.48	$[M+H]^+$	283.18049	283.18028	0.74	9	$C_{18}H_{22}N_2O$	265.17, 210.13, 194.10, 182.10, 158.10, 140.11, 122.10, 108.08	demethyl-alstoscholarisine H 1	L2	<0.1
**P49**	71	13.16	$[M+H]^+$	325.19105	325.19095	0.32	10	$C_{20}H_{24}N_2O_2$	307.18, 293.16, 275.15, 264.14, 246.13, 236.14, 231.10, 220.11, 206.10, 194.10, 182.10, 180.08, 168.08, 144.08, 130.07, 122.10,	(de)CH_2O-alstoscholarisine I	L2	<0.1
**P56**	297	14.01	$[M+H]^+$	373.21218	373.21205	0.36	9	$C_{21}H_{28}N_2O_4$	297.20, 279.18, 222.13, 148.11, 125.12, 122.10, 110.10	$C_2H_4O_3$-alstoscholarisine H	L2	<0.1
**P57**	238	14.07	$[M+H]^+$	283.18049	283.18037	0.42	9	$C_{18}H_{22}N_2O$	265.17, 248.14, 240.13, 224.14, 176.11, 158.10, 144.07, 110.10, 108.08	demethyl-alstoscholarisine H 2	L2	<0.1
**P69**	186	15.17	$[M+H]^+$	293.16484	293.16468	0.55	11	$C_{19}H_{20}N_2O$	265.17, 250.13, 232.11, 220.11, 186.09, 180.08, 171.09, 168.08, 144.08	alstoscholarisine-like	L3	<0.1
**P73**	40	15.49	$[M+H]^+$	329.18597	329.18590	0.21	9	$C_{19}H_{24}N_2O_3$	311.18, 299.18, 282.15, 267.15, 251.15, 230.12, 220.09, 202.09, 110.10, 108.08	dioxy-alstoscholarisine H	L2	1.29
**P87**	262	17.09	$[M+H]^+$	343.20162	343.20155	0.20	9	$C_{20}H_{26}N_2O_3$	325.19, 313.19, 256.13, 222.12, 202.09, 124.11, 122.10, 110.10	CH_2O_2-alstoscholarisine H	L2	<0.1
**P90**	152	17.38	$[M+H]^+$	299.17540	299.17515	0.85	9	$C_{18}H_{22}N_2O_2$	282.17, 264.16, 249.14, 236.14, 222.13, 208.11, 174.09, 156.08, 144.08, 126.09	alstoscholarisine-like	L3	<0.1
**P95**	no	18.36	$[M+H]^+$	341.18597	341.18580	0.50	10	$C_{20}H_{24}N_2O_3$	297.19, 240.14, 210.13, 196.11, 96.08, 44.04	alstoscholarisine D☆	L1	<0.1
**P100**	122	19.11	$[M+H]^+$	341.18597	341.18579	0.53	10	$C_{20}H_{24}N_2O_3$	323.18, 309.15, 297.20, 254.12, 248.12, 240.14, 222.13, 122.10, 108.08	alstoscholarisine C acid	L2	<0.1

Peak No.	FBMN node	RT (min)	Ion type	Precursor ion (calc.)	Precursor ion (exp.)	Δppm	DBE	Formula	Fragment ions	Annotation	Level ※	Average area (%)
**P103**	428	19.47	$[M+H]^+$	297.19614	297.19585	0.98	9	$C_{19}H_{24}N_2O$	279.18, 264.16, 249.13, 237.15, 222.13, 148.11, 122.10	alstoscholarisine H isomer 1	L2	<0.1
**P105**	381	19.66	$[M+H]^+$	341.18597	341.18585	0.35	10	$C_{20}H_{24}N_2O_3$	323.17, 309.16, 291.15, 263.16, 108.08	demethyl-alstoscholarisine I	L2	<0.1
**P108**	10806	19.98	$[M+H]^+$	297.19614	297.19589	0.84	9	$C_{19}H_{24}N_2O$	279.18, 264.16, 249.13, 236.14, 222.13, 208.11, 148.11, 144.08, 133.09, 122.10	alstoscholarisine H isomer 2	L2	<0.1
**P112**	174	20.27	$[M+H]^+$	297.19614	297.19598	0.54	9	$C_{19}H_{24}N_2O$	279.19, 264.16, 248.14, 236.14, 233.12, 222.13, 148.11, 144.08, 122.10, 107.08	alstoscholarisine H★	L1	<0.1
**P113**	231	20.47	$[M+H]^+$	387.22783	387.22761	0.58	9	$C_{22}H_{30}N_2O_4$	369.18, 355.20, 337.19, 325.19, 282.15, 140.11, 123.10, 122.10, 110.10, 108.08	CH_4O-alstoscholarisine B	L2	<0.1
**P124**	205	21.75	$[M+H]^+$	297.19614	297.19599	0.51	9	$C_{19}H_{24}N_2O$	279.18, 248.14, 240.13, 222.13, 210.13, 209.12, 122.10	alstoscholarisine A★	L1	<0.1
**P135**	15	22.92	$[M+H]^+$	355.20162	355.20159	0.08	10	$C_{21}H_{26}N_2O_3$	337.19, 325.19, 293.17, 265.17, 240.10, 170.10, 122.10	alstoscholarisine I★	L1	1.73
**P141**	no	23.33	$[M+H]^+$	297.19614	297.19612	0.07	9	$C_{19}H_{24}N_2O$	240.14, 210.13, 196.11, 96.08	alstoscholarisine E☆	L1	<0.1
**P142**	27	23.35	$[M+H]^+$	355.20162	355.20152	0.28	10	$C_{21}H_{26}N_2O_3$	339.17, 323.17, 295.18, 279.15, 220.11, 205.09, 122.10, 108.08	C_2H_2-alstoscholarisine A	L2	0.55
**P144**	99	23.57	$[M+H]^+$	297.19614	297.19591	0.78	9	$C_{19}H_{24}N_2O$	279.18, 264.16, 236.14, 222.13, 148.11, 125.12, 124.11, 122.10, 110.10	alstoscholarisine H isomer 3	L2	<0.1
**P179**	126	25.74	$[M+H]^+$	355.20162	355.20138	0.68	10	$C_{21}H_{26}N_2O_3$	323.18, 296.19, 251.15, 228.10, 208.11, 168.13, 144.08, 122.10, 110.10, 96.08, 44.05	alstoscholarisine C★	L1	<0.1
**P182**	696	26.00	$[M+H]^+$	297.19614	297.19596	0.61	9	$C_{19}H_{24}N_2O$	279.19, 125.12, 124.11, 110.10	alstoscholarisine H isomer 4	L2	<0.1
**P183**	469	26.02	$[M+H]^+$	355.20162	355.20155	0.20	10	$C_{21}H_{26}N_2O_3$	323.18, 296.19, 251.15, 208.11, 144.08, 122.10, 110.10, 96.08, 44.05	alstoscholarisine C isomer 5	L2	<0.1

Peak No.	FBMN node	RT (min)	Ion type	Precursor ion (calc.)	Precursor ion (exp.)	Δppm	DBE	Formula	Fragment ions	Annotation	Level ※	Average area (%)
**P187**	213	26.20	$[M+H]^+$	355.20162	355.20144	0.51	10	$C_{21}H_{26}N_2O_3$	323.18, 296.19, 268.13, 251.15, 228.10, 208.11, 168.13, 144.08, 122.10, 110.10, 96.08, 44.05	alstoscholarisine B★	L1	<0.1
**P189**	553	26.39	$[M+H]^+$	297.19614	297.19588	0.88	9	$C_{19}H_{24}N_2O$	279.18, 125.15, 124.11, 110.10	alstoscholarisine H isomer 6	L2	<0.1
**P192**	242	26.51	$[M+H]^+$	311.21179	311.21165	0.45	9	$C_{20}H_{26}N_2O$	279.19, 264.16, 248.14, 236.14, 236.14, 222.13, 148.11, 144.08, 122.10, 107.09	CH_2-alstoscholarisine H	L2	<0.1
**P193**	506	26.53	$[M+H]^+$	297.19614	297.19593	0.71	9	$C_{19}H_{24}N_2O$	193.09, 122.10, 110.10	alstoscholarisine H isomer 7	L2	<0.1
**P198**	478	26.70	$[M+H]^+$	297.19614	297.19607	0.24	9	$C_{19}H_{24}N_2O$	279.18, 125.12, 124.11, 110.10	alstoscholarisine H isomer 8	L2	<0.1
**P216**	493	28.96	$[M+H]^+$	355.16523	355.16508	0.43	11	$C_{20}H_{22}N_2O_4$	264.10, 236.11, 208.11	alstoscholarisine-like	L3	0.77
**P224**	630	34.00	$[M+H]^+$	295.18049	295.18038	0.37	10	$C_{19}H_{22}N_2O$	277.17, 253.17, 236.14, 208.11, 182.10, 171.09, 144.08, 108.08	dehydro-alstoscholarisine A	L2	<0.1
								**Scholarisine-type MIAs**				
**P10**	no	8.37	$[M+H]^+$	325.19105	325.19101	0.14	10	$C_{20}H_{24}N_2O_2$	180.08, 168.08, 156.08, 144.08, 130.06, 124.11, 122.10, 118.06, 96.08, 94.06, 70.06, 58.06, 44.05	scholarisine J☆	L1	<0.1
**P30**	225	11.24	$[M+H]^+$	309.15975	309.15962	0.44	11	$C_{19}H_{20}N_2O_2$	291.15, 280.13, 265.17, 220.11, 208.11, 168.08, 160.08, 156.08, 144.08, 122.10, 108.08	demethyl-scholarisine I 1	L2	<0.1
**P67**	458	15.02	$[M+H]^+$	323.17540	323.17519	0.67	11	$C_{20}H_{22}N_2O_2$	122.1	scholarisine I★	L1	<0.1
**P156**	377	24.33	$[M+H]^+$	309.15975	309.15959	0.53	11	$C_{19}H_{20}N_2O_2$	291.15, 281.16, 249.14, 238.12, 186.09, 158.09, 144.08, 132.08, 108.08, 107.07, 106.06	demethyl-scholarisine I 2	L2	<0.1
**P158**	no	24.39	$[M+H]^+$	307.14410	307.14396	0.47	12	$C_{19}H_{18}N_2O_2$	168.08, 144.08, 130.06, 108.08	scholarisine A☆	L1	<0.1
								**Vallesiachotamine-type MIAs**				
**P225**	34	34.57	$[M+H]^+$	351.17032	351.17023	0.25	12	$C_{21}H_{22}N_2O_3$	319.14, 249.10, 170.09, 107.05	vallesiachotamine★	L1	0.91
**P226**	47	34.87	$[M+H]^+$	351.17032	351.17013	0.54	12	$C_{21}H_{22}N_2O_3$	319.14, 249.10, 170.09, 107.05	isovallesiachotamine★	L1	0.66

Peak No.	FBMN node	RT (min)	Ion type	Precursor ion (calc.)	Precursor ion (exp.)	Δppm	DBE	Formula	Fragment ions	Annotation	Level ※	Average area (%)
						**Alstoscholarine-type MIAs**						
**P227**	no	35.31	$[M+H]^+$	361.15467	361.15441	0.72	14	$C_{22}H_{20}N_2O_3$	301.13, 273.14, 172.08, 144.08	*Z*-alstoscholarine☆	L1	<0.1
**P228**	no	35.78	$[M+H]^+$	361.15467	361.15451	0.44	14	$C_{22}H_{20}N_2O_3$	301.13, 273.14, 172.08, 144.08	*E*-alstoscholarine☆	L1	<0.1
						**Unclassified MIAs**						
**P1**	2073	4.72	$[M+H]^+$	355.16523	355.16519	0.12	11	$C_{20}H_{22}N_2O_4$	311.18, 283.15, 160.08,	unknown	L4	<0.1
**P3**	836	5.87	$[M+H]^+$	343.16523	343.16498	0.74	10	$C_{19}H_{22}N_2O_4$	325.15, 271.14, 160.07, 108.08	unknown	L4	<0.1
**P4**	899	6.01	$[M+H]^+$	311.17540	311.17536	0.14	10	$C_{19}H_{22}N_2O_2$	293.16, 283.18, 229.10, 211.09, 201.10, 188.07, 122.10, 110.09, 108.08	unknown	L4	<0.1
**P5**	305	6.77	$[M+H]^+$	299.17540	299.17535	0.18	9	$C_{18}H_{22}N_2O_2$	281.16, 253.14, 210.09, 198.09, 174.09, 160.07, 122.10, 110.10, 108.08, 107.07, 106.06	unknown	L4	<0.1
**P7**	252	7.84	$[M+H]^+$	295.18049	295.18030	0.65	10	$C_{19}H_{22}N_2O$	278.15, 266.15, 264.14, 254.15, 252.14, 250.12, 236.10, 224.11, 222.09, 210.09, 208.07, 198.09, 184.07, 144.08, 122.10	unknown	L4	<0.1
**P11**	108	8.47	$[M+H]^+$	299.17540	299.17532	0.28	9	$C_{18}H_{22}N_2O_2$	281.16, 252.14, 238.12, 226.12, 210.09, 198.09, 184.08, 174.09, 172.07, 160.07, 146.06, 140.11, 122.10, 108.08	unknown	L4	<0.1
**P13**	219	8.52	$[M+H]^+$	355.16523	355.16512	0.32	11	$C_{20}H_{22}N_2O_4$	/	unknown	L4	<0.1
**P17**	198	9.44	$[M+H]^+$	371.19653	371.19633	0.55	10	$C_{21}H_{26}N_2O_4$	210.06, 196.07, 174.09, 172.08, 160.07, 134.06, 122.09, 108.08, 107.07	unknown	L4	<0.1
**P24**	270	10.44	$[M+H]^+$	323.17540	323.17522	0.57	11	$C_{20}H_{22}N_2O_2$	305.16, 279.18, 222.13, 180.08, 168.08, 156.08, 122.09, 108.08	unknown	L4	<0.1
**P25**	10813	10.59	$[M+H]^+$	341.18597	341.18587	0.29	10	$C_{20}H_{24}N_2O_3$	323.17, 251.12, 236.14, 223.12, 196.10, 180.08, 166.06, 144.08, 108.08	unknown	L4	<0.1
**P47**	971	12.98	$[M+H]^+$	325.15467	325.15449	0.55	11	$C_{19}H_{20}N_2O_3$	/	unknown	L4	<0.1
**P58**	636	14.17	$[M+H]^+$	369.18088	369.18058	0.82	11	$C_{21}H_{24}N_2O_4$	337.15, 325.15, 308.14, 280.10, 239.12, 210.05,	unknown	L4	<0.1

Peak No.	FBMN node	RT (min)	Ion type	Precursor ion (calc.)	Precursor ion (exp.)	*Δ*ppm	DBE	Formula	Fragment ions	Annotation	Level ※	Average area (%)
									182.09, 166.09, 156.08, 122.10			
**P65**	237	14.71	$[M+H]^+$	311.21179	311.21156	0.74	9	$C_{20}H_{26}N_2O$	281.17, 251.13, 238.12, 212.15, 184.11, 170.10, 158.09, 144.08, 132.08, 130.06, 124.11, 112.11, 110.10,	unknown	L4	<0.1
**P85**	103	16.92	$[M+H]^+$	297.19614	297.19591	0.78	9	$C_{19}H_{24}N_2O$	279.19, 269.20, 250.15, 235.12, 222.12, 196.11, 182.09, 170.10, 156.08, 144.08, 130.06, 124.11	unknown	L4	<0.1
**P89**	364	17.38	$[M+H]^+$	353.18597	353.18588	0.25	11	$C_{21}H_{24}N_2O_3$	/	unknown	L4	<0.1
**P106**	857	19.83	$[M+H]^+$	283.18049	283.18033	0.57	9	$C_{18}H_{22}N_2O$	/	unknown	L4	<0.1
**P122**	56	21.61	$[M+H]^+$	399.19145	399.19125	0.50	11	$C_{22}H_{26}N_2O_5$	369.18, 339.17, 321.16, 307.14, 289.13, 279.15, 270.11, 230.08, 186.09, 172.08, 144.08, 108.08, 107.07, 106.06	unknown	L4	0.54
**P126**	358	22.02	$[M+H]^+$	337.15467	337.15441	0.77	12	$C_{20}H_{20}N_2O_3$	322.13, 305.13, 277.13, 263.12, 222.13, 144.08, 108.08	unknown	L4	<0.1
**P131**	194	22.38	$[M+H]^+$	353.14958	353.14949	0.27	12	$C_{20}H_{20}N_2O_4$	323.17, 293.13, 202.09, 170.10, 144.06, 124.08, 122.06, 108.08	unknown	L4	1.12
**P133**	762	22.70	$[M+H]^+$	309.15975	309.15971	0.14	11	$C_{19}H_{20}N_2O_2$	291.15, 281.16, 238.12, 186.09, 132.08, 108.08	unknown	L4	<0.1
**P153**	65	24.16	$[M+H]^+$	323.17540	323.17535	0.17	11	$C_{20}H_{22}N_2O_2$	291.15, 280.14, 249.14, 180.08, 156.08, 142.06, 122.10, 108.08, 106.06	unknown	L4	<0.1
**P155**	472	24.28	$[M+H]^+$	367.16523	367.16508	0.42	12	$C_{21}H_{22}N_2O_4$	/	unknown	L4	<0.1
**P159**	no	24.44	$[M+H]^+$	293.12845	293.12828	0.60	12	$C_{18}H_{16}N_2O_2$	278.10, 263.08	alstoscholarisines F☆	L1	<0.1
**P161**	86	24.47	$[M+H]^+$	323.17540	323.17531	0.29	11	$C_{20}H_{22}N_2O_2$	293.16, 263.15, 249.14, 234.13, 220.11, 202.08, 194.10, 180.10, 170.10, 144.08, 122.10	pleiocarpamine	L2	<0.1
**P175**	no	25.36	$[M+H]^+$	187.08659	187.08647	0.64	8	$C_{11}H_{10}N_2O$	159.09, 132.08, 130.06, 117.06, 115.05	1,2,3,4-tetrahydro-1-oxo-*β*-carboline▲	L2	<0.1
**P181**	no	25.90	$[M+H]^+$	339.20670	339.20657	0.40	10	$C_{21}H_{26}N_2O_2$	307.18, 138.12, 124.11	(+)-vincadifformine☆	L1	<0.1
**P185**	104	26.15	$[M+H]^+$	369.21727	369.21701	0.70	10	$C_{22}H_{28}N_2O_3$	337.19, 309.16, 246.09, 194.10, 168.08, 106.06	unknown	L4	<0.1

Peak No.	FBMN node	RT (min)	Ion type	Precursor ion (calc.)	Precursor ion (exp.)	*Δ*ppm	DBE	Formula	Fragment ions	Annotation	Level ※	Average area (%)
**P186**	193	26.19	$[M+H]^+$	327.17032	327.17023	0.27	10	$C_{19}H_{22}N_2O_3$	309.16, 280.12, 263.12, 184.07, 156.08	leuconolam	L2	<0.1
**P188**	no	26.21	$[M+H]^+$	499.20749	499.20732	0.35	13	$C_{26}H_{30}N_2O_8$	337.15, 267.11, 171.09, 144.08	strictosamide☆	L1	0.64
**P203**	296	27.22	$[M+H]^+$	399.19145	399.19139	0.15	11	$C_{22}H_{26}N_2O_5$	202.09, 136.08, 124.08, 112.04	unknown	L4	<0.1
**P204**	239	27.22	$[M+H]^+$	381.21727	381.21712	0.39	11	$C_{23}H_{28}N_2O_3$	335.18, 303.15, 275.15, 228.10, 144.08	unknown	L4	2.31
**P205**	563	27.49	$[M+H]^+$	311.17540	311.17531	0.34	10	$C_{19}H_{22}N_2O_2$	228.14, 56.04	leuconoxine★	L1	<0.1
**P207**	525	27.73	$[M+H]^+$	359.19653	359.19628	0.71	9	$C_{20}H_{26}N_2O_4$	341.19, 299.18, 254.15, 182.12, 154.12, 137.10, 109.10	unknown	L4	<0.1
**P208**	1139	27.83	$[M+H]^+$	387.19145	387.19133	0.31	10	$C_{21}H_{26}N_2O_5$	328.15, 297.16	unknown	L4	<0.1
**P213**	no	28.51	$[M+H]^+$	351.17032	351.17021	0.31	12	$C_{21}H_{22}N_2O_3$	244.11, 232.11, 206.09, 134.09, 120.08, 106.06, 56.05	scholarisine H☆	L1	<0.1
**P222**	694	32.12	$[M+H]^+$	325.15467	325.15448	0.58	11	$C_{19}H_{20}N_2O_3$	281.16	unknown	L4	<0.1
**P223**	26	33.70	$[M+H]^+$	295.18049	295.18046	0.10	10	$C_{19}H_{22}N_2O$	277.17, 266.14, 253.17, 248.13, 238.15, 171.09, 137.10, 109.10	unknown	L4	1.0
**P229**	1046	35.79	$[M+H]^+$	353.18597	353.18583	0.39	11	$C_{21}H_{24}N_2O_3$	281.13	unknown	L4	<0.1

★ Reference MIAs in FBMN; ☆ Reference MIAs out of FBMN; ▲ Confidential annotation MIAs in FBMN.

※ Annotation levels of MIAs: L1, confirmed by reference standard; L2, speculated by library spectrum match or diagnostic fragmentation evidence; L3, tentative classified candidate; L4, exact molecular formula.

References

[1] Liu, L.; Chen, Y. Y.; Qin, X. J.; et al. Antibacterial monoterpenoid indole alkaloids from Alstonia scholaris cultivated in temperate zone. *Fitoterapia*, 2015, 105, 160-164.

[2] Atta ur, R.; Asif, M.; Ghazala, M.; et al. Scholaricine, an alkaloid from Alstonia scholaris. *Phytochemistry*, 1985, 24, 2771-2773.

[3] Qin, X. J.; Zhao, Y. L.; Song, C. W.; et al. Monoterpenoid indole alkaloids from inadequately dried leaves of Alstonia scholaris. *Natural Products and Bioprospecting*, 2015, 5, 185-193.

[4] Yamauchi, T.; Abe, F.; Chen, R.-f.; et al. Alkaloids from the leaves of Alstonia scholaris in Taiwan, Thailand, Indonesia and the Philippines. *Phytochemistry*, 1990, 29, 3547-3552.

[5] Qin, X. J.; Zhao, Y. L.; Lunga, P. K.; et al. Indole alkaloids with antibacterial activity from aqueous fraction of Alstonia scholaris. *Tetrahedron*, 2015, 71, 4372-4378.

[6] Yuan, Y. X.; Guo, F.; He, H. P.; et al. Two new monoterpenoid indole alkaloids from Alstonia rostrata. *Natural Product Research*, 2018, 32, 844-848.

[7] Salim, A. A.; Garson, M. J.; Craik, D. J. New indole alkaloids from the bark of Alstonia scholaris. *Journal of Natural Products*, 2004, 67, 1591-1594.

[8] Chen, Y. Y.; Yang, J.; Yang, X. W.; et al. Alstorisine A, a nor-monoterpenoid indole alkaloid from cecidogenous leaves of Alstonia scholaris. T*etrahedron Letters*, 2016, 57, 1754-1757.

[9] Pinar, M. R., U.; Hesse, M.; Schmid, H. Tubotaiwin-N-oxid aus der wurzelrinde von conopharyngia johnstoni mitteilung über alkaloide. *Helvetica Chimica Acta*, 1972, 55, 2972-2974.

[10] Yang, X.-W.; Luo, X.-D.; Lunga, P. K.; et al. Scholarisines H–O, novel indole alkaloid derivatives from long-term stored Alstonia scholaris. *Tetrahedron*, 2015, 71, 3694-3698.

[11] Abe, F.; Chen, R.-F.; Yamauchi, T.; et al. Alschomine and Isoalschomine, New alkaloids from the Leaves of Alstonia scholaris. *Chemical & Pharmaceutical Bulletin*, 1989, 37, 887-890.

[12] Cai, X.-H.; Liu, Y.-P.; Feng, T.; et al. Picrinine-type alkaloids from the leaves of Alstonia scholaris. *Zhongguo Tianran Yaowu*, 2008, 6, 20-22.

[13] Burnell, R. H. M., J.D. Alkaloids of aspidosperma cuspa blake. *Phytochemistry*, 1968, 7, 2045-2051.

[14] Zhou, H.; He, H.-P.; Luo, X.-D.; et al. Three new indole alkaloids from the leaves of Alstonia scholaris. *Helvetica Chimica Acta*, 2005, 88, 2508-2512.

[15] Attaurrahman; Khanum, S. Strictamine-N-oxide from Rhazya-stricta. *Phytochemistry*, 1984, 23, 709-710.

[16] Feng, T.; Cai, X. H.; Zhao, P. J.; et al. Monoterpenoid indole alkaloids from the bark of Alstonia scholaris. *Planta Medica*, 2009, 75, 1537-1541.

[17] Bao, M.-F.; Zeng, C.-X.; Qu, Y.; et al. Monoterpenoid indole alkaloids from Alstonia rostrata. *Natural Products and Bioprospecting*, 2012, 2, 121-125.

[18] Zhong, X.-H.; Bao, M.-F.; Zeng, C.-X.; et al. Polycyclic monoterpenoid indole alkaloids from Alstonia rostrata and their reticulate derivation. *Phytochemistry Letters*, 2017, 20, 77-83.

[19] Zeches, M.; Ravao, T.; Richard, B.; et al. Some new vallesamine-type alkaloids. *Journal of Natural Products*, 1987, 50, 714-720.

[20] Yang, X.-W.; Qin, X.-J.; Zhao, Y.-L.; et al. Alstolactines A–C, novel monoterpenoid indole alkaloids from Alstonia scholaris. *Tetrahedron Letters*, 2014, 55, 4593-4596.

[21] Cai, X. H.; Du, Z. Z.; Luo, X. D. Unique monoterpenoid indole alkaloids from Alstonia scholaris. *Organic Letters*, 2007, 9, 1817-1820.

[22] Cheng, G. G.; Zhao, Y. L.; Zhang, Y.; et al. Indole alkaloids from cultivated Vinca major. *Tetrahedron*, 2014, 70, 8723-8729.

[23] Martin, S. F.; Hunter, J. E.; Benage, B.; et al. Unified strategy for synthesis of indole and 2-oxindole alkaloids. *Journal of the American Chemical Society*, 1991, 113, 6161-6171.

[24] Yang, X. W.; Yang, C. P.; Jiang, L. P.; et al. Indole alkaloids with new skeleton activating neural stem cells. *Organic Letters*, 2014, 16, 5808-5811.

[25] Pan, Z. Q.; Qin, X. J.; Liu, Y. P.; et al. Alstoscholarisines H-J, indole alkaloids from Alstonia scholaris: Structural evaluation and bioinspired synthesis of alstoscholarisine H. *Organic Letters*, 2016, 18, 654-657.

[26] Cai, X.-H.; Tan, Q.-G.; Liu, Y.-P.; et al. A cage-monoterpene indole alkaloid from Alstonia scholaris. *Organic Letters*, 2008, 10, 577-580.

[27] Waterman, P. G.; Zhong, S. Vallesiachotamine and Isovallesiachotamine from the Seeds of Strychnos Tricalysioides. *Planta Medica*, 1982, 45, 28-30.

[28] Dhooghe, L.; Mesia, K.; Kohtala, E.; et al. Development and validation of an HPLC-method for the determination of alkaloids in the stem bark extract of Nauclea pobeguinii. *Talanta*, 2008, 76, 462-468.

[29] Yang, X.-W.; Song, C.-W.; Zhang, Y.; et al. Alstoscholarisines F and G, two unusual monoterpenoid indole alkaloids from the leaves of Alstonia scholaris. *Tetrahedron Letters*, 2015, 56, 6715-6718.

[30] Smith, G. F.; Wahid, M. A. 760. The isolation of (±)- and (+)-vincadifformine and of (+)-1,2-dehydroaspidospermidine from Rhazya stricta. *Journal of the Chemical Society*, 1963, 4002-4004.

[31] Abe, F.; Yamauchi, T. Indole alkaloids from leaves and stems of Leuconotis eugenifolius. *Phytochemistry*, 1993, 35, 169-171.

[32] Pluskal, T.; Castillo, S.; Villar-Briones, A.; et al. MZmine 2: Modular framework for processing, visualizing, and analyzing mass spectrometry-based molecular profile data. *BMC Bioinformatics*, 2010, 11, e395

[33] Katajamaa, M.; Miettinen, J.; Oresic, M. MZmine: toolbox for processing and visualization of mass spectrometry based molecular profile data. *Bioinformatics*, 2006, 22, 634-636.

[34] Nothias, L. F.; Petras, D.; Schmid, R.; et al. Feature-based molecular networking in the GNPS analysis environment. *Nature Methods*, 2020, 17, 905-908.
